# Peptidoglycan-reshuffling proteins SCO0954, SCO1758, SCO4439, and SCO4440 modulate the formation of wall-deficient cells in *Streptomyces coelicolor* under hyperosmotic sucrose stress

**DOI:** 10.1038/s41598-025-15457-z

**Published:** 2025-09-01

**Authors:** Sergio Alonso-Fernández, Ignacio Gutiérrez-Del-Río, Felipe Lombó, María Teresa Fernández-Del-Campo-García, Eliseo Herrero-Hernández, Diego García-Gómez, Paula Díez, María Montes-Bayón, Gemma Fernández-García, Angel Manteca

**Affiliations:** 1 Department of Functional Biology, Microbiology Area, IUOPA and ISPA, Faculty of Medicine, Universidad de Oviedo, c/Julian Claveria 6, Oviedo, 33006 Spain; 2 Department of Analytical Chemistry, Nutrition and Food Science, University of Salamanca, Plaza de los Caídos s/n, Salamanca, 37008 Spain; 3 Department of Functional Biology, Immunology Area, Faculty of Medicine and Health Science, Universidad de Oviedo, Oviedo, 33006 Spain; 4 Department of Physical and Analytical Chemistry, Faculty of Chemistry and ISPA, Universidad de Oviedo, c/Julian Claveria 8, Oviedo, 33006 Spain

**Keywords:** *Streptomyces*, Cell division, EVs, Wall-deficient cells, S-cells, L-forms, Peptidoglycan, Antibiotic, Differentiation, Sporulation, Environmental microbiology, Antibiotics, Bacterial genes, Bacterial development, Bacterial genetics, Bacterial structural biology

## Abstract

Streptomycetes are biotechnologically valuable bacteria with complex cell division that produce extracellular vesicles (EVs), typically nanometre-sized but can reach 2.5 μm in diameter. *Streptomyces* also produce dividing wall-deficient L-forms (0.5–7 μm diameter) and, under hyperosmotic stress, non-dividing wall-deficient S-cells (3–4 μm diameter). The boundaries between EVs, L-forms and S-cells are not always clear, as large DNA-containing EVs can resemble small L-forms and S-cells in size. Both EVs and wall-deficient cells offer competitive advantages, such as inter-bacterial signalling, antibiotic transport, resistance and phage defence. However, their formation mechanisms remain poorly understood. We identified *sco1758* (*engA* GTPase), *sco0954* (methionine N-acetyltransferase), *sco4439* (D-Ala-D-Ala carboxypeptidase), and *sco4440* (GOLPH3-like) as important for wall-deficient cell formation in *Streptomyces coelicolor* under hyperosmotic sucrose conditions. Mutations in *sco4439* and *sco4440* increased tetra–tetra(Gly) and tetra(Gly)–penta(Gly) (4–3) peptidoglycan (PG) dimers, while *sco1758* affected only the former. Complementation reversed these changes. *sco0954* overexpression enhanced PG-associated methionine acetylation and oxidation. Our findings suggest that PG dimerisation and methionine modification may contribute to the formation of wall-deficient cells under hyperosmotic sucrose stress. Further research is required to elucidate how SCO1758, SCO0954 and SCO4439/40 modulate PG architecture and to evaluate their potential to promote EV production for biotechnological applications.

**Supplementary Information:**

The online version contains supplementary material available at 10.1038/s41598-025-15457-z.

## Introduction

Streptomycetes are biotechnologically important bacteria that produce nearly two-thirds of clinically used bioactive secondary metabolites, including antibiotics, antitumour agents, and immunosuppressants^1,2^. Their complex developmental cycle involves programmed cell death, hyphal differentiation, and sporulation^3,4^. Cell division is unusually intricate, as vegetative and sporulation septa are differentially regulated: proteins such as FtsI, FtsL, FtsW, SsgA/B, CrgA, DynA/B, and SepH are required for sporulation but not vegetative division^5–12^, while SepX is specific to vegetative septation^13^. The tubulin-like GTPase FtsZ functions in both, recruiting the divisome^14–16^. *Streptomyces* is the only known bacterium in which *ftsZ* mutations do not abolish viability, although development is altered^14^.

*Streptomyces* cell biology is further complicated by the presence of non-replicative extracellular vesicles (EVs)^17–20^, which may carry proteins and metabolites, including antibiotics. While some lack DNA^21^, others may contain an entire chromosome, suggesting a role in genome evolution^19^. Vesicles have been linked to programmed cell death^11,22^ and division^12^. In addition, *Streptomyces* produce L-forms, wall-deficient cells capable of dividing independently of the FtsZ machinery^23–25^. Recently, non-dividing wall-deficient cells induced by hyperosmotic stress, termed S cells, have been identified in actinomycetes such as *Kitasatospora viridifaciens*, *Streptomyces griseus* and *Streptomyces venezuelae*^26^. These cells are induced by hyperosmotic stress in media supplemented with 0.64 M sucrose, 0.6 M NaCl or 1 M sorbitol^26^.

EVs, S-cells, and L-forms may confer adaptive benefits. L-forms of pathogenic bacteria are associated with persistent infections, resistance to cell wall-targeting antibiotics^27–29^, and phage protection^30,31^. L-forms in *K. viridifaciens* can take up extracellular DNA via endocytosis-like processes contributing to horizontal gene transfer^32^. EVs may contribute to defence, for example by transporting β-lactamases in *Staphylococcus aureus* or acting as phage decoys^33^. Nonetheless, the biological roles of EVs, S-cells, and L-forms in *Streptomyces* remain largely unclear.

The size of *Streptomyces* L-forms (0.5–7 μm in diameter)^24,25^ and actinomycete S-cells (3–4 μm in diameter)^26^ is significantly larger than that reported for most *Streptomyces* EVs (0.08–0.4 μm in diameter)^17–20^, but more comparable to EVs observed in *S. venezuelae* (2.5 μm in diameter)^21^. Their distinct size, support their classification as separate entities, although the boundaries between them are not always well defined, as the largest DNA-containing EVs can be similar in size with the smallest L-forms and S-cells. According to Welsh et al.^34^, EVs are lipid bilayer-bound particles released from cells. As L-forms divide, they fall outside this definition, while S-cells can be considered large EVs formed under hyperosmotic stress^26^.

EV formation in Gram-negative bacteria is relatively well understood, involving the outer membrane and up to 150 genes^18^. In contrast, Gram-positive EV biogenesis remains elusive. Heat-inactivated gram-positive bacteria do not produce EVs, indicating a dependence on metabolic activity^35^, and some regulatory genes have been implicated in modulating EV formation in these organisms. The sigma factor *sigB* and *covRS* influence EV synthesis in *Listeria monocytogenes* and *Streptococcus pyogenes*, respectively^35^. In *Streptomyces venezuelae* and *Streptomyces lividans*, EVs form at hyphal tips, likely due to mechanical stress from weakened apical walls^20,21^. In *S. coelicolor*, a contractile tail-like system (*sco4256*–*4258*) regulates vesicle formation during cell death^22^.

L-form division has been reported to depend on membrane fluidity and excess intracellular membranes^36^, as shown in *E. coli*^37^, *L. monocytogenes*^38^, and *B. subtilis*^39^, enabling passive, biophysical division^40^. Similar behaviour is seen in synthetic giant lipid vesicles^41^. Despite their passive nature, regulatory mechanisms affecting cell wall synthesis (e.g., peptidoglycan release) and intracellular membrane production are likely involved. Indeed, mutations affecting L-form formation have been found, as the *murE* gene repression in *B. subtilis*^23^ and *mraY* and *ftsQ* mutations in *E. coli*^37^.

S-cells are, by definition, induced by hyperosmotic stress in some actinomycetes^26^. While L-forms are not restricted to such conditions, they can also be triggered by hyperosmotic stress in actinomycetes^26^. The molecular regulation of S-cell and L-form formation remains unclear, but evidence for genetic control is growing. For example, *filP* mutation in *K. viridifaciens* prevents S-cell formation^42^. The stomatin-like protein StlP, involved in polar growth, was recently linked to the emergence of wall-deficient cells under hyperosmotic stress in *S. coelicolor*^43^, a strain previously not known to form S-cells or L-forms^24–26^, but which can produce EVs^17,18,44^.

In this study, we further explore the genetic modulation of S-cell and L-form formation in *Streptomyces coelicolor*. We identified *sco1758* (EngA GTPase), *sco0954* (methionine N-acetyltransferase), *sco4439* (D-Ala-D-Ala carboxypeptidase), and *sco4440* (GOLPH3-like), whose mutations alter peptidoglycan (PG) muropeptide composition and affect the redox and acetylation states of PG-bound methionine. These changes correlate with the emergence of wall-deficient cells under hyperosmotic sucrose stress (S-cells in *sco0954* and *sco4439/40*, and both S-cells and L-forms in *sco1758*).

## Results

### The *sco1760::Tn5*, and *sco4439/40::Tn5062* knockout mutants, as well as *S.coelicolor* overexpressing *sco0954*, exhibit hyphal bulging and the formation of large distinct round cells under conditions of hyperosmotic sucrose stress

**Fig. 1:**
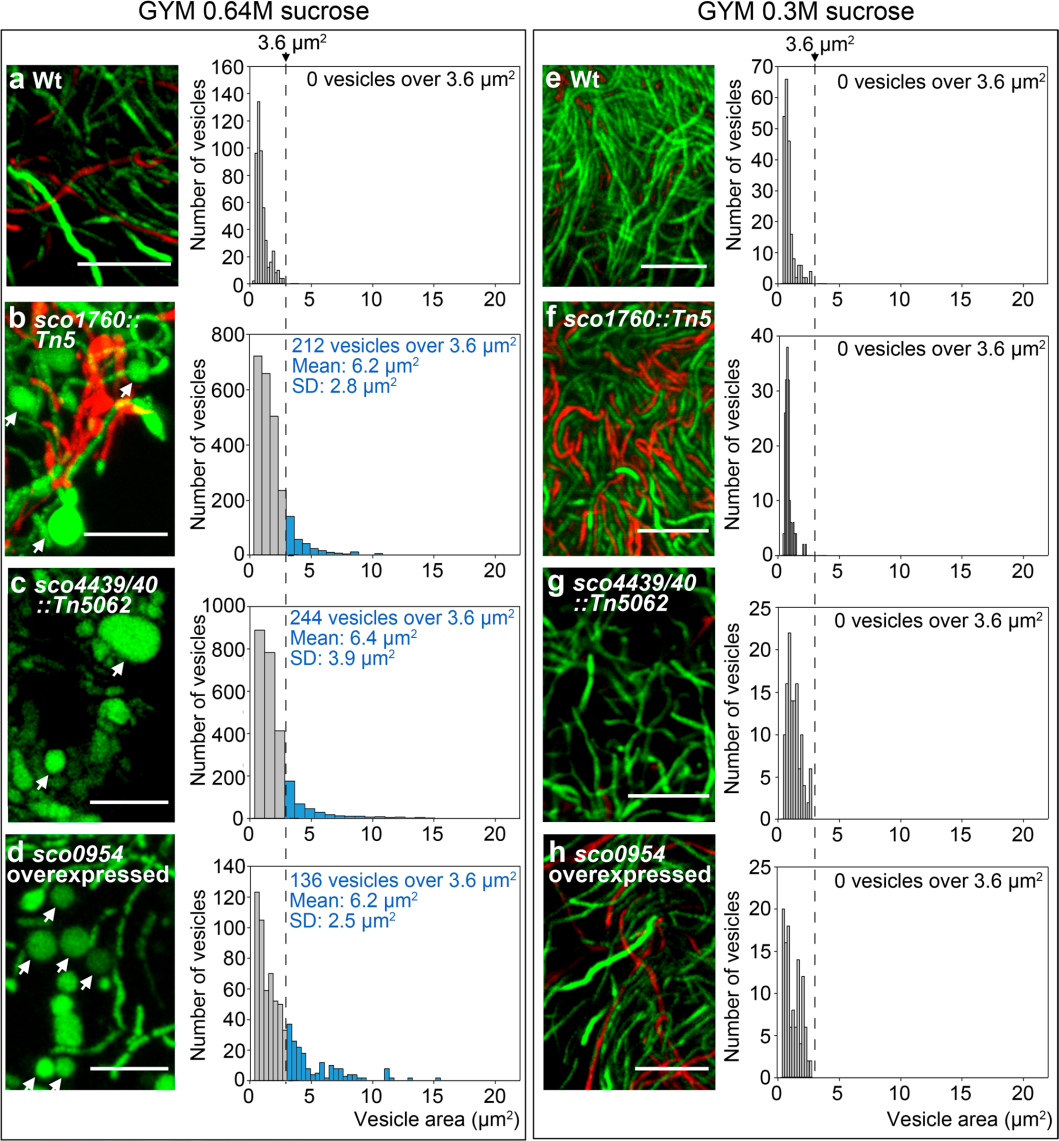
Formation of wall-deficient cells and extracellular vesicles (EVs) under sucrose-induced hyperosmotic stress in the *sco1760::Tn5*, *sco4439/40::Tn5062*, and *sco0954* knockout mutants, as well as in *S. coelicolor* overexpressing *sco0954*, grown on GYM medium supplemented with 0.64 M sucrose. Images correspond to confocal laser-scanning fluorescence microscopy of hyphae stained with SYTO9 and PI (DNA stains). Histograms represent EVs sizes (areas): grey bars correspond to the *S. coelicolor* wild-type strain (negative control, not producing EVs), used to establish the threshold area for EVs identification; blue bars represent EVs with an area above the 3.6 μm² threshold in mutants grown on GYM medium supplemented with 0.64 M sucrose. **(a–d) **Cultures on GYM medium supplemented with 0.64 M sucrose. **(e–h)** Cultures on GYM medium supplemented with 0.64 M sucrose. Representative images from at least three biological replicates are shown. Arrows indicate EVs and sucrose-driven hyperosmotic-stress-induced cells. Scale bars: 8 μm.

The *sco1760::Tn5* mutant was generated in this study using the transposon-based random mutagenesis method of Xu et al.^45^. The phenotypes of 1,000 random mutants were analysed macroscopically (colony morphology, and production of undecylprodigiosin [red] and actinorhodin [blue]), and microscopically, using vital, membrane and cell-wall stains). The *SCO1760::Tn5* mutant exhibited pronounced hyphal bulging and the formation of large extracellular round cells that contain DNA (arrows in Fig. 1b), most of which were viable (SYTO9 green staining; see methods).

The *sco4439/40::Tn5062* mutant, constructed previously^46^, lacks SCO4439, a D-Ala-D-Ala carboxypeptidase; this increases peptidoglycan cross-linking and causes notable spore swelling during germination^46^. This mutant also exhibited hyphal bulging and the formation of large cells in medium amended with 0.64 M sucrose (Fig. 1c).

*sco0954* was identified in this study via RNA-seq comparing an *ftsZ* mutant^14^ with the *S. coelicolor* wild type. Given its marked upregulation in the mutant (Supplementary Table S1), we hypothesised that it may be involved in the FtsZ-independent division of L-forms. We overexpressed *sco0954* in the wild-type strain to test this, observing a phenotype similar to the *sco1760::Tn5* and *sco4439/40::Tn5062* mutants, S-cell formation in hyperosmotic sucrose conditions (Fig. 1d).

No differences in viability were observed among the mutants, however, some of the large cells formed as well as the bulging hyphae, were dead (as indicated by red PI staining in Fig. 1; see additional images in Supplementary Fig. S1), perhaps reflecting an instability of these structures. Hyphal bulging and distinct large cells were not observed on 0.3 M sucrose (Fig. 1, right panels), a concentration typically used in laboratory media to protect wall-deficient cells from lysis (see examples of media designed to preserve protoplast integrity in Kieser et al.^47^.

Overexpression of *sco0954* and mutations in *sco1760* and *sco4439/40* induce wall-deficient S-cells under hyperosmotic sucrose stress in Streptomyces coelicolor.

### Overexpression of *sco0954* and mutations in *sco1760* and *sco4439/40* induce wall-deficient S-cells under hyperosmotic sucrose stress in *Streptomyces coelicolor*.

**Fig. 2:**
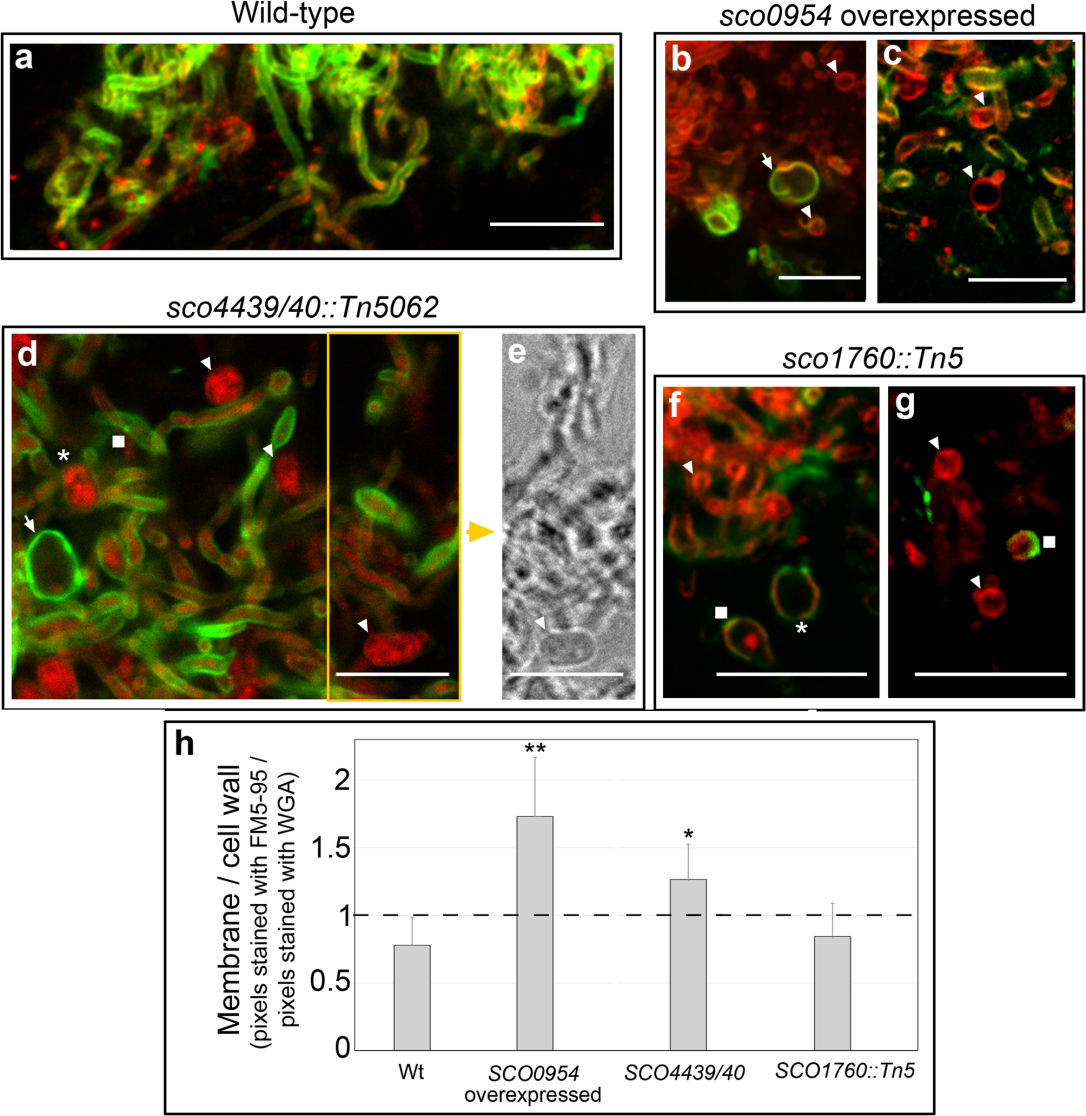
Cells formed under sucrose-induced hyperosmotic stress in the *sco1760::Tn5* and *sco4439/40::Tn5062* mutants, as well as in the wild-type strain overexpressing *sco0954*, lack a cell wall. **(a–g)** Confocal laser-scanning microscopy images of cultures grown on GYM medium supplemented with 0.64 M sucrose and stained with FM5-95 (red membrane stain) and Alexa Fluor 488–WGA (green cell wall stain). **(h)** Membrane-to-cell-wall abundance quantified as the ratio between pixels stained with WGA and FM5-95. Asterisks indicate statistically significant differences compared to the wild-type strain: *p* < 0.05, *p < 0.01, **p < 0.001. Arrowheads indicate cells without detectable cell walls; asterisks denote cells with very thin cell walls; arrows indicate cells with thick peptidoglycan (PG) walls; and squares indicate cells with cell wall patches. Representative images from at least three biological replicates are shown. Scale bars represent 8 μm.

We next examined whether the cells induced under hyperosmotic sucrose conditions observed here, like S- and L-cells, lack a cell wall. To do this, we used Alexa Fluor 488-WGA (green, cell wall) and FM5-95 (red, membrane) staining (Fig. 2). No large distinct round cells were detected in the *S. coelicolor* wild type (Fig. 2a). In contrast, all mutants grown on GYM medium with 0.64 M sucrose produced large distinct round cells with membranes (red) without detectable walls (green) (arrowheads in Fig. 2). Consequently, these cells fit the definition of EVs^34^. More precisely they are S-cells, i.e. wall-deficient cells induced under hyperosmotic stress (0.64 M sucrose) that are unable to divide. We also observed cells with thin walls (asterisks), thick walls (arrows), or localised wall patches (squares), which may represent remnants of original walls or sites of peptidoglycan regeneration. Some vesicles displayed excess internal FM5-95 staining.

**Fig. 3:**
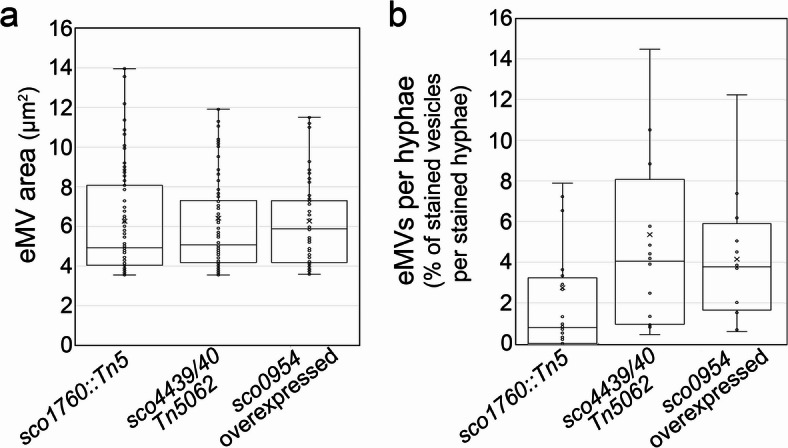
Area and abundance of extracellular vesicles (EVs) formed under sucrose-induced hyperosmotic stress in the *sco1760::Tn5*, *sco4439/40::Tn5062*, and *sco0954* knockout mutants, as well as in *S. coelicolor* overexpressing *sco0954*, grown on GYM medium supplemented with 0.64 M sucrose. **(a)** Osmotic-stress-induced cell/EVs area. **(b)** Osmotic-stress-induced EVs abundance. Box and whisker elements: centreline, median; box limits, upper and lower quartiles; whiskers, 1.5x interquartile range.

We quantified the membrane-to-wall ratio from confocal images using Fiji^48^, calculating the FM5-95 to WGA pixel ratio across at least three biological replicates (see Supplementary Fig. S1 for representative images). In wild-type cultures grown on 0.64 M sucrose, this ratio was slightly below 1, reflecting more peptidoglycan staining, as expected, as the cell wall is thicker than the membrane (Fig. 2a). In contrast, the *sco0954*-overexpressing strain and the *sco4439/40::Tn5062* mutant showed significantly higher ratios (> 1), indicating membrane enrichment (Fig. 2h). The *sco1760::Tn5* mutant did not differ significantly from wild type, likely due to its lower abundance of stress-induced vesicles (see next paragraph, Fig. 3b), with most stained pixels corresponding to hyphae with thick walls rather than wall-deficient cells.

### The *sco1760::Tn5*, and *sco4439/40::Tn5062* knockout mutants, as well as *S. coelicolor* overexpressing *sco0954*, produce S-cells ranging from 4 to 14 µm^**2**^ (corresponding to diameters of 2.2 to 4.2 μm)

Extracellular S-cell sizes were quantified from confocal images using Fiji^48^. *S. coelicolor* wild-type cultures, which do not form EVs, defined a 3.6 μm² area cut-off (grey bars in Fig. 1 histograms), with smaller areas corresponding to minor hyphal thickenings (Fiji quantifies any round structure, including small hyphal thickenings occasionally observed in wild-type hyphae, see images in Fig. S1). All mutants produced EVs exceeding this threshold on 0.64 M sucrose but not on 0.3 M (blue bars, Fig. 1), indicating that these structures correspond to S-cells. Most vesicles ranged from 4 to 14 μm² (diameters of 2.2 to 4.2 μm) (Fig. 3a), consistent with reported sizes of *Streptomyces* L-forms (0.5–7 μm diameter; 0.2–38.4 μm² area)^24,25^, and *K. viridifaciens* S-cells (5.14 μm diameter; 20.73 ± 11.53 μm² area) and L-forms (3 μm diameter; 7.06 ± 5.87 μm² area)^26^. These sizes were larger than those from *Streptomyces* EVs (0.02 to 2.5 μm in diameter, 0.005–4.9 μm²)^17,21^, although they overlapped in the 4 to 4.9 μm² range.

Extracellular vesicle abundance was estimated from confocal images by calculating the percentage of SYTO9/propidium iodide-stained hyphal area occupied by vesicles (Fig. 3b; see Methods). Vesicle abundance in *sco4439/40::Tn5062* and *sco0954*-overexpressing strains ranged from 2 to 14%, whereas *sco1760::Tn5* showed lower levels (0–8%) (Fig. 3b), which as stated in the previous paragraph, corresponds with a lower membrane-to-wall ratio in this mutant (Fig. 2h).

### Extracellular vesicle formation induced by hyperosmotic sucrose stress in *sco1760::Tn5* and *sco4439/40::Tn5062* mutants is complemented by *sco1758* and *sco4439/40*, respectively

**Fig. 4:**
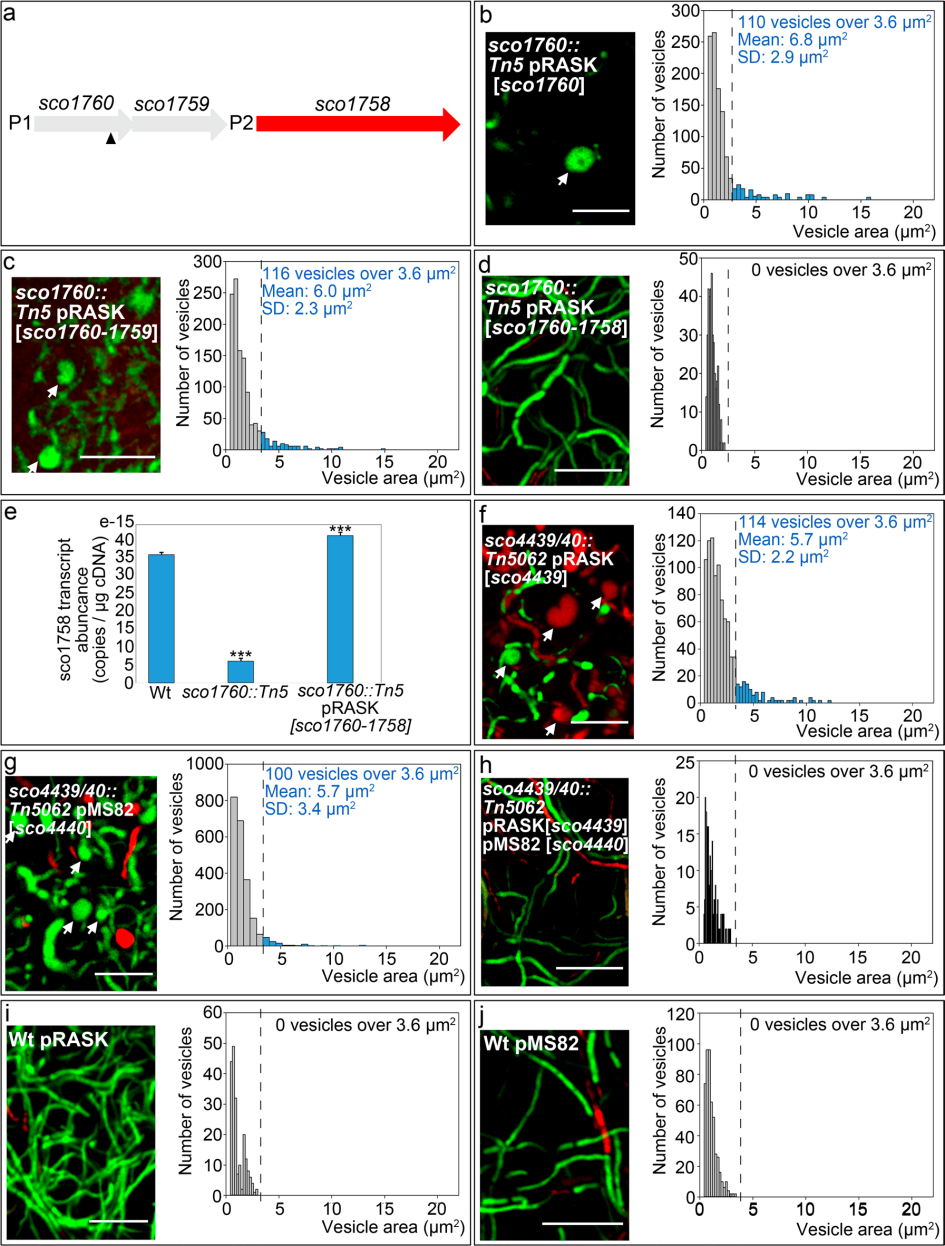
Formation of extracellular vesicles (EVs) under sucrose-induced hyperosmotic stress in the *sco1760::Tn5*, *sco4439/40::Tn5062*, and *sco0954* knockout mutants is complemented by *sco1758* and *sco4439/40*. Images and histograms are as described in Fig. 1. **(a)** Genetic region disrupted by the Tn5 transposon; the arrowhead indicates the insertion site. P1 and P2 denote the two promoters identified by Jeong et al.^49^. **(b-d)** Images and histograms of the mutants **(e)** *SCO1758* gene expression in 48-hour GYM solid cultures. **(f-h)** Images and histograms of the complemented mutant strains. **(i, j)** Images and histograms of the *S.coelicolor* wild-type strain harbouring the empty pRASK and pMS82 plasmids. All cultures were grown on GYM medium supplemented with 0.64 M sucrose. Asterisks indicate statistically significant differences compared to the wild-type strain: *p* < 0.05, *p* < 0.01, **p < 0.001. Arrows indicate EVs and sucrose-driven hyperosmotic stress-induced cells. Scale bars: 8 μm.

The Tn5 transposon in the *sco1760::Tn5* mutant is inserted at chromosomal position 1,881,109, near the end of the *sco1760* ORF (triangle, Fig. 4a), potentially affecting expression of downstream *sco1759–58* (Fig. 4a). To pinpoint the gene(s) responsible for the phenotype, we performed complementation with various *sco1760–58* combinations. Two promoters in this region, at positions 1,881,810 and 1,880,358 (P1 and P2, Fig. 4a), were previously identified by Jeong et al.^49^. Three complementation plasmids were constructed: one with *sco1760* alone; one with *sco1760* and *sco1759*; and one with *sco1760*, *sco1759*, and *sco1758*. All included upstream sequences covering the P1 promoter and were cloned into the integrative ΦC31 vector pRASK^50^.

Neither *sco1760* alone nor *sco1760* with *sco1759* restored the wild-type phenotype (absence of EVs under hyperosmotic sucrose stress) (Fig. 4b, c; blue bars; see Supplementary Fig. S1 for more images). Only the construct containing all three genes restored the wild-type phenotype (Fig. 4d), identifying *sco1758* as being involved in EV formation under hyperosmotic sucrose stress. However, *sco1760* and/or *sco1759* may also contribute to the *sco1760::Tn5* mutant phenotype.

Transcript analysis revealed a sevenfold decrease in *sco1758* expression in the *sco1760::Tn5* mutant versus wild type, while expression was restored in the strain complemented with *sco1760-1758* (Fig. 4e), which also rescued the phenotype (Fig. 4d), supporting *sco1758*’s role in EVs formation.

In the *sco4439/40::Tn5062* mutant, EVs formation under hyperosmotic sucrose stress was suppressed only when both *sco4439* and *sco4440* were co-expressed (Fig. 4f–h). Control plasmids pMS82^51^ and pRASK^50^ had no effect (Fig. 4i, j).

### The ***sco1760::Tn5*** mutant forms hyperosmotic sucrose stress-induced, division-capable cells (L-forms) when grown on GYM medium supplemented with 0.64 M sucrose

**Fig. 5:**
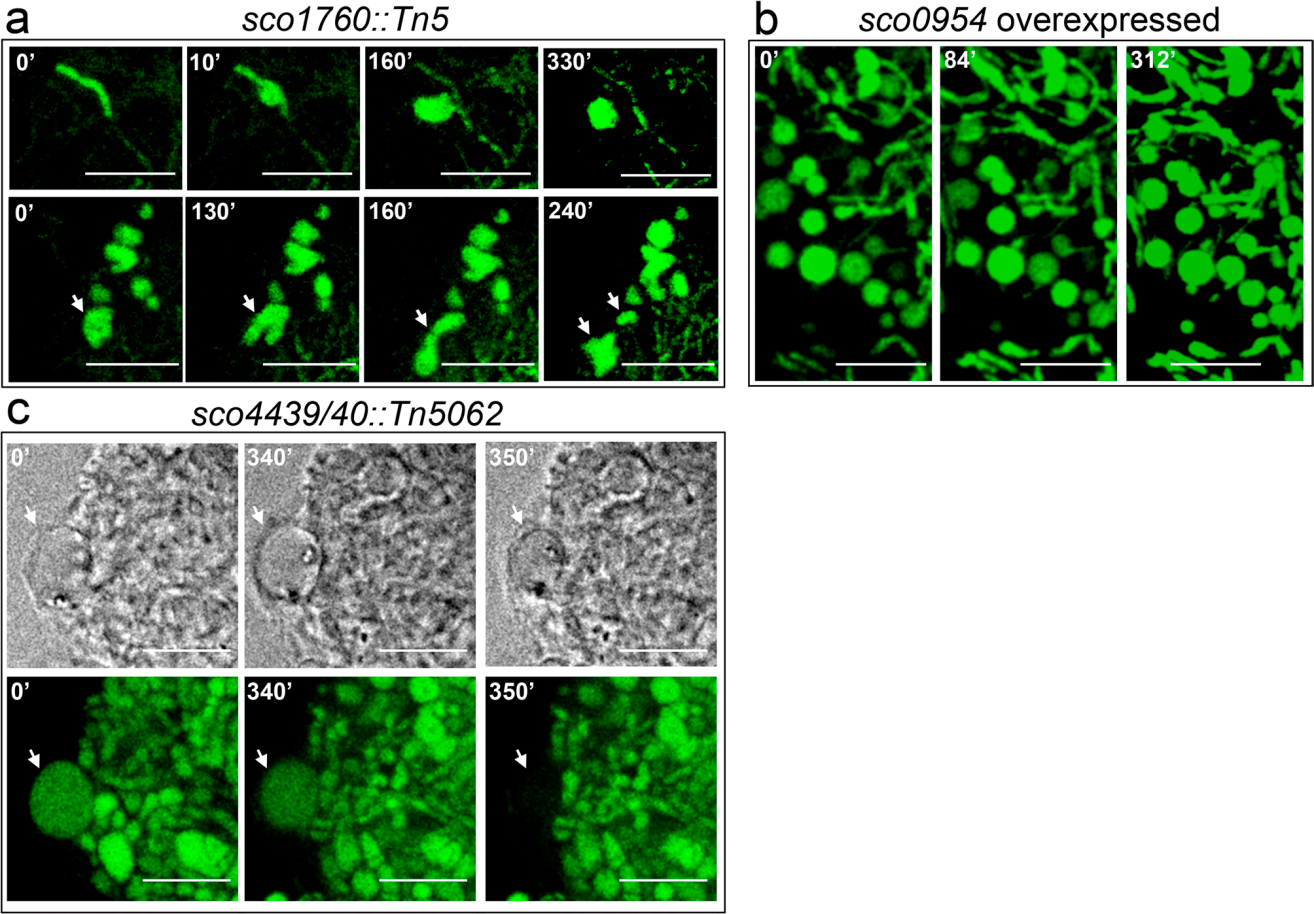
Dynamics of extracellular vesicles (EVs) induced by sucrose-driven hyperosmotic stress in GYM cultures supplemented with 0.64 M sucrose. **(a)** *sco1760::Tn5* mutant. **(b)** Wild-type strain overexpressing *sco0954*. **(c)** *sco4439/40::Tn5062* mutant (upper images in phase-contrast mode). Time-lapse imaging was initiated in 48-hour cultures, once EVs induced by sucrose-driven hyperosmotic stress had formed. Cultures were stained with SYTO-9 (green, DNA stain). Arrows indicate S-cells and L-forms. Time points (in minutes) are indicated; time 0 marks the start of time-lapse imaging (i.e. a 48-hour culture). Scale bars represent 8 μm.

We next examined the dynamics of osmotic stress-induced vesicles using time-lapse microscopy. Observations began at 48 h on GYM medium with 0.64 M sucrose, when vesicles were already present in all mutants. Samples were stained with SYTO-9 (green; DNA stain) (Fig. 5; Movies S1–S3).

In the *sco1760::Tn5* mutant, we observed cells forming as lateral evaginations from hyphae, producing distinct round vesicles clearly separated from the original hyphae (Fig. 5a, upper panels; Movie S1). We also observed chromosomal division (indicated by arrows in Fig. 5a, lower panels; Movie S1) and the separation of two independent chromosomes, which appear to be two independent cells at the 240-minute time point (Fig. 5a, lower panels). These events resemble the L-forms described by Ramijan et al.^26^ in *Kitasatospora viridifaciens*, which arise under osmotic stress and are capable of division.

L-forms were not seen in the *sco4439/40::Tn5062* mutant or in the *S. coelicolor* strain overexpressing *sco0954* (Fig. 5b–c). Both strains produced stable vesicles resembling the S-cells defined by Ramijan et al.^26^ in 48-hour GYM cultures supplemented with 0.64 M sucrose, corresponding to the aerial hyphae stage preceding sporulation (Fig. 5b–c; Movies S2–S4). Some EVs in the *sco4439/40::Tn5062* mutant lysed (see the large cell indicated by an arrow in Fig. 5c). Based on these data, we cannot currently determine the dynamics of EV formation in these two mutants.

### The SCO1758 EngA GTPase, SCO0954 methionine N-acetyltransferase, SCO4439 D-Ala-D-Ala carboxypeptidase, and SCO4440 GOLPH3-like protein are highly conserved among *Streptomyces* species

In order to try to understand the mechanism by which mutations in *sco1758*, *sco4439/40*, and the overexpression of *sco0954* lead to the formation of wall-deficient cells under hyperosmotic sucrose stress conditions, we studied the homology and predicted functions of these proteins across six model *Streptomyces* strains (*S. lividans*, *S. avermitilis*, *S. venezuelae*, *S. scabies*, *S. griseus*, and *S. clavuligerus*).

**Fig. 6:**
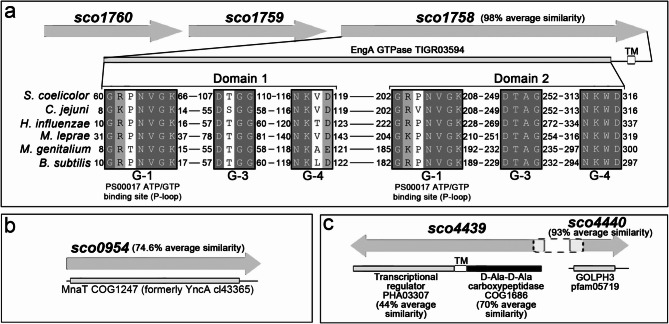
Homology of the SCO1758, SCO0954, SCO4439, and SCO4440 proteins. **(a)** Alignment of SCO1758 with the prototype bacterial EngA GTPases^55^, showing two GTP-binding domains, the G-1, G-3, and G-4 motifs, and the P-loop characteristic of EngA GTPases. **(b)** Conserved domain and protein conservation of SCO0954. **(c)** Conserved domains and protein conservation of SCO4439 and SCO4440. Conserved domain database accession numbers are provided, along with the average similarity of the protein orthologues across six model *Streptomyces* species: *S. lividans*, *S. avermitilis*, *S. venezuelae*, *S. scabies*, *S. griseus*, and *S. clavuligerus*.

SCO1758 carries a putative EngA GTPase domain (CDD: TIGR03594), sharing 98% similarity across six model *Streptomyces* species (*S. lividans*, *S. avermitilis*, *S. venezuelae*, *S. scabies*, *S. griseus*, *S. clavuligerus*). EngA GTPases, part of a conserved GTPase superfamily, act as molecular switches regulating key processes such as chromosome segregation, cell division, and ribosome stability in *E. coli*^52–54^. Their role in wall-deficient cell formation is plausible; however, to the best of our knowledge, no study to date has described an effect of EngA GTPases on the formation of wall-deficient cells. The prototype EngA GTPases from *C. jejuni*, *B. subtilis*, *H. influenzae*, *M. genitalium*, and *M. leprae* are characterised by two tandem GTP-binding domains, each with G-1, G-3, and G-4 motifs^55,56^, which are also present in SCO1758 (Fig. 6a). The G-1 motif, a flexible loop found in many nucleotide-binding proteins, interacts with phosphate groups (Prosite: PS00017).

*sco0954* encodes a 160-residue protein with 75% average similarity in *Streptomyces* and contains a GCN5-related acetyltransferase (GNAT) MnaT domain (CDD: COG1247) (Fig. 6b). This domain defines the MddA subfamily of GNATs, first characterised in *Salmonella enterica*^57^. GNATs acetylate diverse molecules—amino acids, polyamines, antibiotics—modulating key pathways^58^. The *S. enterica* MddA specifically acetylates oxidative methionine derivatives, blocking their uptake and toxicity^57^.

SCO4439 contains a D-Ala-D-Ala carboxypeptidase domain (70% similarity) and a putative transcriptional regulator domain (44%), separated by a transmembrane region^46^. SCO4440 is highly conserved (93% similarity) and harbours a GOLPH3 (GPP34) domain (CDD: pfam05719) (Fig. 1c). Eukaryotic GOLPH3 proteins are conserved elements of the trans-Golgi network, directing protein trafficking via vesicle sorting^59^. GOLPH3-like proteins have not been previously described in prokaryotes. Human GOLPH3 (NP_071413.1) shares > 64% identity with animal homologues, and 39.73% with *S. cerevisiae*^59^, whereas SCO4440 shows only 27% identity with the human protein. Notably, *sco4439–sco4440* synteny is conserved in all six model *Streptomyces* species (Fig. 1c). A search of the NCBI gene database (15 February 2023) identified 218 bacterial species encoding GOLPH3-domain proteins, 129 of which were actinomycetes (Supplementary Table S2).

### Methionine acetylation and oxidation are significantly increased in peptidoglycan-anchored peptides of *Streptomyces coelicolor* overexpressing *sco0954*

As indicated in the previous paragraph, SCO0954 contains an MnaT domain (Fig. 6b), characteristic of the MddA family of methionine sulfoximine/L-methionine sulfone acetyltransferases. In *Salmonella enterica*, the MddA prototype acetylates extracellular oxidised methionine at its amino terminus, thereby preventing its uptake and avoiding cytosolic toxicity caused by oxidative damage^57^. We hypothesise that, analogous to *S. enterica* MddA, SCO0954 may acetylate extracellular methionine. While the primary peptidoglycan (PG) scaffold lacks methionine, sortases attach peptides to PG^60^, and the N-terminal methionine of these peptides could potentially be acetylated by SCO0954.

Quantifying acetylated methionine is challenging, as the most common method for amino acid quantification, o-phthalaldehyde derivatisation, is ineffective, since it targets the amino group, which is blocked by the acetyl moiety^61^. Moreover, acid hydrolysis, which is the most used method to release free amino acids, removes the acetyl group from methionine and is therefore unsuitable (Supplementary Fig. S2). To address these issues, we developed a method involving pronase digestion to release amino acids from PG samples, followed by hydrophilic interaction liquid chromatography (HILIC) to quantify acetylated methionine without derivatisation (see Methods). We measured levels of methionine (M) and N-acetylmethionine (Ac-M), as well as their oxidised forms: M sulfone, M sulfoxide, Ac-M sulfone, and Ac-M sulfoxide.

**Fig. 7:**
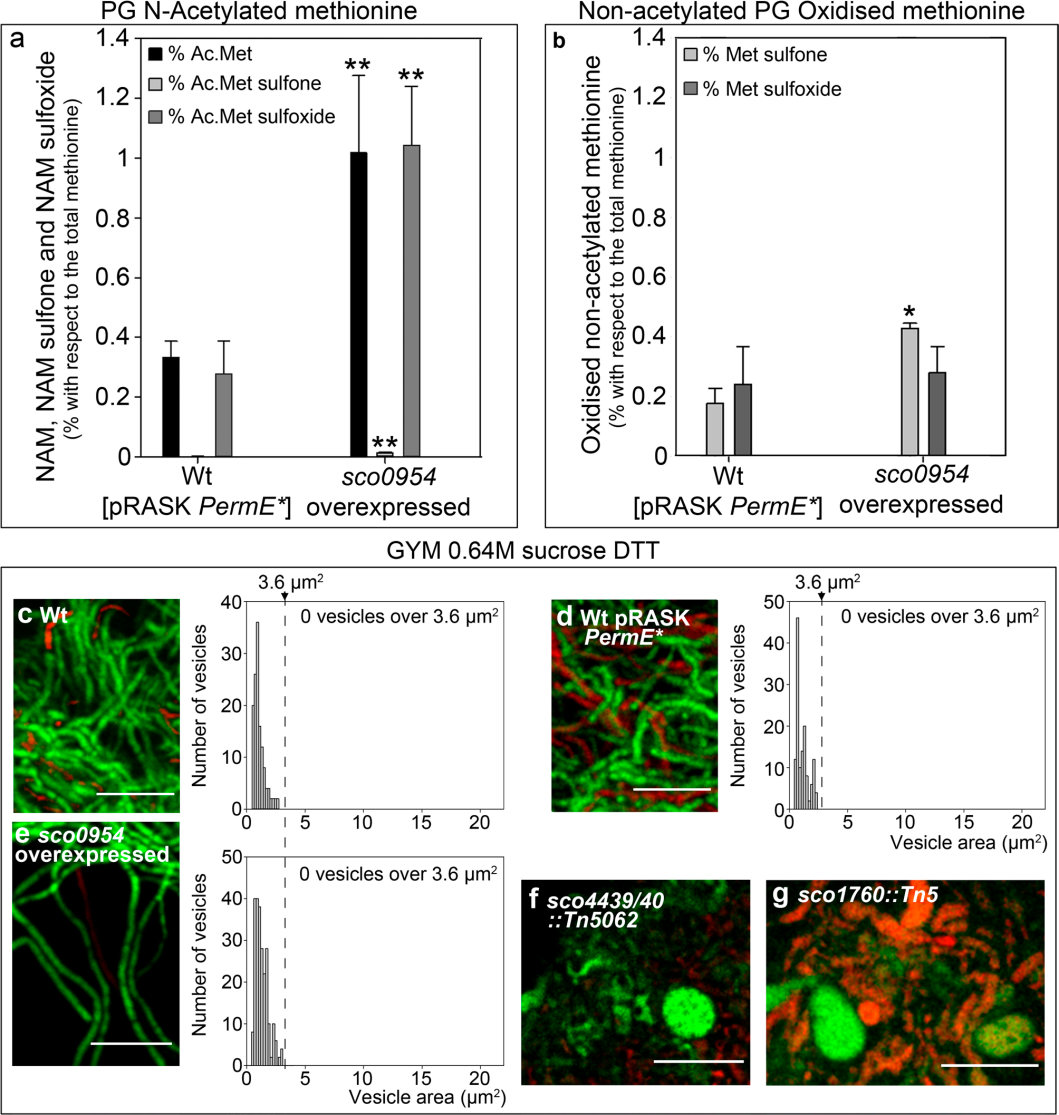
Acetylated/oxidised methionine in PG-associated peptides, and the effect of dithiothreitol on the inhibition of EVs formation, in the *S. coelicolor* strain overexpressing *sco0954*. **(a)** Levels of acetylated PG-associated methionine in the *S. coelicolor* strain overexpressing *sco0954*, compared to the *S. coelicolor* wild-type strain carrying the empty pRASK-PermE* plasmid. **(b)** Levels of non-acetylated, oxidised PG-associated methionine. **(c–e)** Absence of S-cells induced by sucrose-driven hyperosmotic stress in GYM medium supplemented with 0.64 M sucrose and 5 mM DTT, in the *S. coelicolor* strain overexpressing *sco0954*, and in the wild-type control strain with and without pRASK-PermE*. **(f)** Presence of hyperosmotic-stress induced cells in the *sco4439/40::Tn5062* mutant in GYM medium supplemented with 0.64 M sucrose and 5 mM DTT. **(g)** Presence of hyperosmotic-stress induced cells in the *sco1760::Tn5* mutant under the same conditions. Images and histograms are as in Fig. 1. Asterisks indicate statistically significant differences (*p* < 0.05, *p < 0.01, **p < 0.001). Error bars indicate SD.

The *S. coelicolor* strain overexpressing *sco0954* exhibited a marked increase in Ac-M and Ac-M sulfoxide levels, while Ac-M sulfone was only modestly elevated, relative to the wild-type strain carrying the empty pRASK-PermE* plasmid (Fig. 7a). Non-acetylated M sulfone was slightly elevated in the *sco0954*-overexpressing strain, whereas non-acetylated M sulfoxide levels remained comparable to the control strain (Fig. 7b). Overall, acetylated oxidised methionine species were more abundant than their non-acetylated counterparts, especially in the strain overexpressing *sco0954* (compare Fig. 7a and b).

These findings are consistent with SCO0954 possessing methionine acetyltransferase activity, potentially targeting oxidised methionine—particularly M sulfoxide, whose acetylated derivative showed the greatest increase. Nonetheless, further studies are required to fully elucidate the mechanism by which SCO0954 enhances levels of Ac-M and Ac-M sulfoxide in PG. As discussed below, these alterations in PG-associated peptides, may contribute to induction of wall-deficient cell formation.

### Dithiothreitol inhibits wall-deficient cell formation in *S. coelicolor* overexpressing *sco0954*

Having found increased oxidised methionine (Ac-Met sulfoxide and Met sulfone) in *S. coelicolor* overexpressing *sco0954* (Fig. 7a, b), we tested whether a strong reducing agent (5 mM dithiothreitol, DTT) affects wall-deficient cell formation. DTT had no effect in the wild-type strain (with or without pRASK-PermE*) grown on GYM with 0.64 M sucrose, as wall-deficient cells were still not produced (Fig. 7c, d; see Supplementary Fig. S1 for representative images). However, DTT blocked S-cell formation in the strain overexpressing *sco0954* (Fig. 7e). This supports a role for SCO0954 in modulating PG oxidation, as reported for the *Salmonella enterica* MddA protein^57^, and suggests that redox changes in PG driven by *sco0954* overexpression influence wall-deficient cell formation. Notably, DTT does not suppress the formation of wall-deficient cells in the *sco4439/40::Tn5062* and *sco1760::Tn5* mutants (Fig. 7f, g).

Methionine oxidation and acetylation are post-translational modifications analogous to phosphorylation^62,63^, capable of altering the conformation of PG-associated proteins and, in turn, PG structure.

### The *sco1760::Tn5* and *sco4439/40::Tn5062* mutants, as well as the *S. coelicolor* wild-type strain overexpressing *sco0954,* exhibit altered peptidoglycan muropeptide composition

Peptidoglycan subunits, or muropeptides, differ between bacterial species^64,65^, and such variation affects PG architecture and cell shape^66^. We hypothesised that muropeptide composition might be altered in *sco1760::Tn5* and *sco4439/40::Tn5062* mutants, influencing PG properties and contributing to osmotic-stress-induced cell formation.

**Fig. 8:**
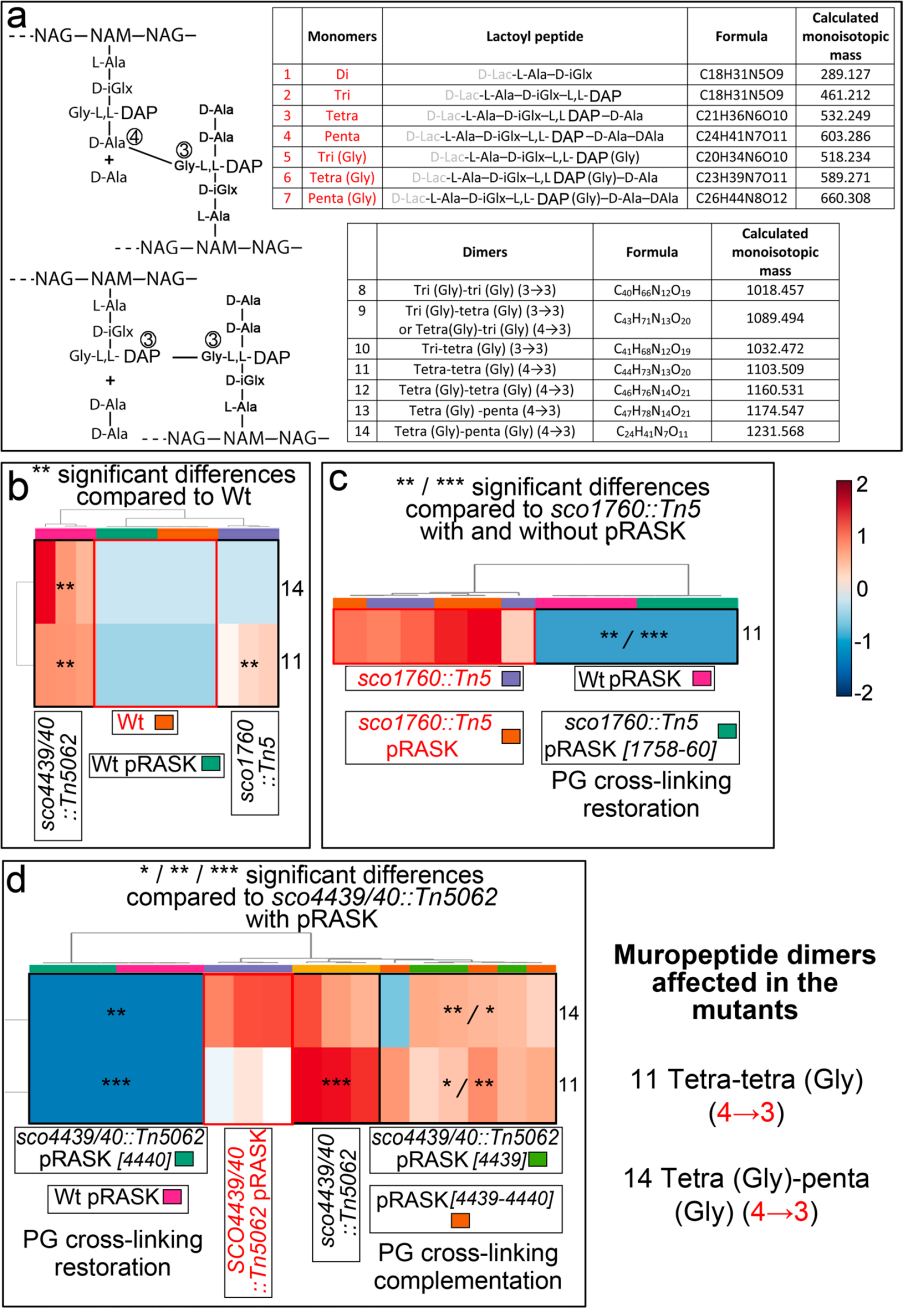
Muropeptide composition of peptidoglycan in the *sco4439/40::Tn5062* and *sco1760::Tn5* mutants, and in the corresponding complemented strains. **(a)** *S. coelicolor*, PG monomer and dimer (4−3 and 3–3 PG cross-linkages) muropeptide structures, with calculated monoisotopic masses. **(b)** Heatmap illustrating muropeptide abundance in the *sco4439/40::Tn5062* and *sco1760::Tn5* mutants, compared to the *S. coelicolor* wild-type strain with or without the empty pRASK plasmid. **(c)** Heatmap showing muropeptide abundance in the *sco1760::Tn5* mutant and its complemented strain, compared to the mutant harbouring pRASK. **(d)** Heatmap showing muropeptide abundance in the *sco4439/40::Tn5062* mutant and its complemented strains, compared to the mutant harbouring pRASK. The relative abundances of the muropeptides shown in the heatmaps (rows) exhibit statistically significant differences in at least one of the analysed strains compared to the corresponding reference strain (reference strains are highlighted in red). Asterisks indicate significant differences (*p* < 0.05, *p* < 0.01, *p < 0.001).

Hugonnet et al.^67^ previously characterised *Streptomyces coelicolor* PG muropeptides via LC-MS/MS (summarised in Fig. 8a). Here, we compared PG profiles of the wild-type (non-producing stress-induced cells) and mutants (producing stress-induced cells) (Figs. 8b–d), focusing on spores, as they derive from sporulating hyphae that are those involved in stress-induced cell production. While spores are easily isolated, sporulating hyphae are confined to the upper region of aerial hyphae and cannot be separated from non-sporulating forms^68,69^. We employed high-resolution mass spectrometry (ESI-Q-TOF Impact II HR-MS spectrometer) to identify muropeptides based on their exact monoisotopic masses, with a mass accuracy of ± 0.005 Da. Signal intensities were normalised to the tetra(Gly) monomer, the most abundant muropeptide in our PG analyses (see Methods for details).

Figure 8b shows altered muropeptide profiles in the *sco4439/40::Tn5062* and *sco1760::Tn5* mutants versus wild type (with or without pRASK). Both mutants exhibited increased tetra-tetra(Gly) (4–3) dimer (muropeptide 11); *sco4439/40::Tn5062* also showed elevated muropeptide 14. This aligns with SCO4439’s D-Ala-D-Ala carboxypeptidase activity^46^, whose absence leads to pentapeptide accumulation and subsequent (4–3) dimer formation. Figure 8c indicates that complementation with *sco1758–1760* restored muropeptide 11 to wild-type levels in *sco1760::Tn5*, matching phenotypic rescue (Fig. 4d). Figure 8d shows that *sco4440* complementation reduced muropeptides 11 and 14 to wild-type levels in *sco4439/40::Tn5062*, whereas *sco4439* alone or with *sco4440* had minimal effect.

Overall, these findings suggest that SCO4439, SCO4440, and SCO1758 influence the PG muropeptide composition of *S. coelicolor*. As discussed below, these PG alterations appear to contribute to the induction of wall-deficient cell formation.

## Discussion

As introduced above, non-replicative extracellular membrane vesicles and wall-deficient cells capable of division (L-forms) have been reported in many bacteria, including actinomycetes^17–19,26,70^, whereas wall-deficient cells unable to divide and induced by hyperosmotic stress (S-cells) have been specifically characterised in actinomycetes^26^. L-forms have been described as forming through a passive biophysical mechanism, dependent on an excess of intracellular membranes^36–38^. A similar passive mechanism for cell division has been proposed for the formation of EVs at the hyphal tips of *Streptomyces venezuelae*^20,21^. A biochemical modulation of EVs, S-cell, and L-form formation is compatible with this passive biophysical mechanism, as the intracellular membrane excess may be subject to genetic regulation. In this context, some genes (*murE*, *mraY*, *ftsQ*,* filP*,* stlP*) have already been reported to modulate L-form and S-cell formation in bacteria^23,37,42,43^. EV formation in gram-positive bacteria has been reported to be under genetic control (*sigB*, *covRS*)^35^. Our study advances this understanding by demonstrating that S-cell formation in *Streptomyces coelicolor* is triggered by the inactivation of *sco1758*, *sco4439*, and *sco4440*, as well as by the overexpression of *sco0954*. Moreover, the inactivation of *sco1758* induces both L-form and S-cell formation (Fig. 1). We also found that wall-deficient cells are only present in 48-hour cultures, which correspond to the aerial hyphae stage preceding sporulation, suggesting a connection between hyphal differentiation and the formation of wall-deficient cells. Hyperosmotic stress (0.64 M sucrose) trigger wall-deficient cell formation, whereas lower osmotic stress (0.3 M sucrose, sufficient to prevent osmotic lysis of wall-deficient cells) does not induce their formation (Fig. 1). Consequently, wall-deficient cell formation appears to be actively triggered under the 0.64 M sucrose condition, rather than sucrose merely protecting wall-deficient cells, produced under all conditions, from osmotic lysis. As *Streptomyces coelicolor* lacks invertase, it is unable to metabolise sucrose^71,72^. The formation of wall-deficient cells at high sucrose concentrations in the mutants is therefore likely driven by osmotic stress, rather than by a metabolic effect of sucrose, as previously reported in other actinomycetes such as the wild-type strains of *Kitasatospora viridifaciens*, *Streptomyces griseus* and *Streptomyces venezuelae*^26^, as well as in the *S. coelicolor* strain lacking the stomatin-like protein StlP^43^. In these actinomycetes, S cells are induced not only by sucrose, but also by other osmolytes such as NaCl and sorbitol^26,43^. Further studies will be required to determine whether osmolytes other than sucrose can similarly trigger S-cell formation in the *sco1760::Tn5* and *sco4439/40::Tn5062* mutants, as well as in *S. coelicolor* overexpressing *sco0954*.

The PG structure is altered in all the *S. coelicolor* mutants producing wall-deficient cells. Acetylated and oxidised methionine from PG-associated peptides is highly increased in the *S. coelicolor* strain overexpressing *sco0954* (Fig. 7a), whereas PG muropeptide composition is altered in the *sco4439/40::Tn5062* and *sco1760::Tn5* mutants (Fig. 8). PG muropeptide composition is fundamental in defining PG 3D structure, PG resistance, and bacterial cell shape. In fact, the shape of purified PG is reminiscent of the bacterial cell^73,74^. Methionine oxidation and acetylation also influence PG structure, as both post-translational modifications significantly affect protein conformation and function, in a manner similar to that of other PTMs such as amino acid phosphorylation^62,63^.

**Fig. 9:**
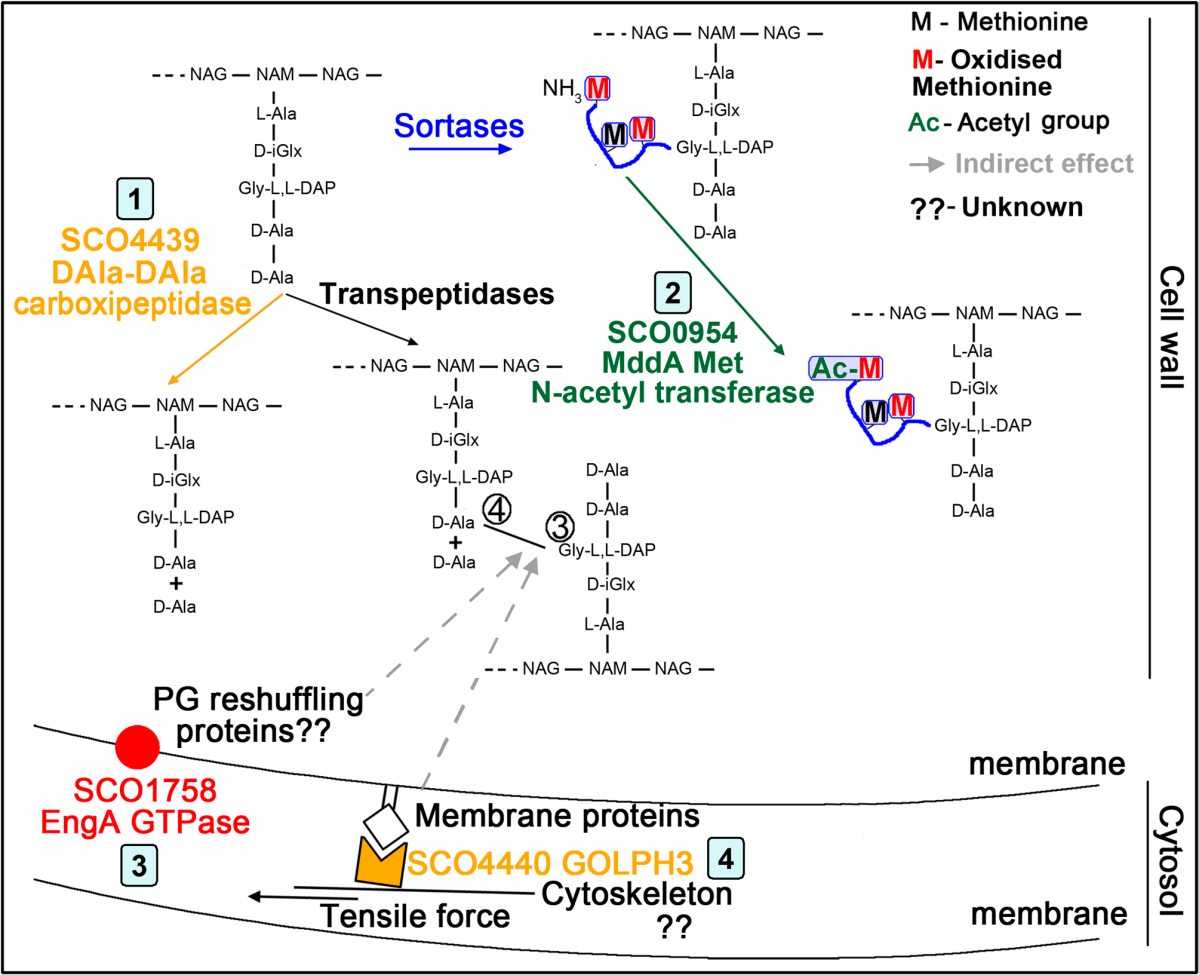
Model illustrating the roles of SCO4439, SCO4440, SCO0954, and SCO1758 in modulating peptidoglycan structure. The putative functions of SCO4439 (labelled as 1), SCO0954 (2), SCO1758 (3), and SCO4440 (4) in influencing PG muropeptide composition and the acetylation of oxidised methionine in PG-associated peptides are depicted. Solid arrows indicate direct modifications to the PG, while dashed arrows represent indirect effects on its structure. See Discussion for details.

We propose the model outlined in Fig. 9, describing the effect of SCO0954, SCO4439, SCO4440 and SCO1758 on PG structure and wall deficient cell formation under hyperosmotic sucrose conditions. First, *sco4439/40::Tn5062* mutant lack D-Ala-D-Ala carboxypeptidase activity^46^, leading to the accumulation of pentapeptides, which serve as substrates for increased formation of 4–3 cross-linked dimers (Fig. 8b). Second, *sco0954* overexpression increases acetylated oxidised methionine (Fig. 7a), consistent with SCO0954 acting as an N-acetyltransferase of oxidised methionine, similar to the prototype MddA N-acetyltransferase from *Salmonella enterica*^57^. Third, down-regulation of SCO1758 (EngA GTPase) in *sco1760::Tn5* (Fig. 4e) increases 4–3 dimers (Fig. 8b). Analogously to other GTPases involved in PG biosynthesis as the FtsZ GTPase^75^ or the CpgA GTPase^76^, SCO1758 might interact with specific PG reshuffling enzymes, positioning them in the hyphae destined to form wall-deficient cells. Fourth, SCO4440 also influences 4–3 muropeptide dimers, as their levels decrease when *sco4439/40::Tn5062* is complemented with *sco4440* (Fig. 8d). SCO4440 contains a GOLPH3-like domain, a feature of eukaryotic trans-Golgi proteins (Fig. 6c). While not previously reported in prokaryotes, GOLPH3-like proteins are widespread in actinomycetes (Supplementary Table S2), hinting at a role in a putative secretory network similar to the eukaryotic Golgi^59,77^. Like other cytoskeleton-interacting proteins involved in S/L-form (the *Kitasatospora viridifaciens* FilP cytoskeletal protein)^42^ and in the formation of intracellular membrane vesicles (the *S. coelicolor* SCO4256-4258 contractile cytosolic bacteriophage tail-like injection systems)^22^, SCO4440 might link membrane proteins with cytoskeleton elements.

In summary, SCO0954 (N-acetyltransferase), SCO4439 (carboxypeptidase), SCO4440 (GOLPH3-like), and SCO1758 (GTPase) contribute to wall-deficient cell formation under hyperosmotic sucrose stress (non-dividing S-cells) and SCO1758 is also involved in the formation of divisible L-forms. Alterations in muropeptide composition, particularly 4–3 dimers and PG-associated acetylated and oxidised methionine, modulate this process. *S. coelicolor* stress-induced wall-deficient cells (4–14 μm²; Fig. 3a) are up to four times larger than *Streptomyces* EVs (0.02–4.9 μm²)^17,21^, with both overlapping in the 4–4.9 μm² range, suggesting they are distinct structures that may nonetheless share mechanisms of formation and regulation. Additional research is needed to further elucidate how these proteins modulate PG architecture, determine whether homologous proteins induce wall-deficient cells in other bacteria, and to explore the potential of these genes to promote EV formation for biotechnological applications.

## Methods

### Bacterial strains and culture conditions

All *Streptomyces* and *Escherichia coli* strains used are listed in Supplementary Table S3. Spores were harvested from SFM solid plates^47^ after incubation at 30 °C for 7 days. Osmotic-stress-induced cell formation was assessed on GYM^78^ (5 g/L glucose, 4 g/L yeast extract, 5 g/L malt extract, 0.5 g/L MgSO₄·7 H₂O, 20 g/L agar; post-autoclaving, supplemented with sterile 0.5 g/L K₂HPO₄). Twenty-five millilitres of medium were dispensed into 90-mm, 3-vent Petri dishes. Plates were overlaid with cellophane, inoculated with 3 × 10⁷ fresh (non-frozen) spores, and incubated at 30 °C. Hyperosmotic stress to induce wall-deficient S-cell formation was created by the addition of 0.64 M sucrose, as described by Ramijan et al.^26^.

*E. coli* strains were grown in LB or 2×TY at 37 °C. Antibiotics for plasmid and mutant selection included: ampicillin (100 µg/mL), apramycin (100 µg/mL for *E. coli*, 25 µg/mL for *S. coelicolor*), hygromycin (100 µg/mL for *E. coli*, 200 µg/mL for *S. coelicolor*), kanamycin (50 µg/mL), thiostrepton (50 µg/mL), chloramphenicol (25 µg/mL), and nalidixic acid (25 µg/mL).

### Random mutagenesis

The *S. coelicolor sco1760* ORF, disrupted by mini-Tn5 (sco1760::Tn5), was identified from a random mutagenesis library generated in our laboratory using the method of Xu et al.^45^.

### Directed mutagenesis

The *sco4439* ORF was disrupted in a previous study^46^ using the Tn5062-based single-gene knockout library developed by Fernández-Martínez et al.^79^. As shown by Rioseras et al.^46^, the transposon insertion caused a deletion encompassing the promoter regions of sco4439 and sco4440, thereby abolishing expression of both genes.

### Complementation of the *sco4439/40::Tn5062,* and the *sco1760::Tn5* mutants

The *sco4440* was previously cloned into pMS82 by Rioseras et al.^46^. In this study, *sco4439* was excised from pTOPO [*sco4439*]^46^ using *EcoRV* and *SpeI*, and cloned into similarly digested pRASK^50^, yielding pRASK [*sco4439*].

*sco4440* and the *sco4440-41-42* fragment were excised from their respective pTOPO constructs^46^ using the *EcoR*V and *Spe*I and cloned into pRASK, generating pRASK [*sco4440*] and pRASK [*sco4440-41-42*], respectively.

To construct pRASK [*sco4439-4440*], a PCR product from wild-type genomic DNA was amplified using primers *sco4439/4440-F* and *sco4439/4440-R*, cloned into pCR™-Blunt II-TOPO (Thermo Fisher), sequenced, and subcloned into pRASK via *SpeI* and *EcoRV*.

*sco1760*, *sco1760-1759*, and *sco1760-1758* (including its upstream P1 promoter region) were amplified using primer pairs *sco1760F/sco1760R*, *sco1760F/SCO1759R*, and *sco1760F/sco1758R*, respectively. Products were cloned into pCR™-Blunt II-TOPO (Thermo Fisher), sequenced, and subcloned into pRASK^50^.

The pRASK plasmids containing the genes used for complementation were introduced into the mutants via conjugation^47^. Integration of ɸC31 was confirmed using the primers *sco3798intF* and *intR*^80^.

### Protein sequence analyses

Orthologous amino acid sequences of *S. lividans*, *S. avermitilis*, *S. venezuelae*, *S. scabies*, *S. griseus*, and *S. clavuligerus* for SCO0954, SCO4439, SCO4440, and SCO1758 were retrieved from StrepDB (http://strepdb.streptomyces.org.uk/). Conserved domains were identified using the Conserved Domain Database (CDD) (https://www.ncbi.nlm.nih.gov/Structure/cdd/cdd.shtml). Pairwise amino acid similarities were calculated with EMBOSS Needle (https://www.ebi.ac.uk/Tools/psa/emboss_needle/). Sequence alignments were generated using MUSCLE via the Phylemon platform (http://phylemon.bioinfo.cipf.es/)^81^.

### DNA and RNA extraction

Genomic DNA was isolated using standard methods^47^. RNA was extracted with Direct-zol™ RNA columns (Zymo-Spin™), treated with TURBO™ DNase (Thermo Fisher) and assessed using a Nanodrop 2000 (Thermo Fisher) and a 2100 Bioanalyzer (Agilent).

### *FtsZ* mutant transcriptomic analyses

Next-generation sequencing (NGS) was performed by Stab Vida (Caparica, Portugal) using two biological replicates of the *S. coelicolor* FtsZ mutant^14^ and the M145 wild-type strain. Ribosomal RNA was depleted with the Ribo-Zero Bacteria Kit (Illumina), and cDNA libraries were prepared using the TruSeq Stranded mRNA Library Preparation Kit (Illumina). Sequencing was conducted on the Illumina HiSeq 2500 platform with 100-bp paired-end reads. Bioinformatic analyses were performed on Linux using FastQC (quality control), Cutadapt (trimming), Bowtie2 (mapping to the *S. coelicolor* genome), and Cuffdiff (differential expression analysis)^82^.

### Overexpression of *sco0954*

The *sco0954* gene was amplified from *S. coelicolor* using primers *sco0954F/R* (Table S3), which included *Nde*I and *Spe*I. The amplicons were cloned into pCR™-Blunt II-TOPO and sequenced by Sanger using M13F/R. The ORFs were excised from pCR™-Blunt II-TOPO with *Nde*I and *Spe*I and subcloned into pNG4^83^ using the same enzymes. *PermE-sco0954* was excised from pNG4 with *Mfe*I and *Bgl*II and cloned into pRASK^50^ (pRASK-*PermE*[*sco0954*]). The empty pRASK-PermE* plasmid was constructed by digesting pRASK-PermE*[sco0954] with *Nde*I and *Hpa*I, blunting with S1 nuclease (Thermo Fisher™) and religating.

### Real-time quantitative reverse-transcription PCR (qRT-PCR)

cDNA was synthesised from 1.5 µg of RNA from two biological replicates using the High-Capacity cDNA Reverse Transcription Kit (Applied Biosystems, Waltham, MA, USA). Real-time PCR was performed on an FQD-96 A fluorescence detection system (BIOER, China) in triplicate reactions containing 2 µL cDNA, 10 µL PowerTrack™ SYBR Green Master Mix (Thermo Fisher), and 300 nM of gene-specific primers (Table S3) in a final volume of 20 µL. *sco1758* was amplified with primers q1758F2/R2 (Table S3). Negative controls (RNA or water in place of cDNA) were used to assess DNA contamination and primer-dimer formation. Amplification conditions: 2 min at 50 °C, 10 min at 95 °C, then 40 cycles of 15 s at 95 °C and 1 min at 60 °C.

Absolute quantification was performed using standard curves generated from PCR-amplified *sco1758* fragments^84^, all with correlation coefficients > 0.99. Transcript abundance was normalised by amplicon size and molecular weight, and expressed as transcript copies per microgram of cDNA. Three biological replicates were analysed per gene. Statistical significance was assessed by two-sided t-tests; p-values < 0.05, < 0.01, or < 0.001 were considered significant.

### Cell morphology, viability, membrane, cell-wall staining, time-lapse analyses

Cell morphology and viability were assessed using PI and SYTO 9 stains from the LIVE/DEAD BacLight Bacterial Viability Kit (Invitrogen). Stains were prepared in 103 g/L sucrose to prevent osmotic lysis. Samples were imaged using a Leica TCS-SP8 confocal laser-scanning microscope (excitation: 488 nm and 568 nm; emission: 530 nm [green] and 640 nm [red])^85^.

Cell walls were stained with Alexa Fluor 488-conjugated WGA (Invitrogen), which binds N-acetylglucosamine and N-acetylmuramic acid. *Streptomyces* hyphae were scraped from cellophane with a spatula and processed as described^15^. Samples were imaged at excitation/emission wavelengths of 498 nm/520 nm.

Time-lapse imaging was performed using FM5-95 (3.95 µg/mL) and SYTO9 (0.5 µmol/L) in solid cultures. Cultures were grown on cellophane, pre-incubated at 30 °C for 48 h, and transferred to fresh medium containing the stains. The sheets were inverted onto coated µ-dishes (Ibidi GmbH) and incubated at 30 °C. Imaging was performed every 13 min over 17 h on a Leica TCS-SP8 confocal microscope (excitation: 488 nm and 522 nm; emission: 530 nm [SYTO9] and 782 nm [FM5-95]). The microscope chamber was equilibrated for 3 h prior to imaging. Unstained cultures served as autofluorescence controls. Images were processed using Fiji software^48^ to adjust histograms, add scales, and assemble time-lapse videos.

### Quantification of osmotic-stress-induced EVs size and abundance

Cell size and abundance under osmotic stress were quantified from confocal images stained with PI and SYTO 9, or SYTO9 plus FM5-95. Vesicle size was measured using a Fiji macro^48^ with the Stardist plugin^86^, which extracted parameters including area, perimeter, circularity, Feret diameter, minimum Feret diameter, aspect ratio (AR), roundness, and solidity. A circularity threshold of 0.8 was applied (1.0 = perfect sphere). The vesicle area threshold was defined as 3.6 μm² based on control images of the wild-type strain (with or without empty plasmids). Over 100 vesicles exceeding this threshold were analysed from images taken from at least three biological replicates.

Vesicle abundance was calculated as the percentage of vesicle-stained area relative to total stained area per image. Vesicle area was quantified as above, and total stained area was measured by counting pixels above background and converting to µm². Background levels were estimated from hyphae-free regions. At least 100 vesicles were analysed from three biological replicates.

Statistical differences were assessed using a two-sided t-test, with p-values of < 0.05, < 0.01, or < 0.001 considered significant.

### Quantification of membrane and cell wall abundance

The membrane-to-cell wall ratio was determined from FM5-95- and WGA-stained confocal images by quantifying pixels above background using Fiji^48^. Background levels for each stain were calculated per image from hyphae-free regions. For each strain, data from at least three biological replicates were combined, with a minimum of 1 × 10⁶ pixels above background analysed per replicate. The FM5-95/WGA pixel ratio reflects membrane abundance: a ratio of 1 indicates equal membrane and wall content; <1 indicates more wall than membrane; and > 1 indicates more membrane than wall. Statistical significance was assessed using a two-sided t-test, with p-values < 0.05, < 0.01, or < 0.001 considered significant.

### Muropeptide abundance quantification

Peptidoglycan was purified from *Streptomyces* spores as described previously^46,87^, resuspended in 3 mL mQ and stored at − 80 °C. *Streptomyces* muropeptides^67^ were quantified by high-resolution LC-MS. PG (100 µL) was digested with 50 U/mL mutanolysin (A&A Biotechnology) and 2 mg/mL lysozyme in 25 mM phosphate buffer (pH 7) with 0.1 M MgCl₂. Lactoyl peptides were released using 32% ammonium hydroxide^88^, dried, and resuspended in 50 µL mQ water with 0.1% formic acid. Five µL were analysed by LC-HRESI-MS using a Dionex Ultimate 3000 UHPLC system and Bruker Impact II Q-TOF in positive mode (m/z 50–2500). Chromatographic separation used a Zorbax^®^ Eclipse Plus C18 column (50 × 2.1 mm, 1.8 μm) at 0.25 mL/min with a formic acid/acetonitrile gradient.

Data were processed with DataAnalysis v4.3, calibrated with internal standards. Muropeptides were identified using a ± 0.005 Da mass and ± 0.1 min RT tolerance. [M + H]⁺ was dominant in monomers, [M + 2 H]²⁺ in dimers. Relative abundances (peak areas) were normalised to the most abundant Tetra (Gly) monomer. Statistical analyses were performed in MetaboAnalyst 6.0^89^, with missing values imputed as 1/5 of the minimum, log₁₀-transformed, and Pareto scaled. Comparisons included wild-type and mutant strains with or without plasmids. Triplicate samples were analysed. Significance thresholds were *p* < 0.05, < 0.01, or < 0.001.

### Quantification of N-acetylmethionine, N-acetylmethionine sulfone, and N-acetylmethionine sulfoxide

One hundred µL of purified PG were digested overnight at 37 °C with 50 U/mL mutanolysin (A&A Biotechnology) and 2 mg/mL lysozyme (GoldBio) in 25 mM phosphate buffer (pH 7) containing 0.1 M MgCl₂. The reaction was stopped by boiling for 3 min. After centrifugation (16,000 × g), the supernatant was digested with 0.2 mg/mL pronase (MedChem Express) at 37 °C for 20 h. Proteins were precipitated with 5% TCA on ice for 30 min, followed by centrifugation and vacuum-drying of the supernatant. The pellet was resuspended in 100 µL ultrapure water, vortexed, and diluted with 900 µL acetonitrile (ACN). An aliquot (25 µL) was injected into an Agilent 6410 LC-MS/MS system (ESI-QqQ) operated in MRM mode. Parameters included a 35 psi nebulizer, + 4000 V capillary voltage, and nitrogen gas at 12 L/min and 350 °C.

Chromatography was performed using a XBridge^®^ Amide column (Waters) (4.6 × 150 mm, 3.5 μm; Waters) at 1 mL/min with a gradient of Solvent A (0.1% formic acid in water) and Solvent B (ACN): 85–80% B (0–5 min), 80–50% B (5–15 min), and re-equilibrated at 85% B for 4 min. Standards included N-acetyl-L-methionine, its sulfoxide and sulfone forms (AOZEAL and Sigma-Aldrich), as well as non-acetylated forms (Sigma-Aldrich). Instrument parameters are listed in Supplementary Table S4. Three biological replicates per sample were analysed; statistical significance was set at *p* < 0.05, < 0.01, or < 0.001.

## Supplementary Information

Below is the link to the electronic supplementary material.

Supplementary Table S1

Supplementary Table S2

Supplementary Table S3

Supplementary Table S4

Supplementary Fig. S1

Supplementary Fig. S2

Supplementary Material 7

Supplementary Material 8

Supplementary Material 9

Supplementary Movies S1-S3.

## Data Availability

The authors declare that the data supporting the findings of this study are available within the article and its supplementary information files or from the corresponding authors on request. The RNAseq raw data are available on the SRA database (https://www.ncbi.nlm.nih.gov/sra) under the PRJNA1024652 accession number.
