# Supplementary material for: Peptidoglycan-reshuffling proteins SCO0954, SCO1758, SCO4439, and SCO4440 modulate the formation of wall-deficient cells in Streptomyces coelicolor under hyperosmotic sucrose stress
Source: Sci Rep. 2025 Sep 1;15:32112. doi: 10.1038/s41598-025-15457-z (PMC12402492; doi:10.1038/s41598-025-15457-z)
Supplement: Supplementary file 3 — Supplementary Table S3 [file 41598_2025_15457_MOESM3_ESM.pdf]

Supplementary Table S3. Bacterial strains, plasmids and primers used in this study.

Strain	Description	Reference
S. coelicolor M145	SCP1⁻ SCP2⁻ , reference strain	[1]
S. coelicolor sco4439::Tn5062	*S. coelicolor* interrupted at the *SCO4439* ORF, AmR	[2]
S. coelicolor sco1760::Tn5	*S. coelicolor* interrupted at the *SCO1760* ORF, ApraR	This study
E. coli TOP10	F- *mcr*A Δ (*mrr-hsd*RMS-*mcr*BC) φ80*lac*ZΔM15 Δ*lac*X74 *rec*A1 *ara*D139 Δ (*ara-leu*)7697 *gal*U *gal*K *rps*L *end*A1 *nup*G	Invitrogen
E. coli ET12567	*dam-13::*Tn9*, dcm-6, hsdM*, *hsdR*	[3]
E. coli ET12567/pUZ8002	*E. coli* ET12567 harbouring pUZ8002, a non-self-transmissible plasmid which can mobilize *oriT*-containing plasmids by conjugation	[4]

Plasmids	Description	Reference
PCR™-Blunt II-TOPO®	Zero Blunt® TOPO® PCR Cloning Kit, Kan^R	Invitrogen
pMS82	Integrative and conjugative vector, $Hygro^R$	[5]
pRASK	pRAS[6] harbouring kanamycin resistance	[6,7]
pRASK [*sco4439*]	pRASK harbouring *SCO4439*	This study
pRASK [*sco4440*]	pRASK harbouring *SCO4440*	This study
pRASK [*sco4440-41-42*]	pRASK harbouring *SCO4440*, *SCO4441* and *SCO4442*	This study
pRASK-PermE*	pRASK harobouring the PermE* promoter	This study
pMS82 [*sco4440*]	pMS82 harbouring *SCO4440*	[8]
pMS82 [*sco4439*]	pMS82 harbouring *SCO4439*	[8]
pNG4	Integrative and conjugative vector harbouring *PermE** promoter, $Hygro^R$	[8]

Primer	Sequence	Reference
M13F	CAGGAAACAGCTATGA	Invitrogen
M13R	CTGGCCGTCGTTTTAC	Invitrogen
sco1760F	AAAAACTAGT TGGCACGCACGCTCGCCG	This study
sco1760R	GGGGGATATCTCACTTCCCGGCCCGCTTCT	This study
sco1759R	GGGGGATATCTCAGCGCCCGGTGAGGCGC	This study
sco1758R	GGGGGATATCCGCTTGCCTGTTCCCGCTTCC	This study
sco0954F	GGGGCATATGCTGATCAGGGAAGCCAC	This study
sco0954R	AAAAACTAGTGACTGAGCCGGGACCTACA	This study
q1758F2	ATGAACGACCACATCCACCC	This study
q1758R2	CTCGATGTCGAAGCCCTCTT	This study

References

1 Kieser, T. *Practical Streptomyces genetics*. (John Innes Foundation, 2000).

2 Rioseras, B. *et al.* Characterization of SCO4439, a D-alanyl-D-alanine carboxypeptidase involved in spore cell wall maturation, resistance, and germination in *Streptomyces coelicolor Sci Rep* **6**, 21659, doi:10.1038/srep21659 (2016).

3 MacNeil, D. J. *et al.* Analysis of *Streptomyces* avermitilis genes required for avermectin biosynthesis utilizing a novel integration vector. *Gene* **111**, 61-68 (1992).

4 Flett, F., Mersinias, V. & Smith, C. P. High efficiency intergeneric conjugal transfer of plasmid DNA from Escherichia coli to methyl DNA-restricting streptomycetes. *FEMS Microbiol Lett* **155**, 223-229 (1997).
5 Gregory, M. A., Till, R. & Smith, M. C. Integration site for *Streptomyces* phage phiBT1 and development of site-specific integrating vectors. *J Bacteriol* **185**, 5320-5323 (2003).
6 Perez-Redondo, R., Santamarta, I., Bovenberg, R., Martin, J. F. & Liras, P. The enigmatic lack of glucose utilization in *Streptomyces clavuligerus* is due to inefficient expression of the glucose permease gene. *Microbiology (Reading)* **156**, 1527-1537, doi:10.1099/mic.0.035840-0 (2010).
7 Fernández-García, G. *et al.* The DeoR-like pleiotropic regulator SCO1897 controls specialised metabolism, sporulation, spore germination, and phosphorus accumulation in Streptomyces coelicolor. *Communications Biology* **7**, 1457 (2024).
8 Gonzalez-Quinonez, N. *et al.* New PhiBT1 site-specific integrative vectors with neutral phenotype in *Streptomyces*. *Appl Microbiol Biotechnol* **100**, 2797-2808, doi:10.1007/s00253-015-7271-0 (2016).
