# Supplementary material for: Peptidoglycan-reshuffling proteins SCO0954, SCO1758, SCO4439, and SCO4440 modulate the formation of wall-deficient cells in Streptomyces coelicolor under hyperosmotic sucrose stress
Source: Sci Rep. 2025 Sep 1;15:32112. doi: 10.1038/s41598-025-15457-z (PMC12402492; doi:10.1038/s41598-025-15457-z)
Supplement: Supplementary file 4 — Supplementary Table S4 [file 41598_2025_15457_MOESM4_ESM.pdf]

Supplementary Table S4. Experimental parameters for the LC-MS/MS analysis of N-acetylmethionine, N-acetylmethionine sulfone, and N-acetylmethionine sulfoxide.

Compound	Mw (g/mol)	Retention time (min)	Precursor ion (m/z)	Product ion (m/z)[a]	Fragmentor (V)	Collision energy (eV)
N-AcetyL-L-Methionine	191.25	2.0	192.0	144.0/104.0	72	5/13
AcetyL Methionine Sulfoxide	207.24	3.7	208.0	98.0/144.0	72	20/5
AcetyL Methionine Sulfone	223.24	2.3	224.0	56.0/182.0	72	20/5

[a] In bold, product ion (m/z) used for quantification.
