# Supplementary material for: Peptidoglycan-reshuffling proteins SCO0954, SCO1758, SCO4439, and SCO4440 modulate the formation of wall-deficient cells in Streptomyces coelicolor under hyperosmotic sucrose stress
Source: Sci Rep. 2025 Sep 1;15:32112. doi: 10.1038/s41598-025-15457-z (PMC12402492; doi:10.1038/s41598-025-15457-z)

Supplementary Fig. S1. Representative confocal microscopy images of EVs used for area quantification and for calculating membrane/cell wall ratios. (a) Images of EVs used to quantify EVs areas and to generate the histograms shown in Fig. 1. Quantified EVs are indicated. (b) Images of EVs used to generate the area histograms shown in Fig. 4. Quantified EVs are indicated. (c) Images used to calculate the membrane/cell wall ratio, as presented in the histogram in Fig. 2h.

(a) Representative images of EVs used to quantify EV areas and to generate the histograms shown in Fig. 1.

Fig. 1a Wt growing on GYM 0,64M sucrose 48-hour culture. SYTO9 (green) and PI (red) staining.

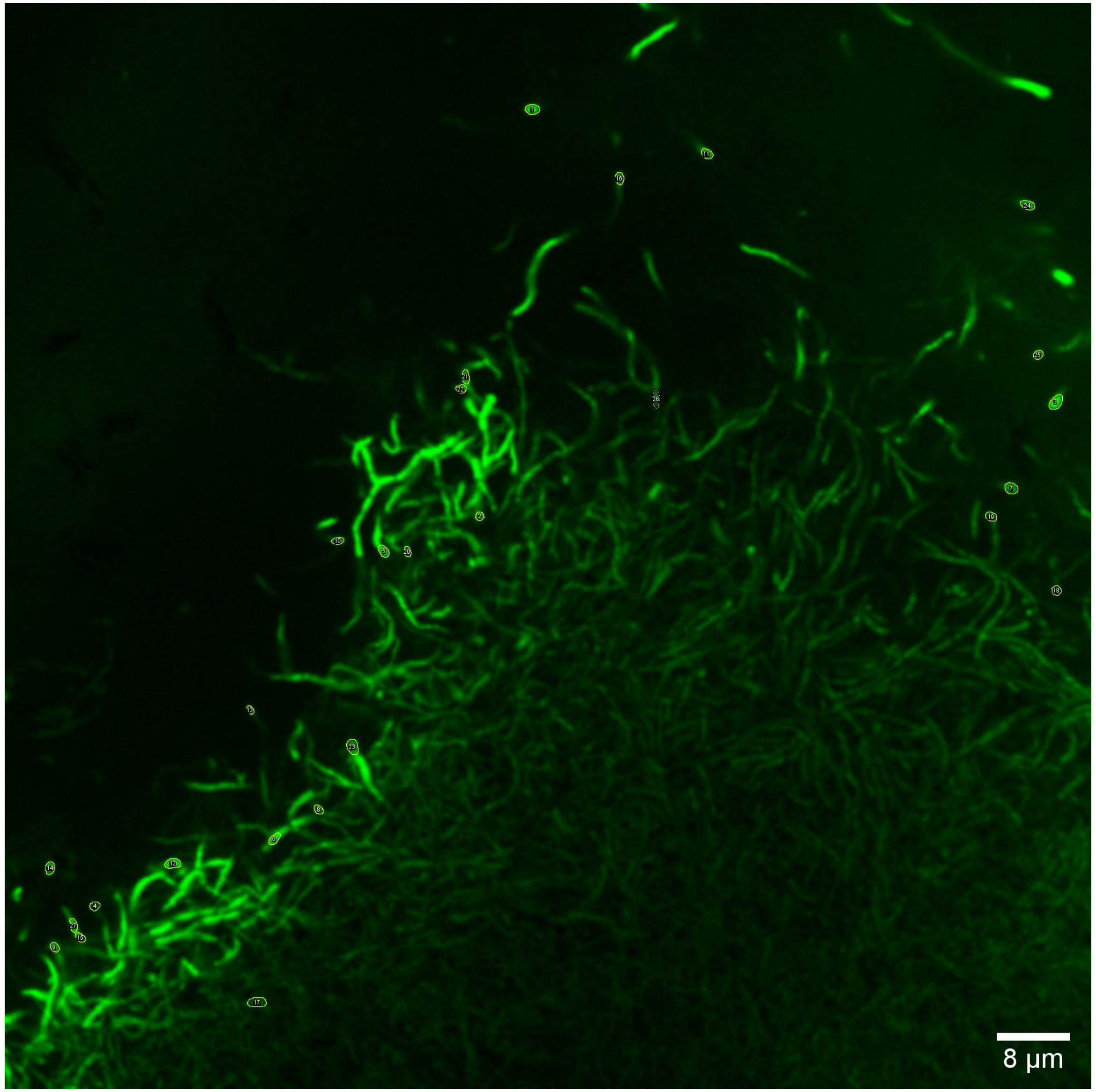

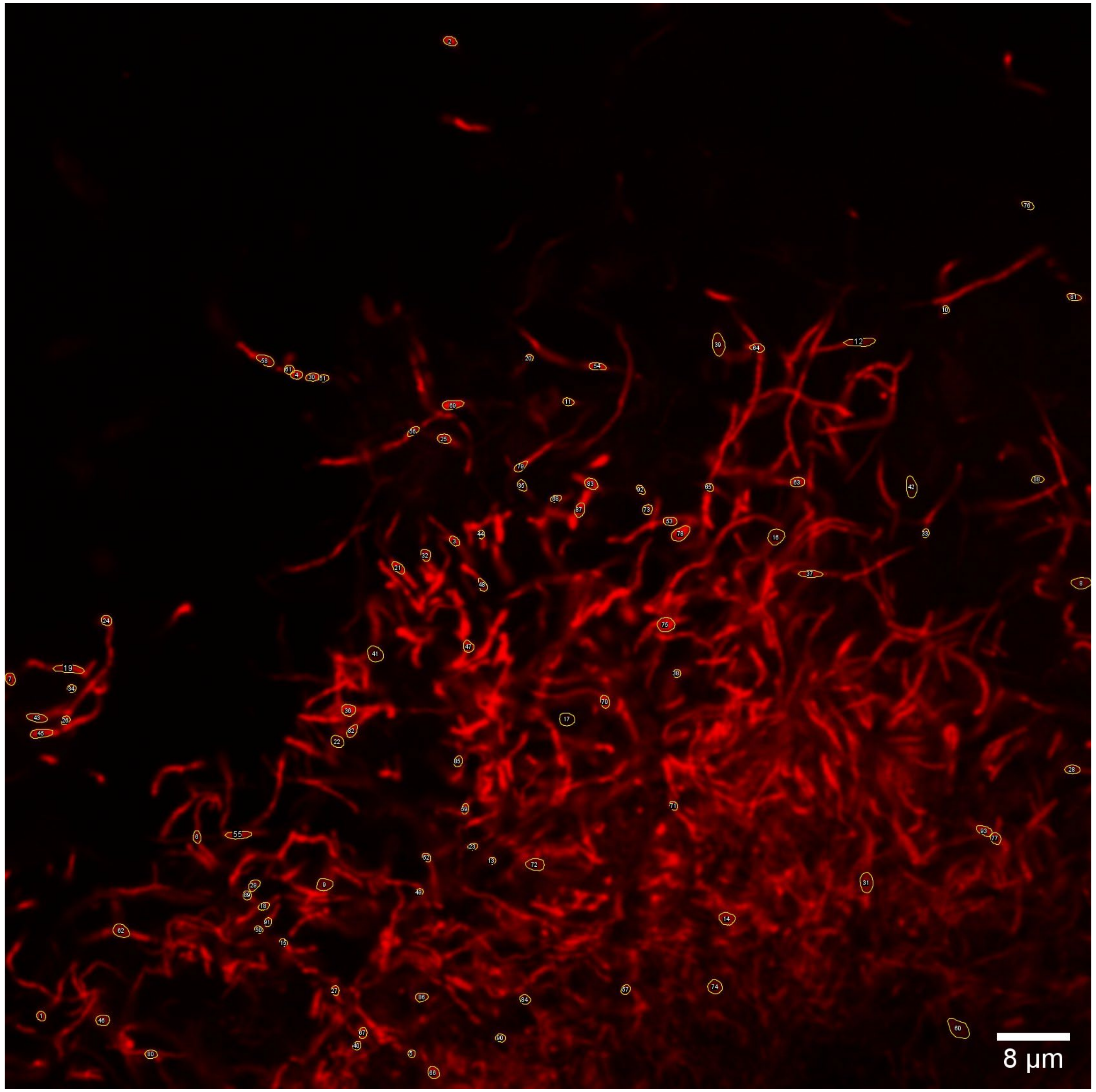

(a) Representative images of EVs used to quantify EV areas and to generate the histograms shown in Fig. 1.

Fig. 1b *sco1760::Tn5* growing on GYM 0,64M sucrose 48-hour culture. SYTO9 (green) and PI (red) staining.

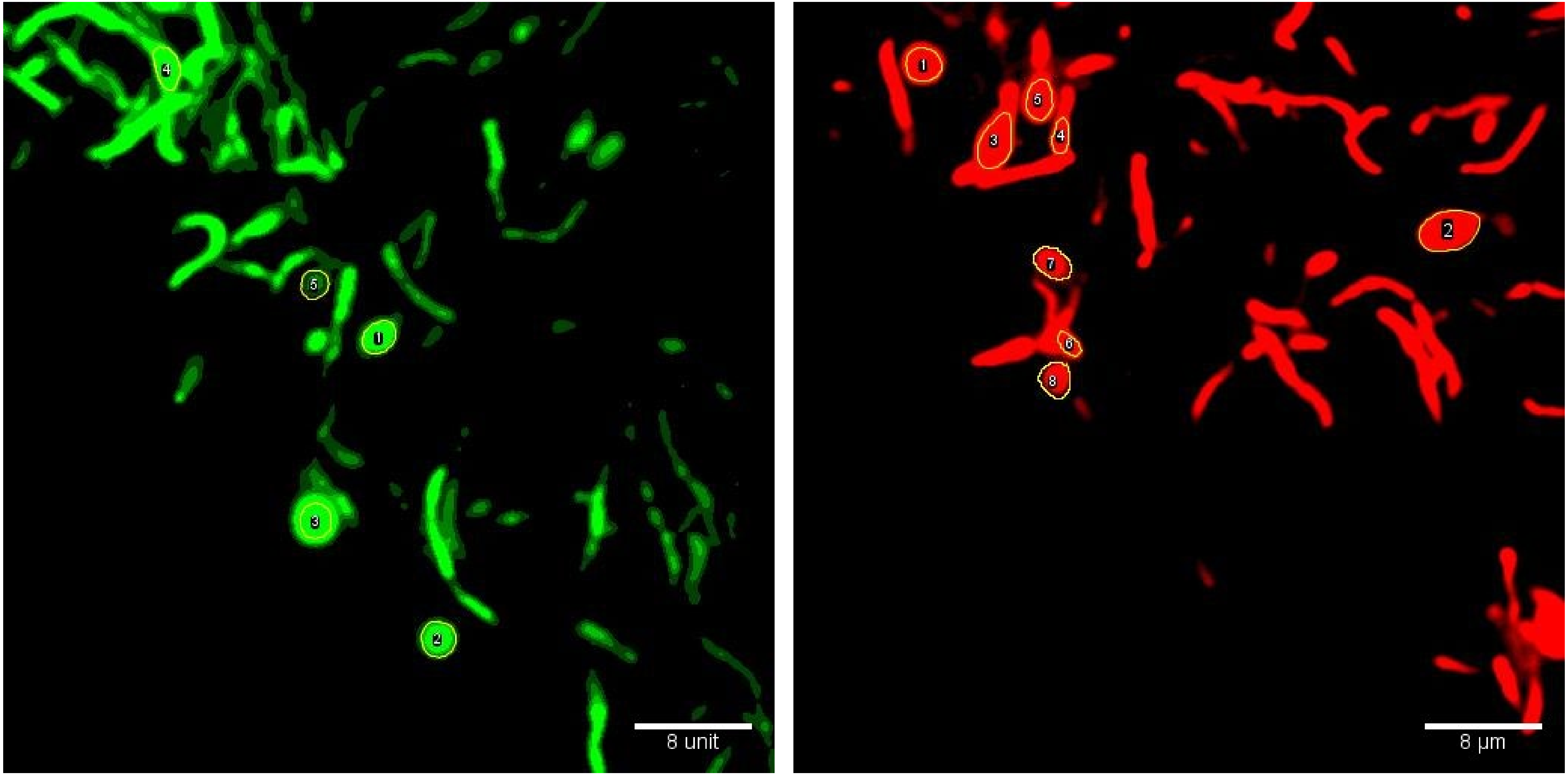

(a) Representative images of EVs used to quantify EV areas and to generate the histograms shown in Fig. 1.

Fig. 1c *sco4439/40::Tn5062* growing on GYM 0,64M sucrose 48-hour culture.

SYTO9 (green) and FM5-95 (red) staining.

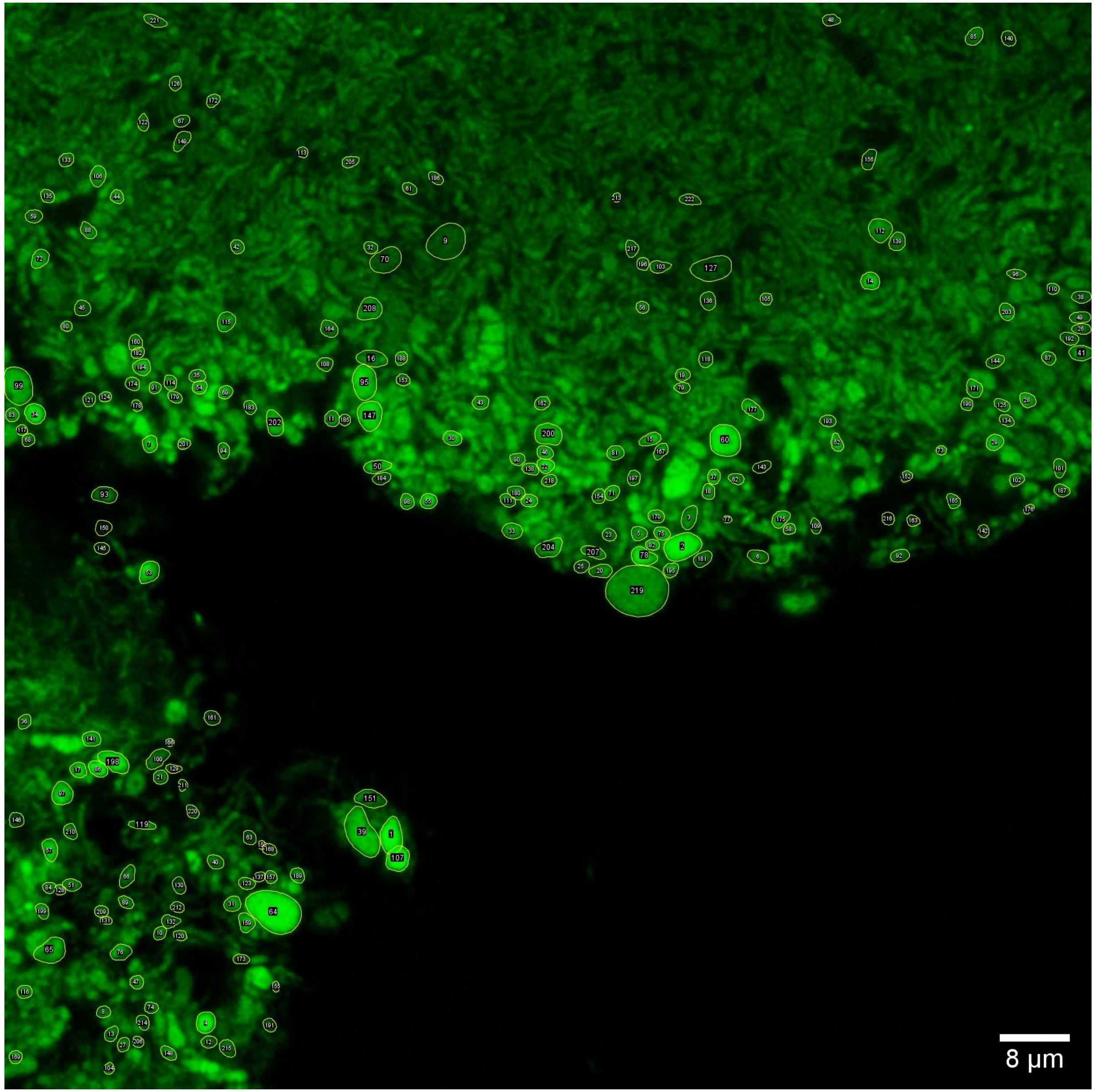

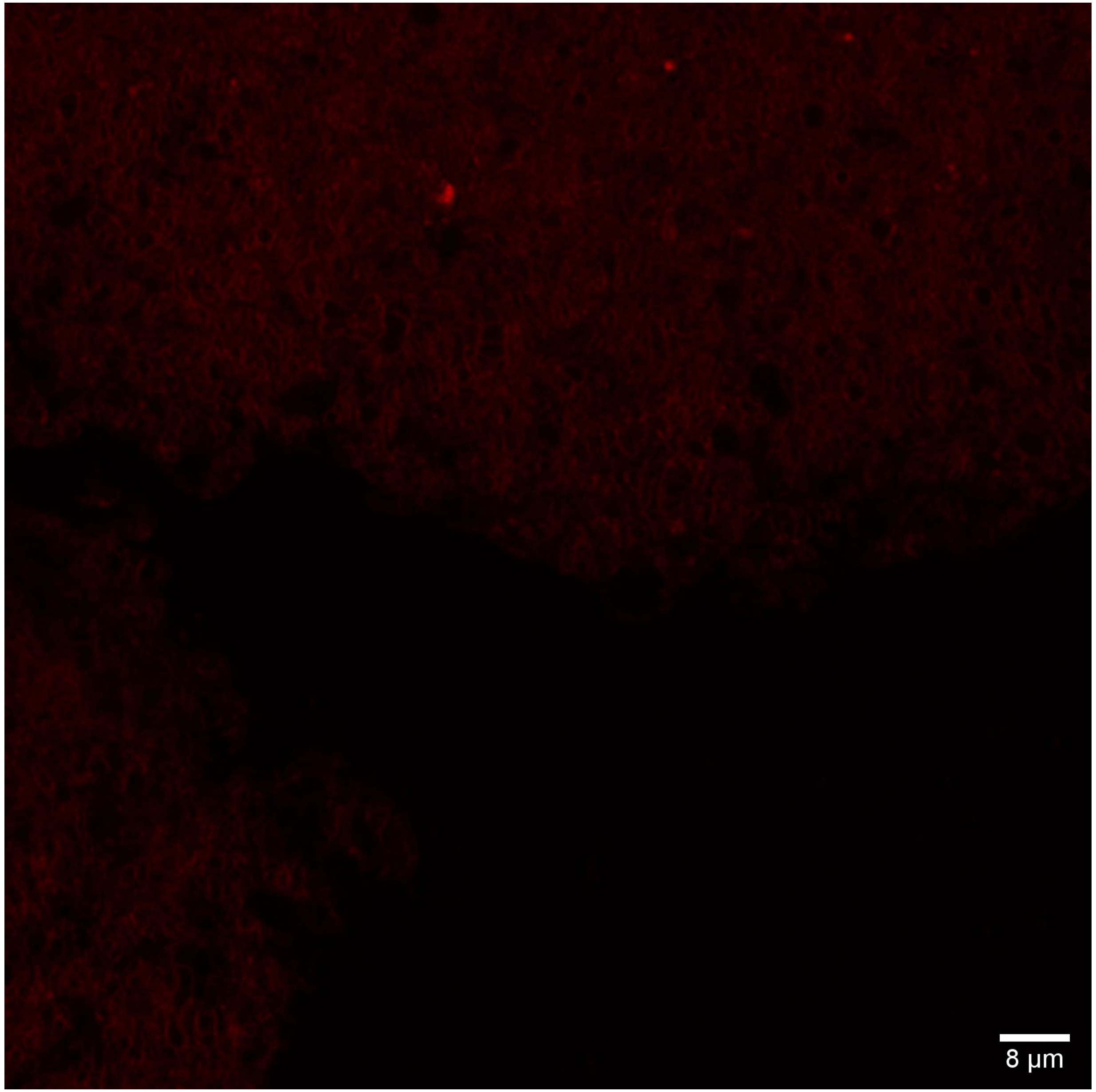

(a) Representative images of EVs used to quantify EV areas and to generate the histograms shown in Fig. 1.

Fig. 1d *S. coelicolor* overexpressing *sco0954* growing on GYM 0,64M sucrose 48-hour culture. SYTO9 (green) and FM5-95 (red) staining.

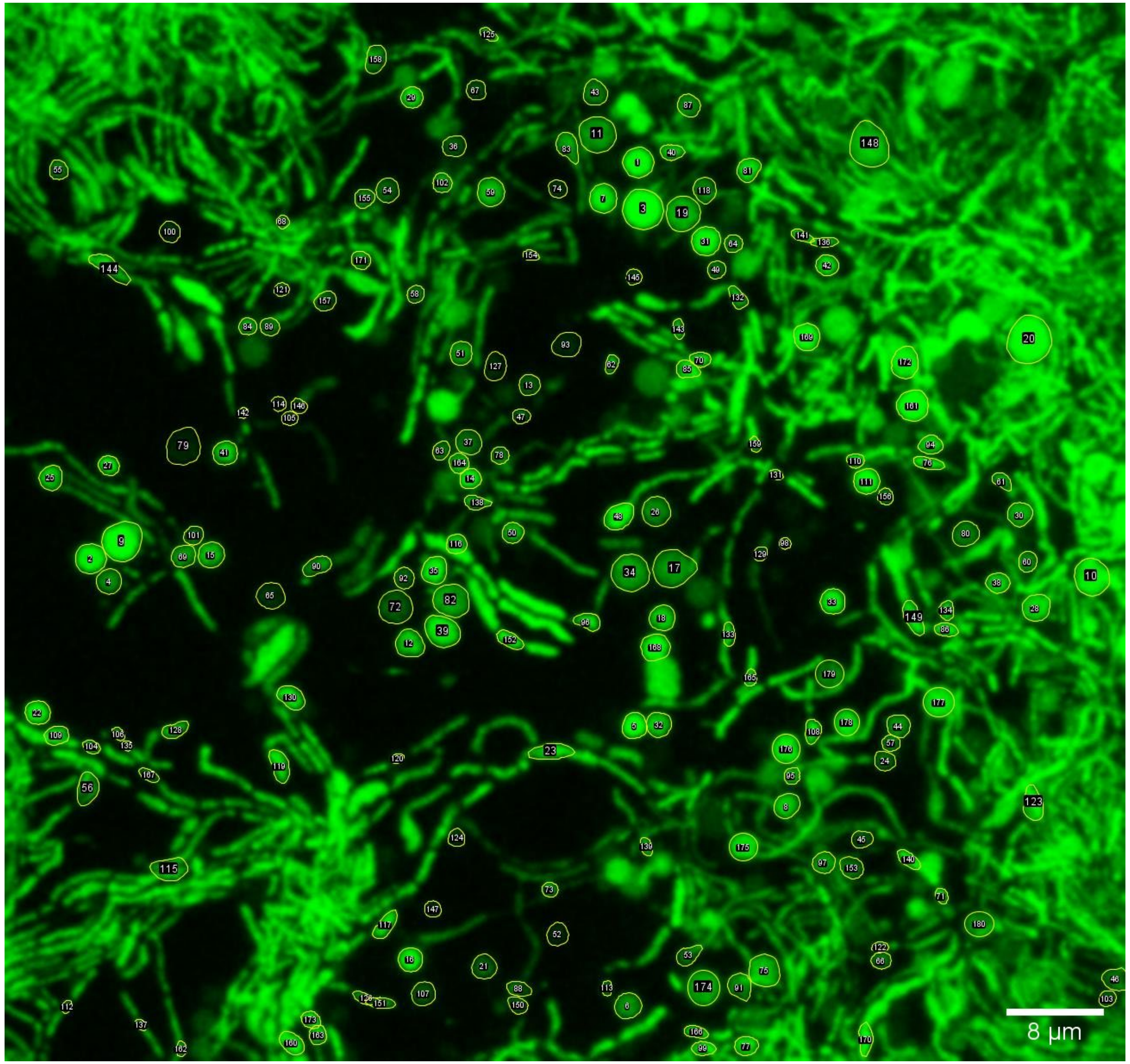

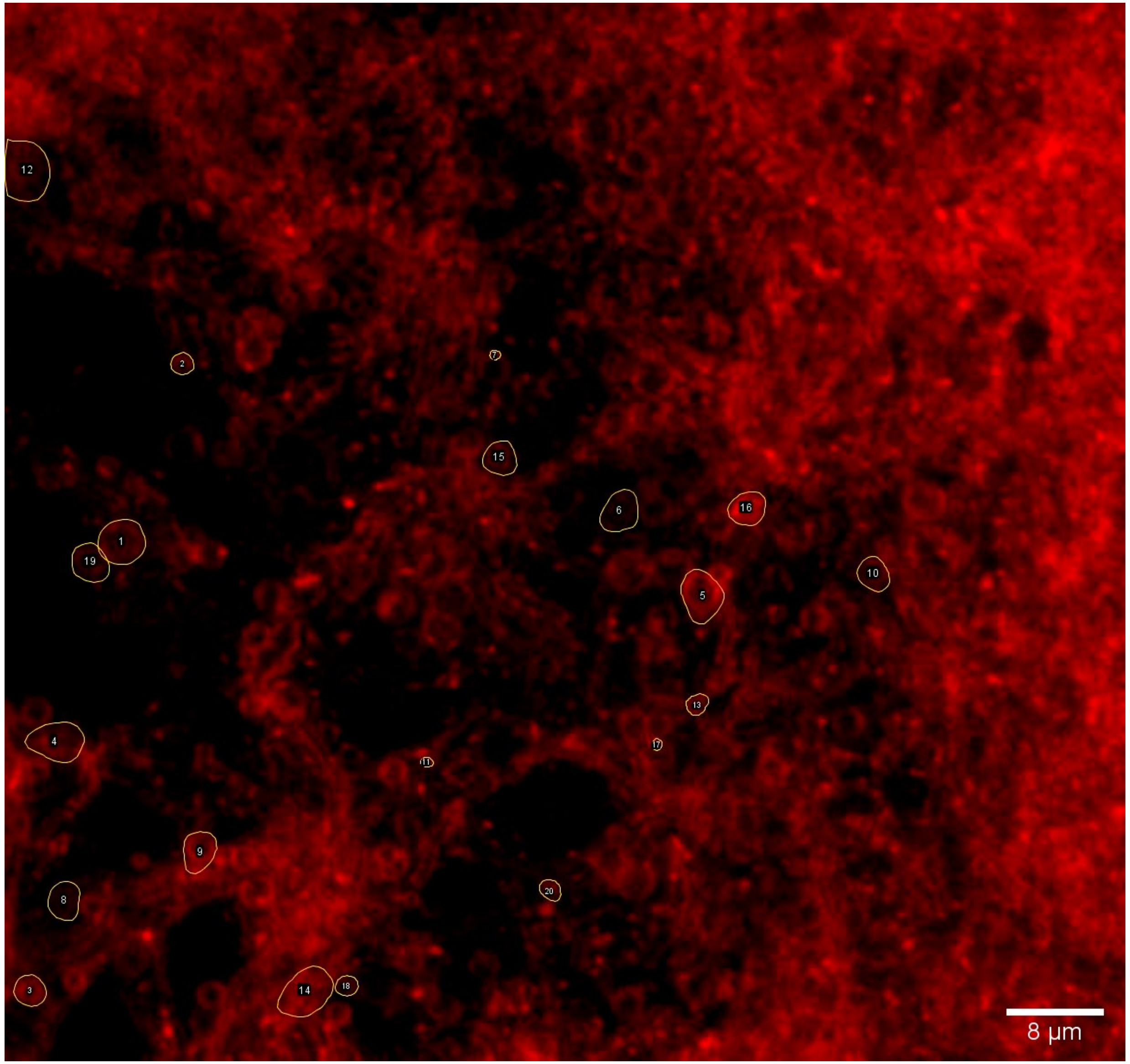

(a) Representative images of EVs used to quantify EV areas and to generate the histograms shown in Fig. 1.

Fig. 1e Wt growing on GYM 0,3M sucrose 48-hour culture. SYTO9 (green) and PI (red) staining.

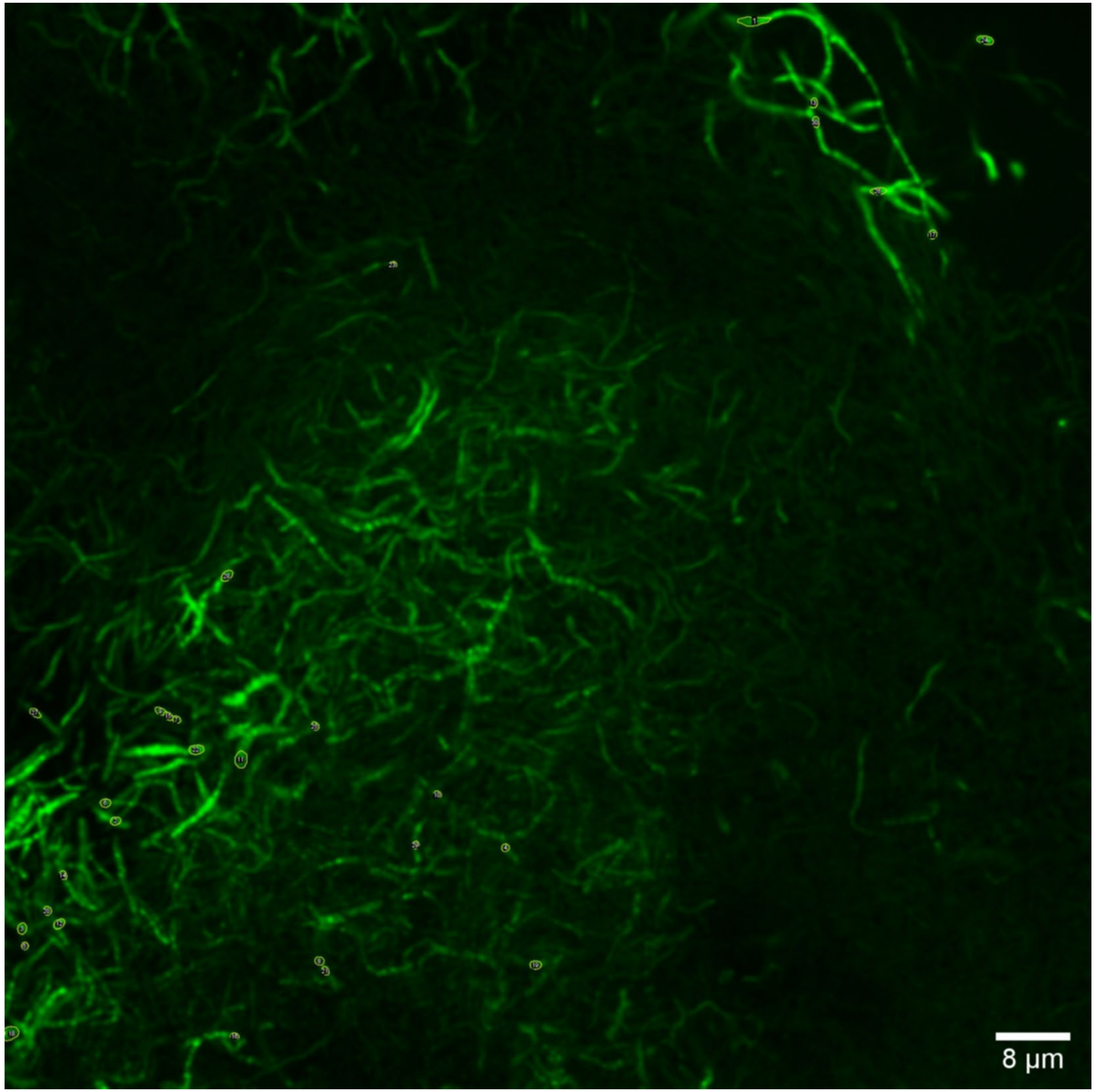

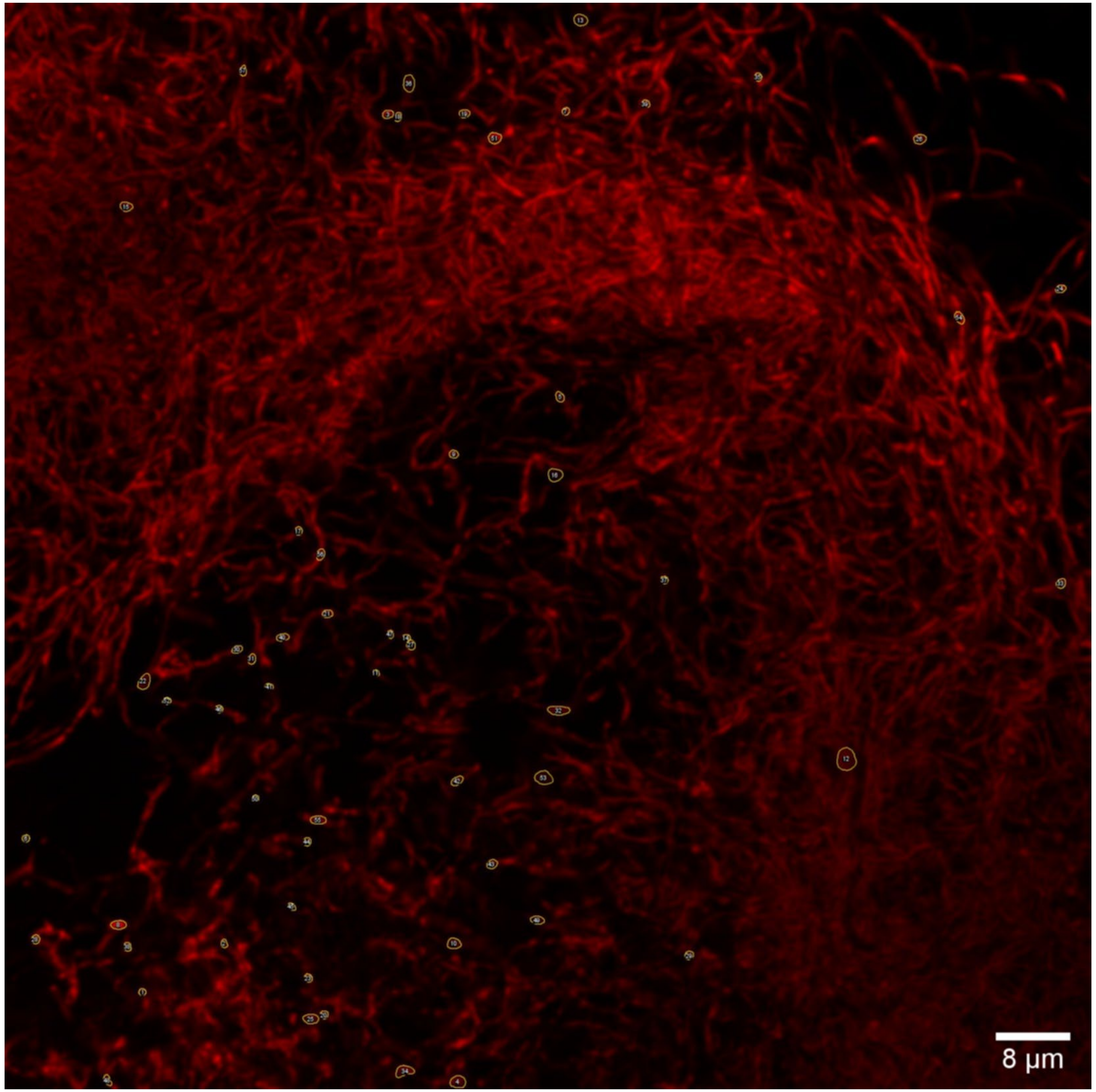

(a) Representative images of EVs used to quantify EV areas and to generate the histograms shown in Fig. 1.

Fig. 1f *sco1760::Tn5* growing on GYM 0,3M sucrose 48-hour culture. SYTO9 (green) and PI (red) staining.

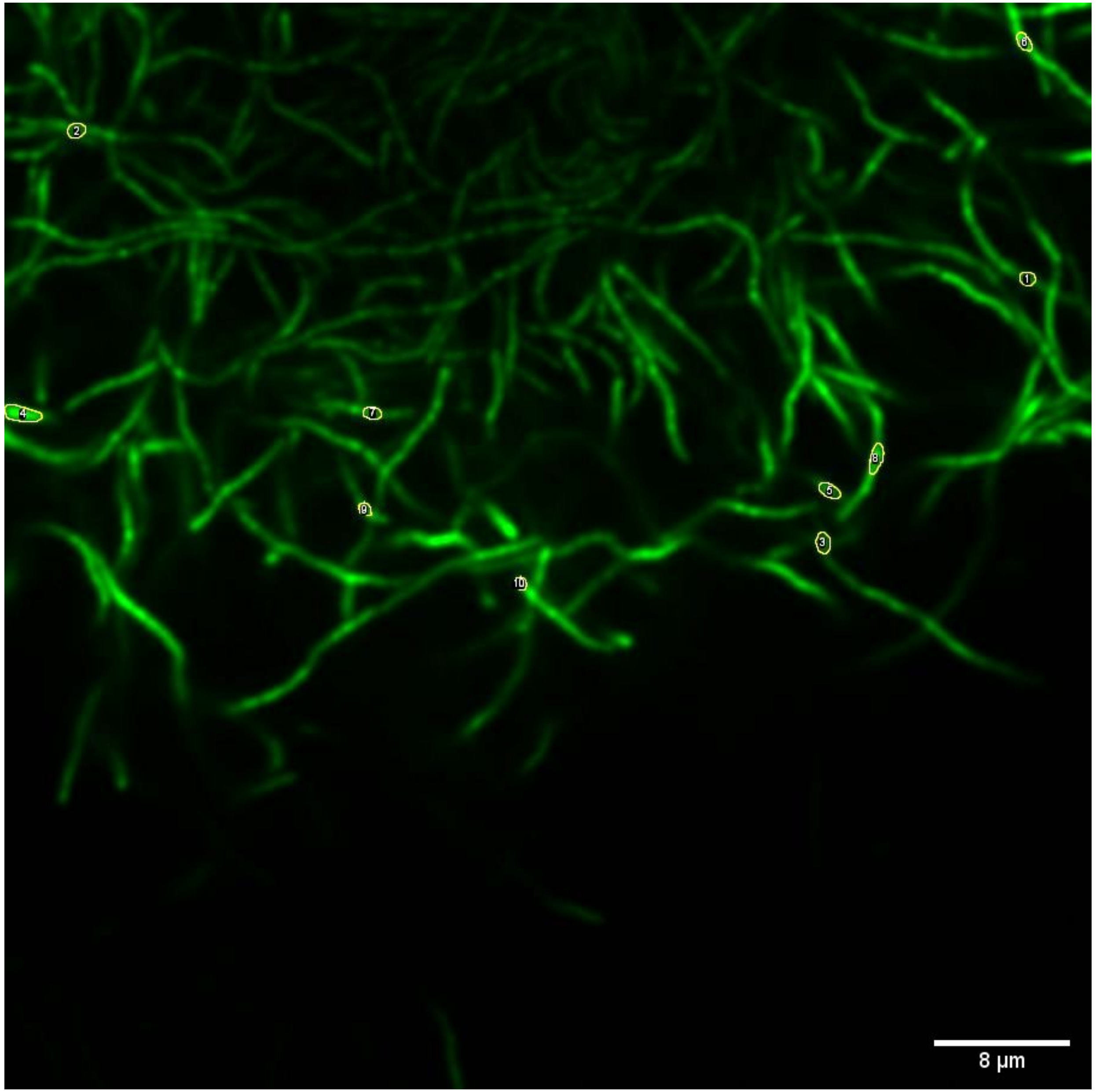

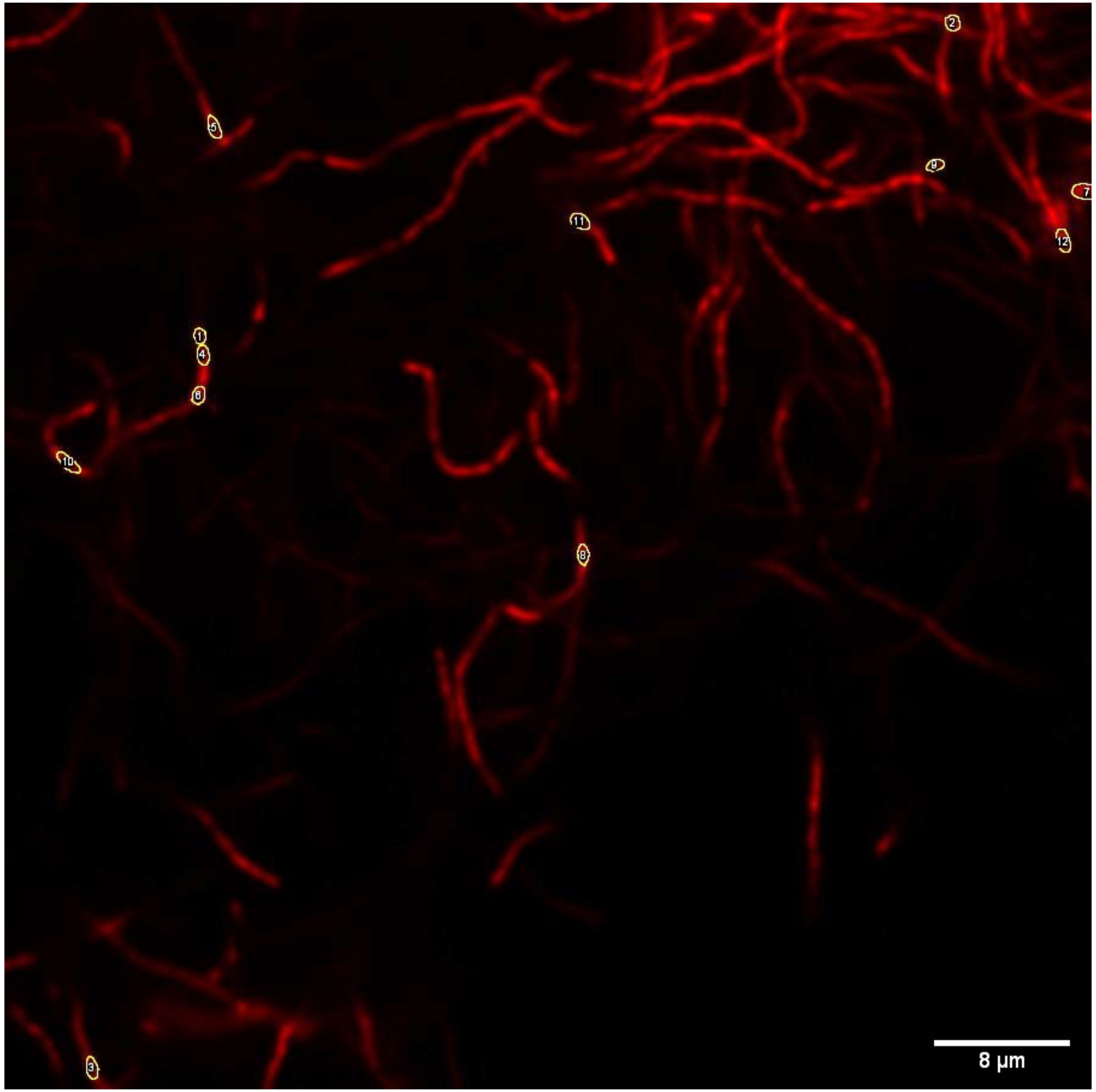

(a) Representative images of EVs used to quantify EV areas and to generate the histograms shown in Fig. 1.

Fig. 1g *sco4439/40::Tn5062* growing on GYM 0,3M sucrose 48-hour culture. SYTO9 (green) and PI (red) staining.

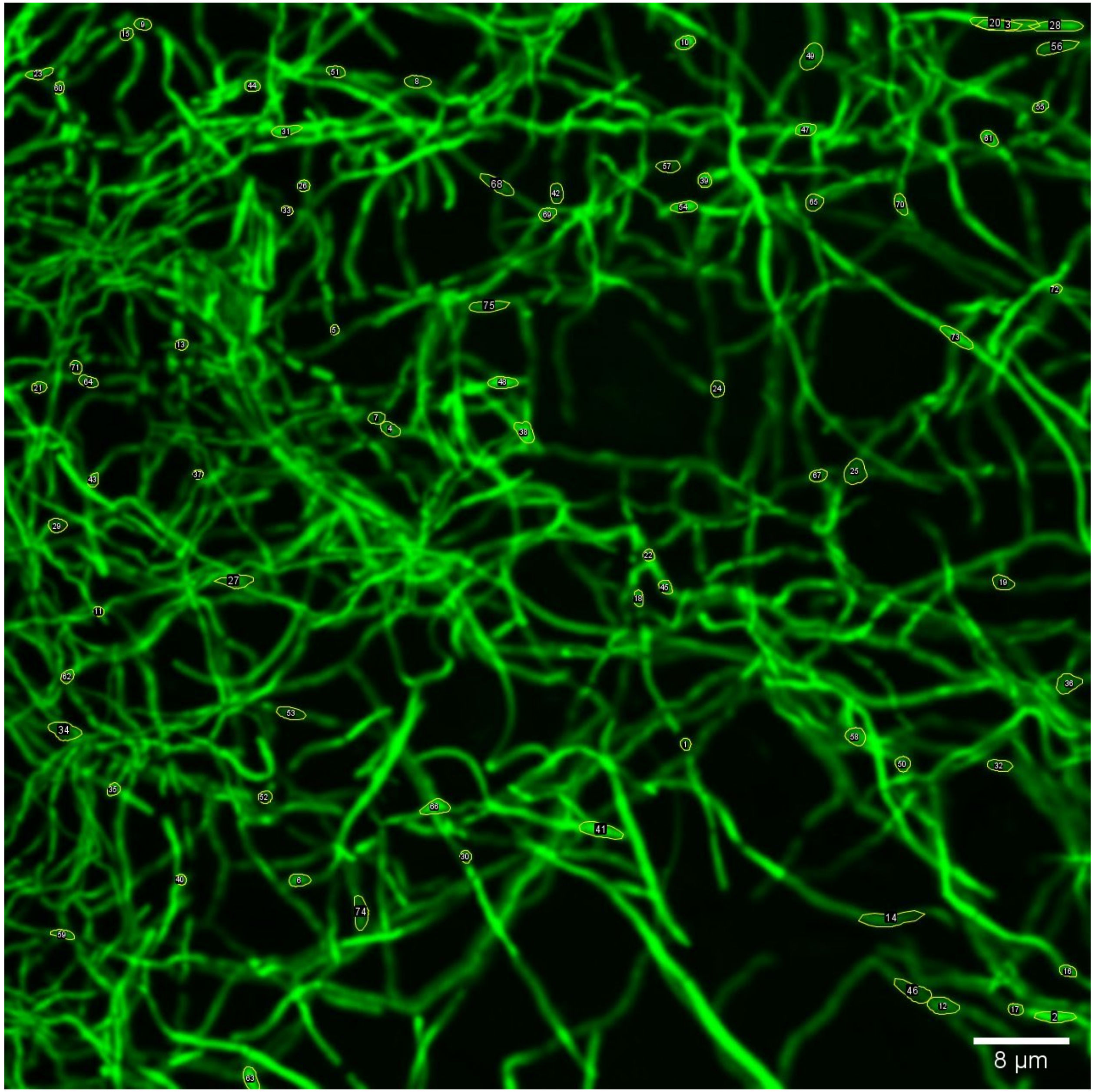

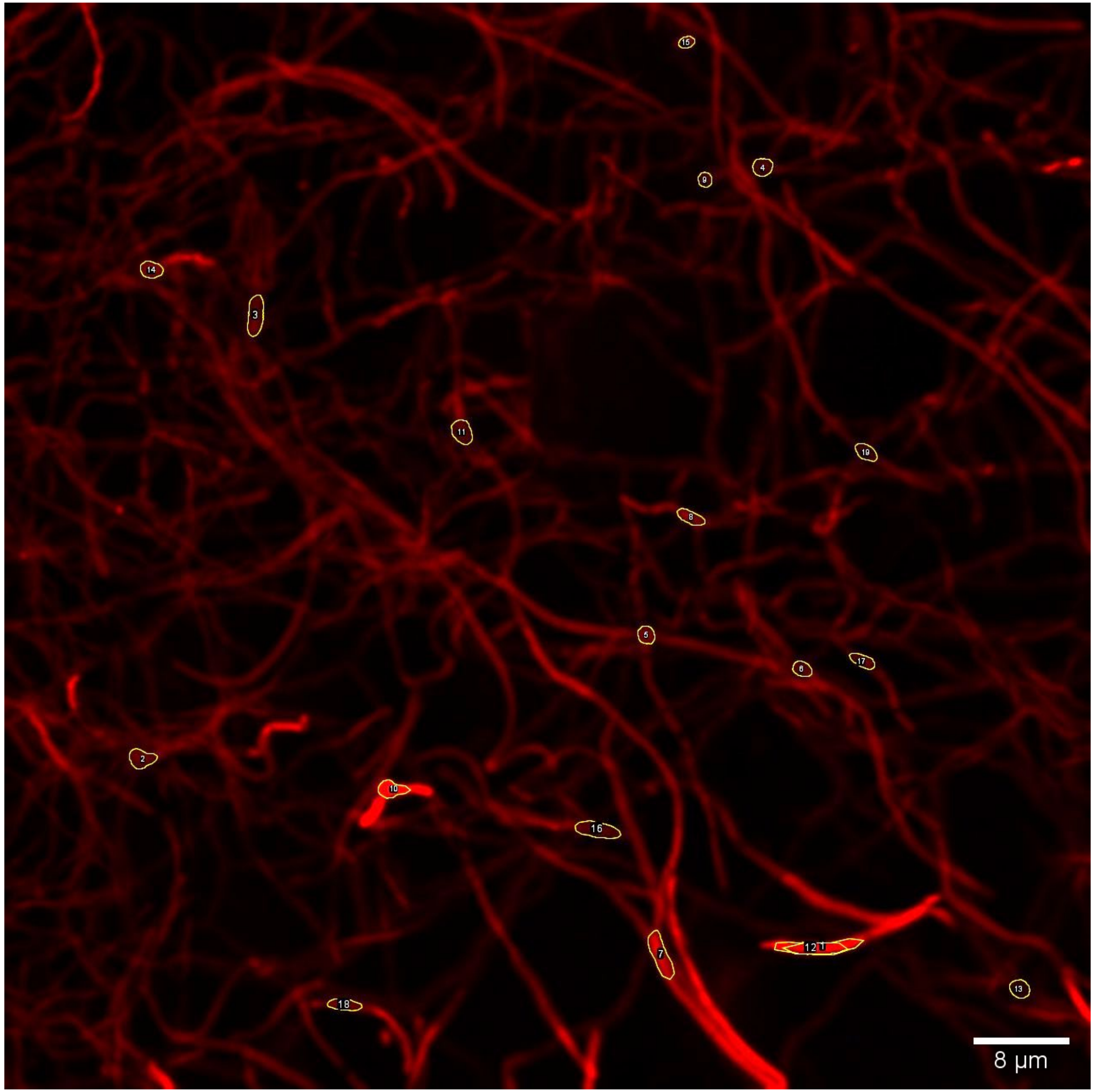

(a) Representative images of EVs used to quantify EV areas and to generate the histograms shown in Fig. 1.

Fig. 1h *S. coelicolor* overexpressing *sco0954* growing on GYM 0,3M sucrose 48-hour culture. SYTO9 (green) and PI (red) staining.

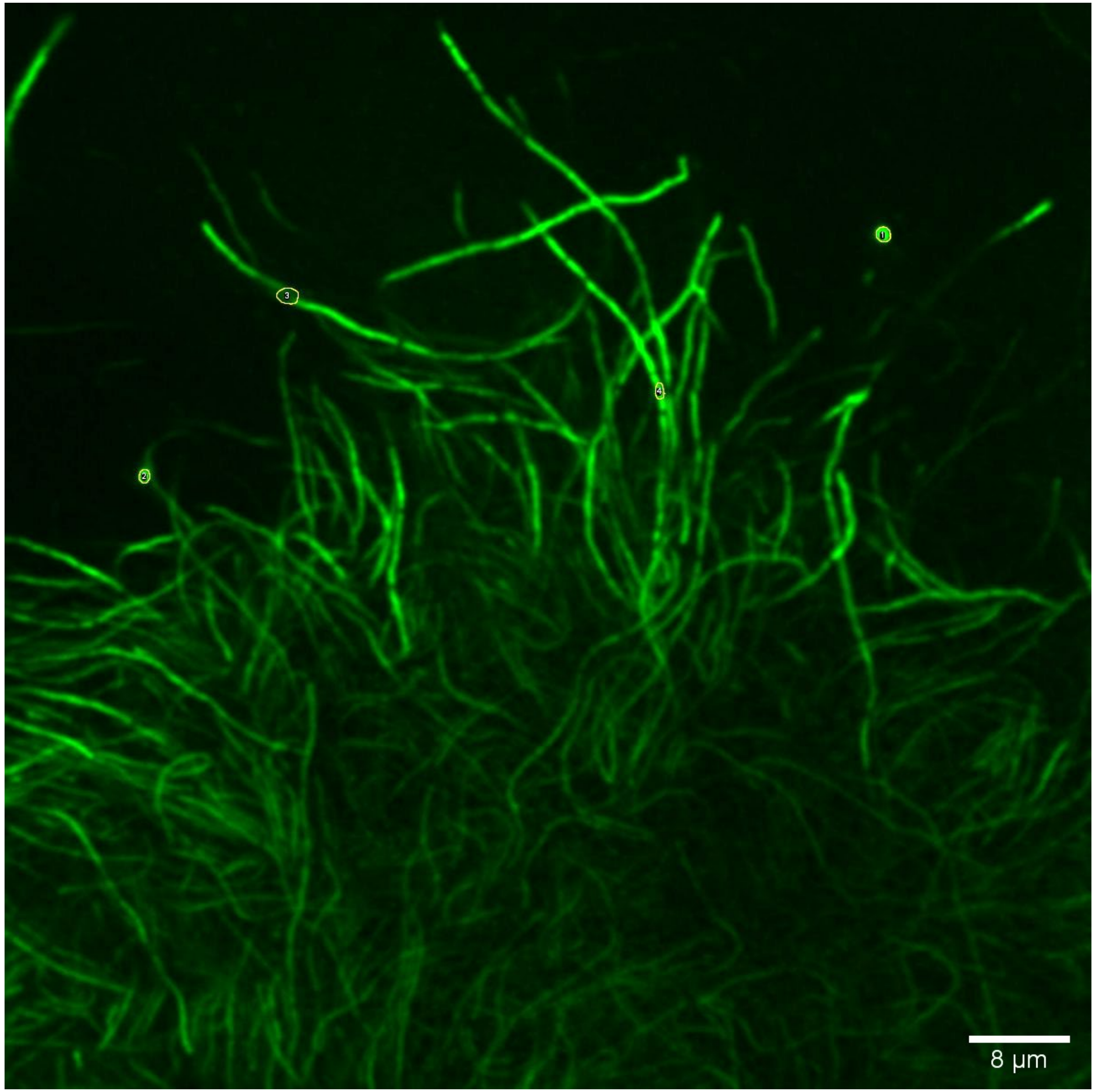

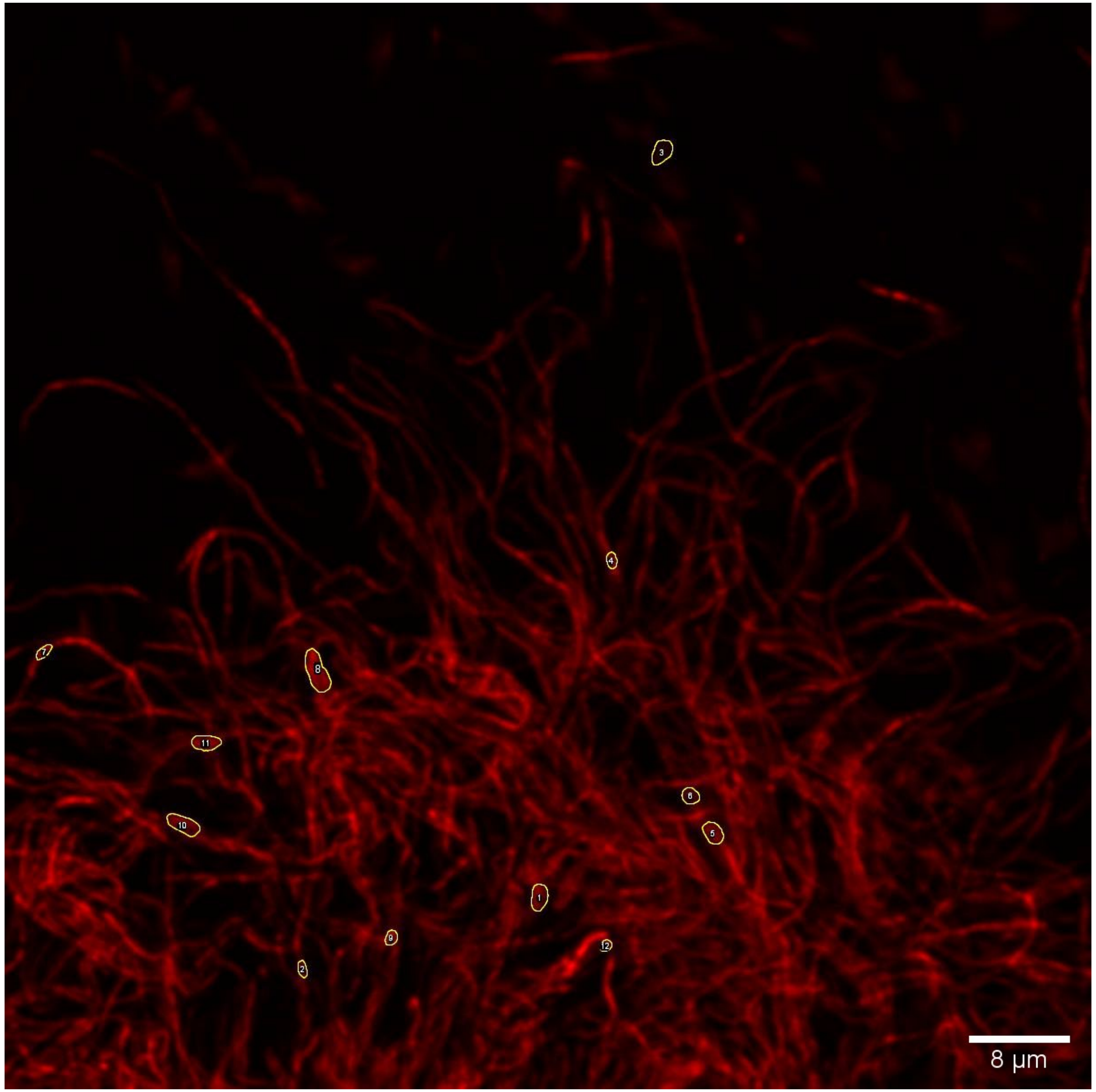

(b) Representative images of EVs used to generate the area histograms shown in Fig. 4.

Fig. 4b *sco1760::Tn5* pRASK *[sco1760]* growing on GYM 0,64M sucrose 48-hour culture. SYTO9 (green) and PI (red) staining.

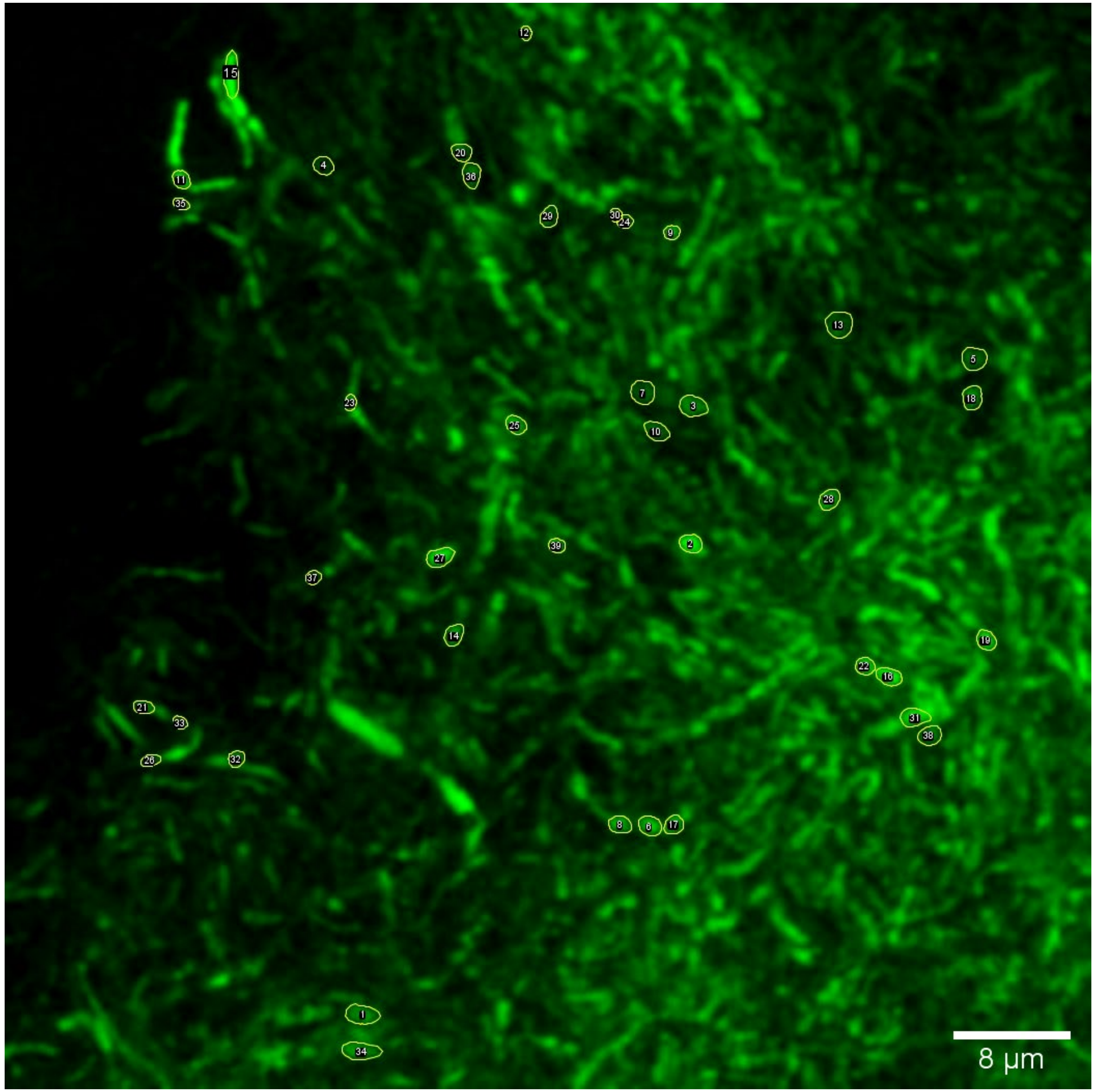

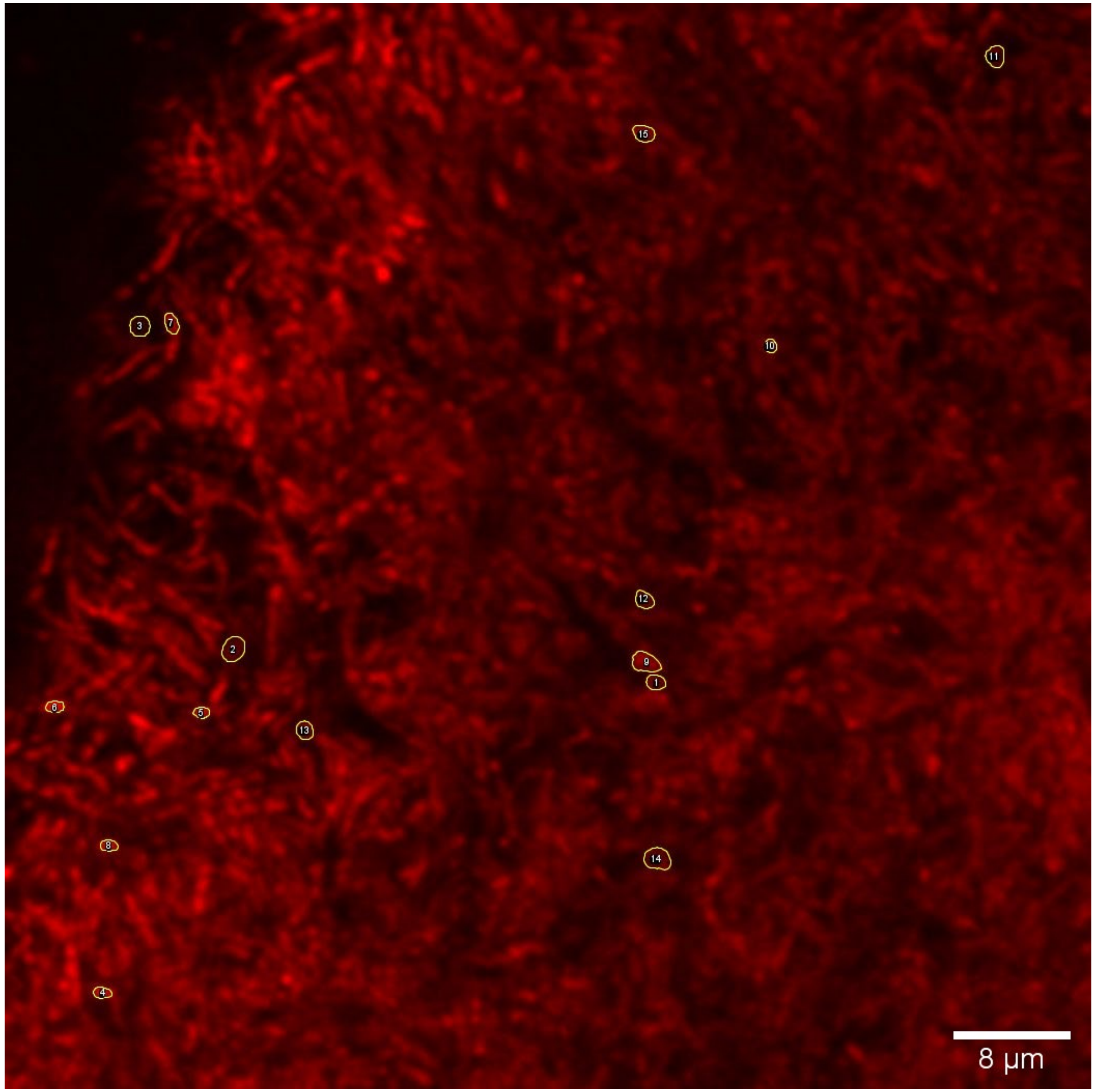

(b) Representative images of EVs used to generate the area histograms shown in Fig. 4.

Fig. 4c *sco1760::Tn5* pRASK *[sco1760-1759]* growing on GYM 0,64M sucrose 48-hour culture. SYTO9 (green) and PI (red) staining.

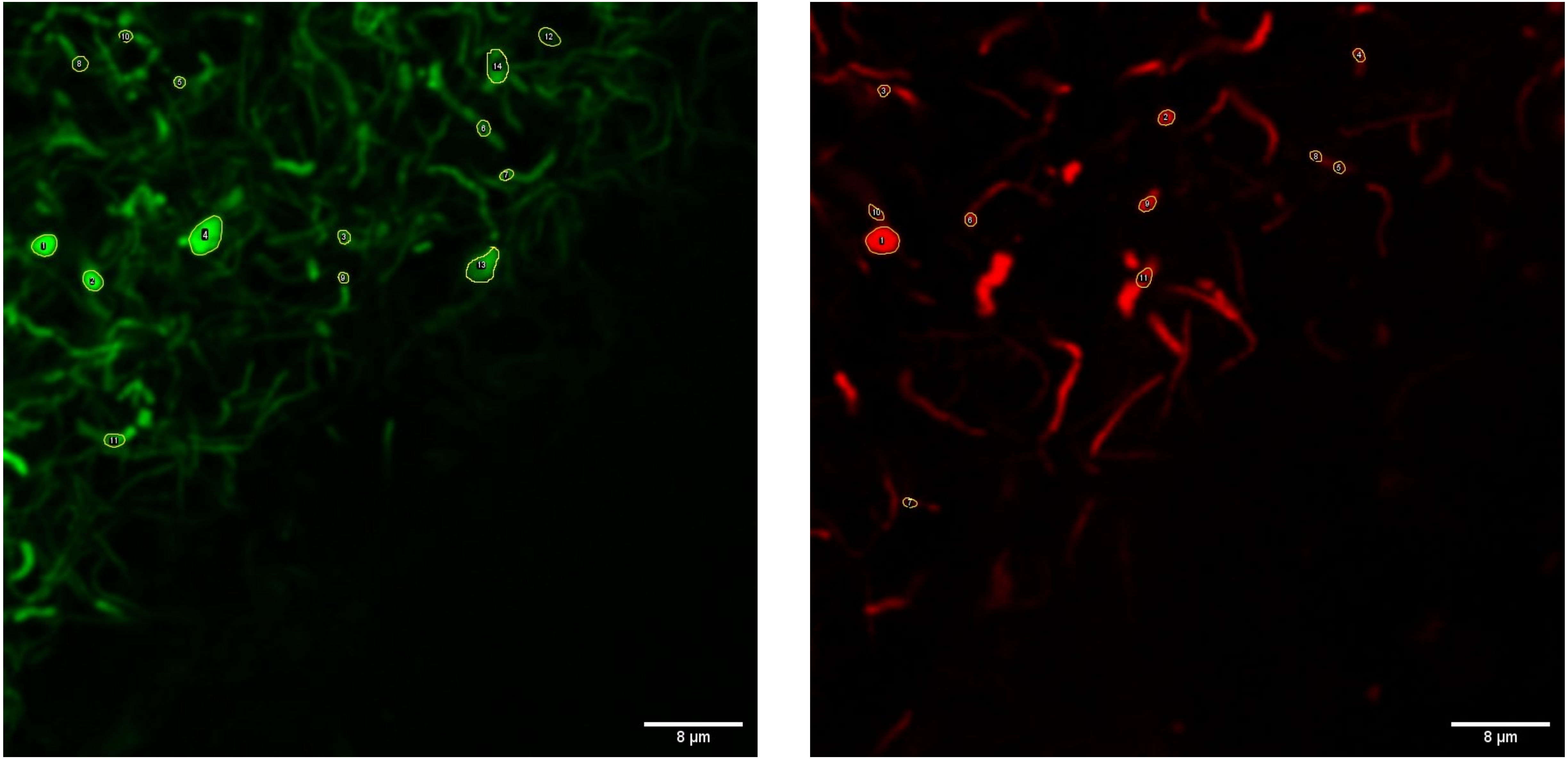

(b) Representative images of EVs used to generate the area histograms shown in Fig. 4.

Fig. 4d *sco1760::Tn5* pRASK *[sco1760-1758]* growing on GYM 0,64M sucrose 48-hour culture. SYTO9 (green) and PI (red) staining.

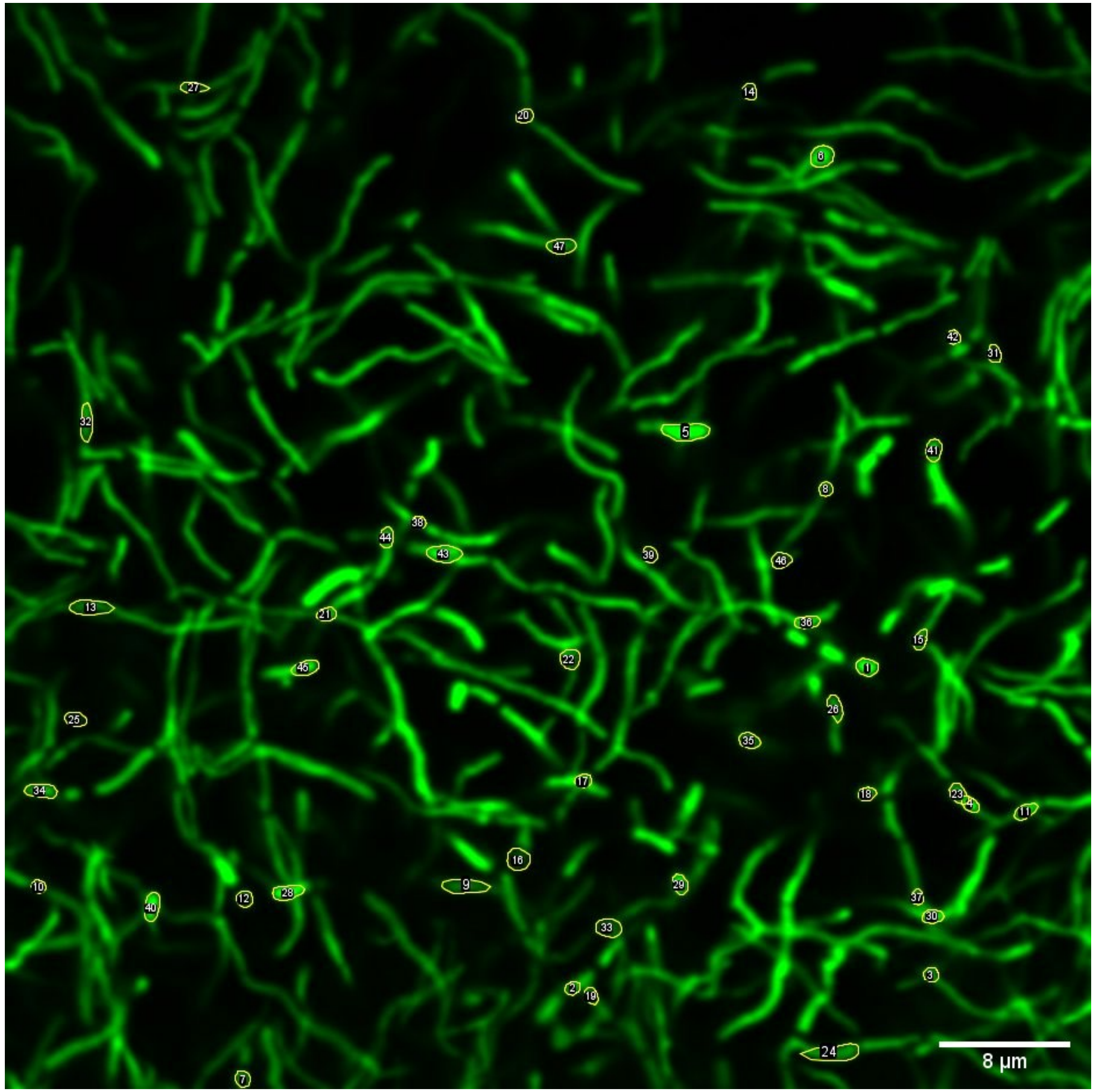

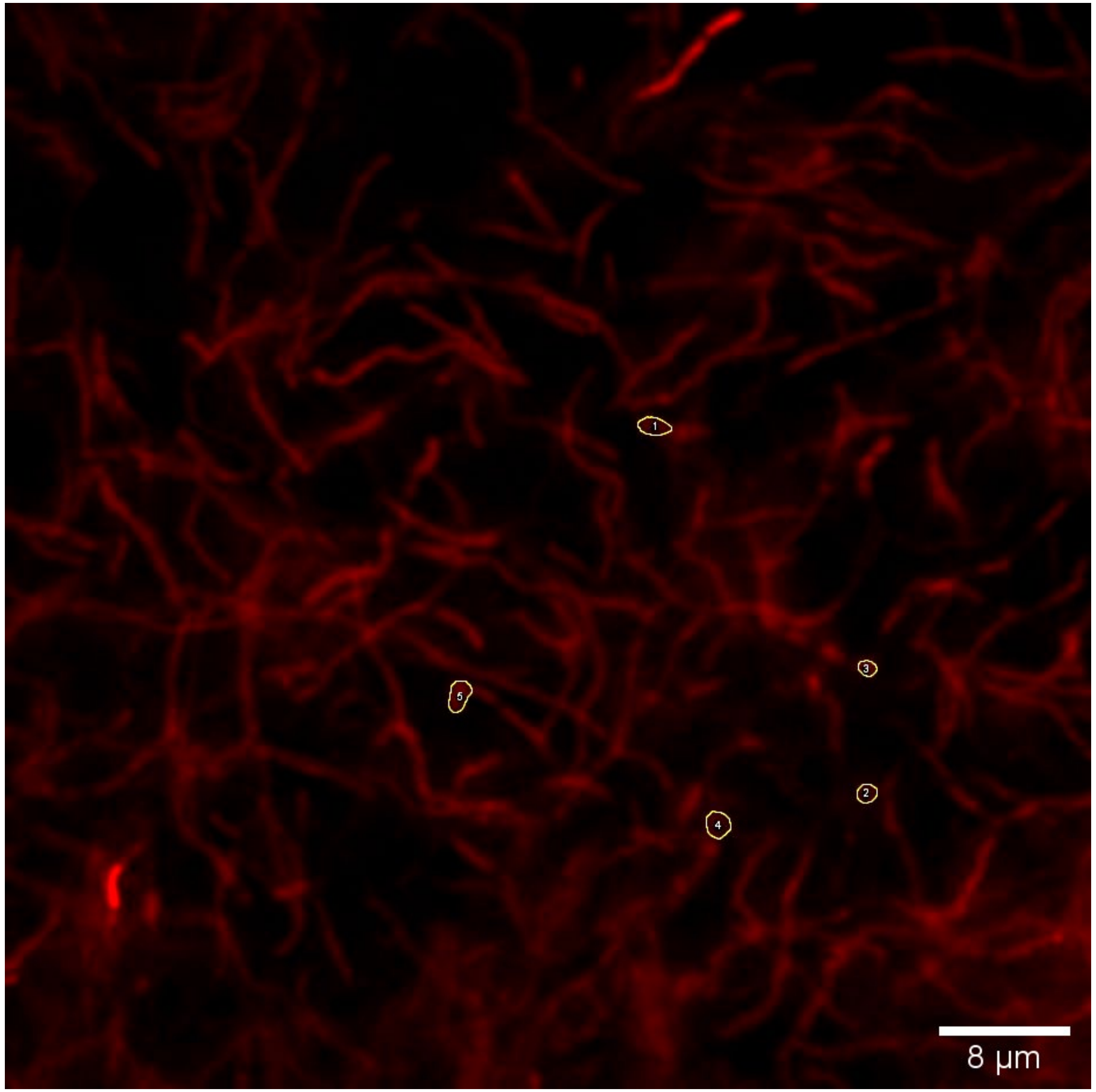

(b) Representative images of EVs used to generate the area histograms shown in Fig. 4.

Fig. 4f *sco4439/40::Tn5062* pRASK *[sco4439]* growing on GYM 0,64M sucrose 48-hour culture. SYTO9 (green) and PI (red) staining.

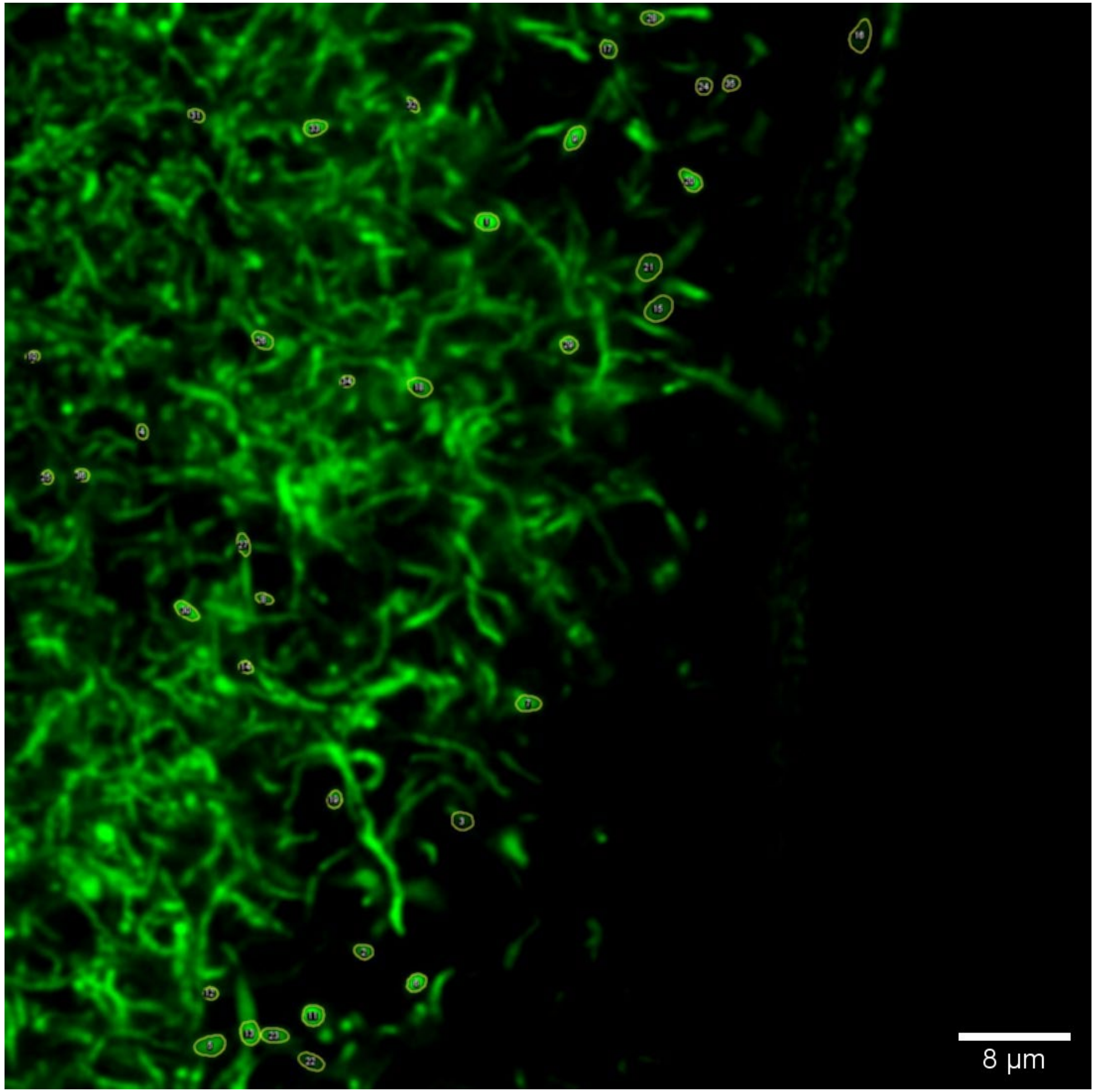

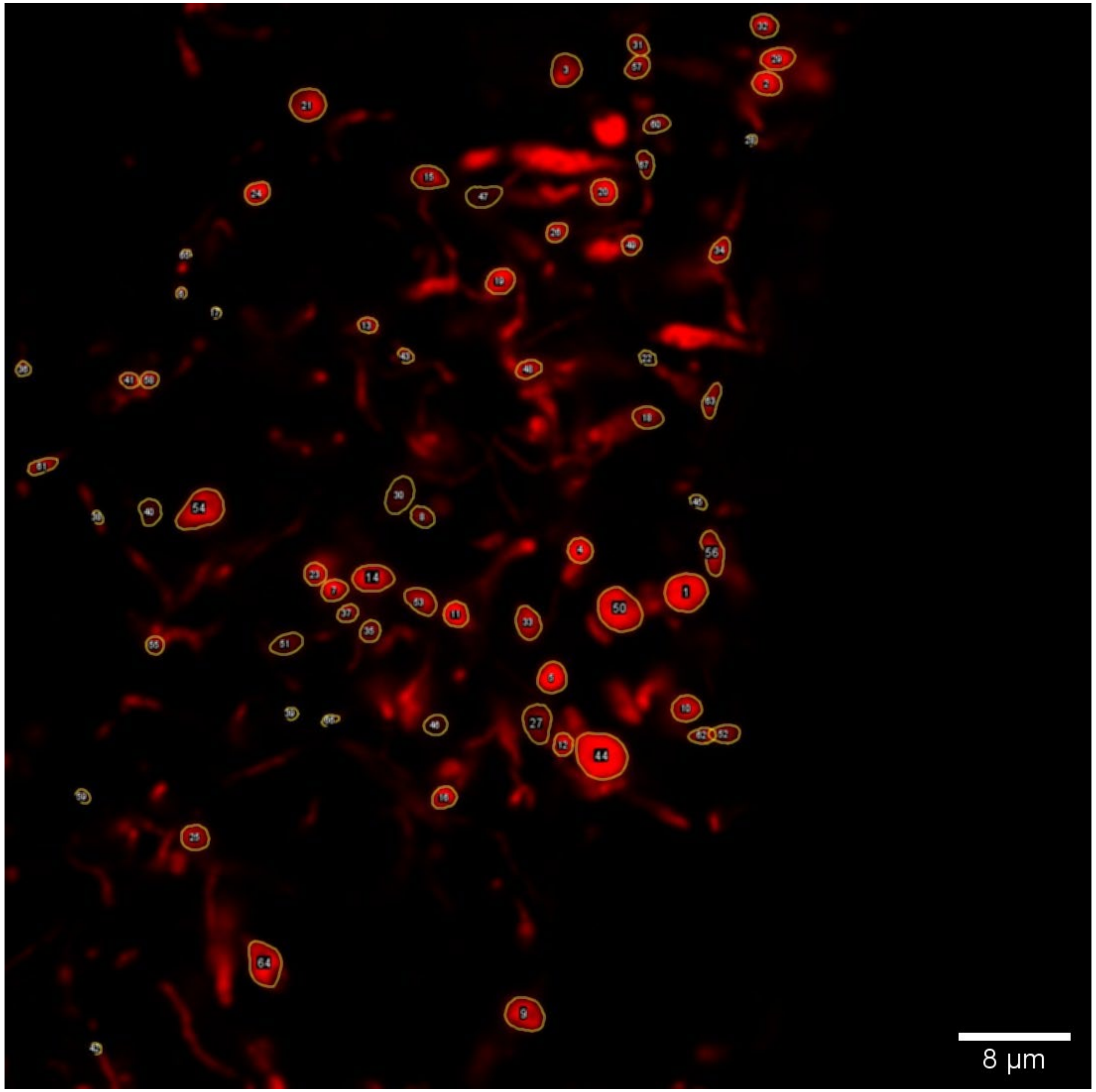

(b) Representative images of EVs used to generate the area histograms shown in Fig. 4.

Fig. 4g *sco4439/40::Tn5062* pRASK *[sco4440]* growing on GYM 0,64M sucrose 48-hour culture. SYTO9 (green) and PI (red) staining.

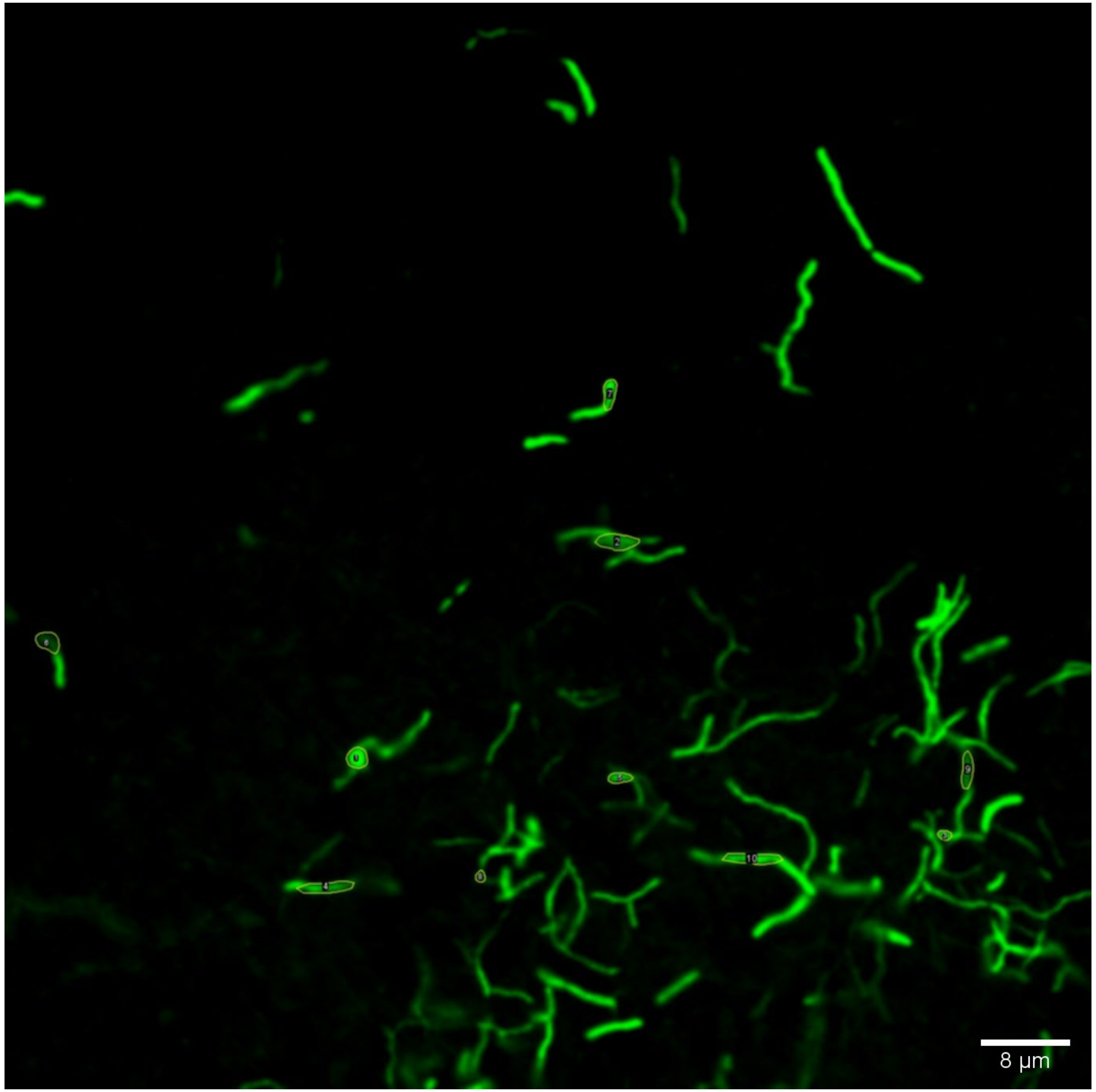

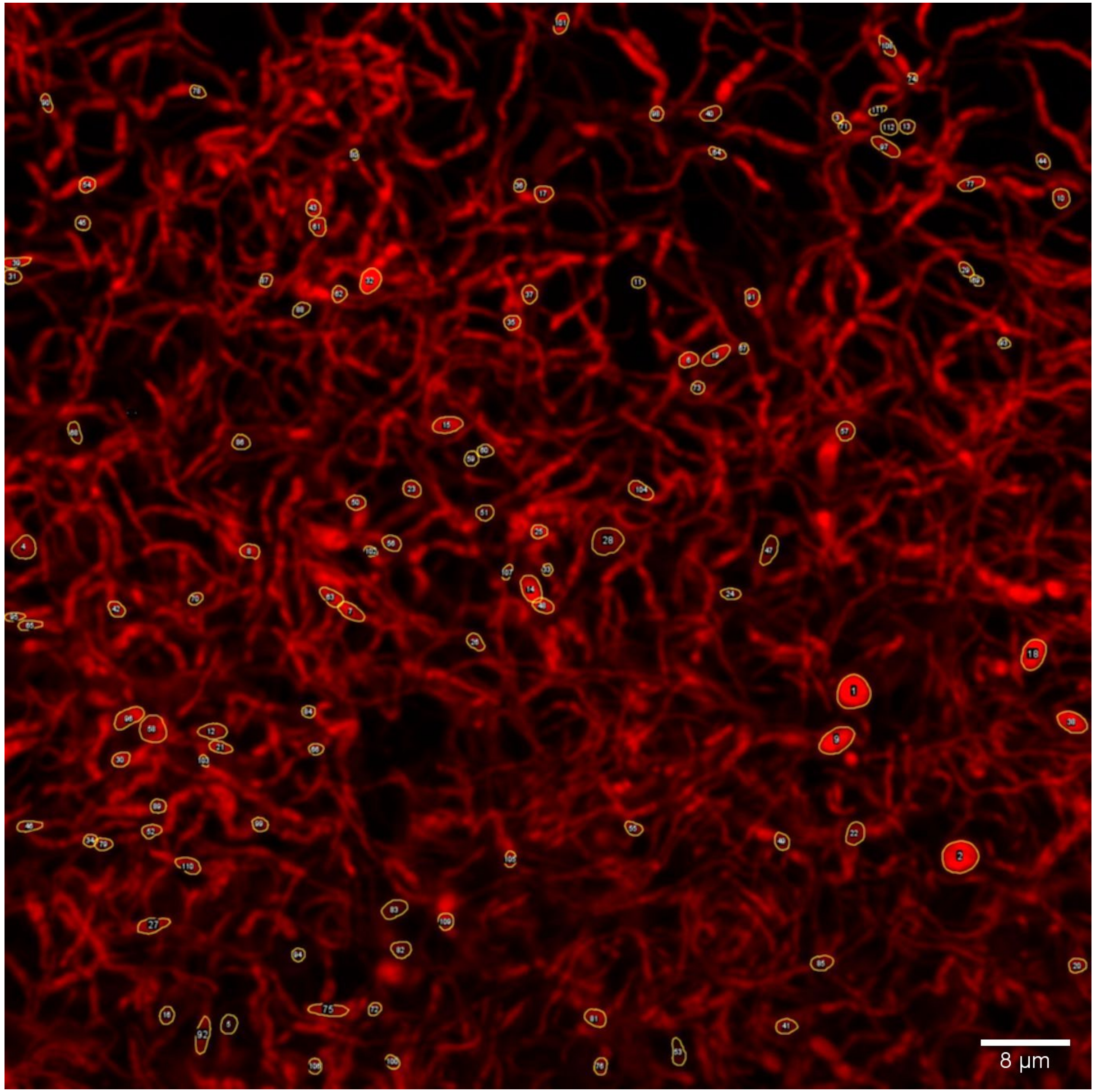

(b) Representative images of EVs used to generate the area histograms shown in Fig. 4.

Fig. 4h *sco4439/40::Tn5062* pRASK *[sco4439-4440]* growing on GYM 0,64M sucrose 48-hour culture. SYTO9 (green) and PI (red) staining.

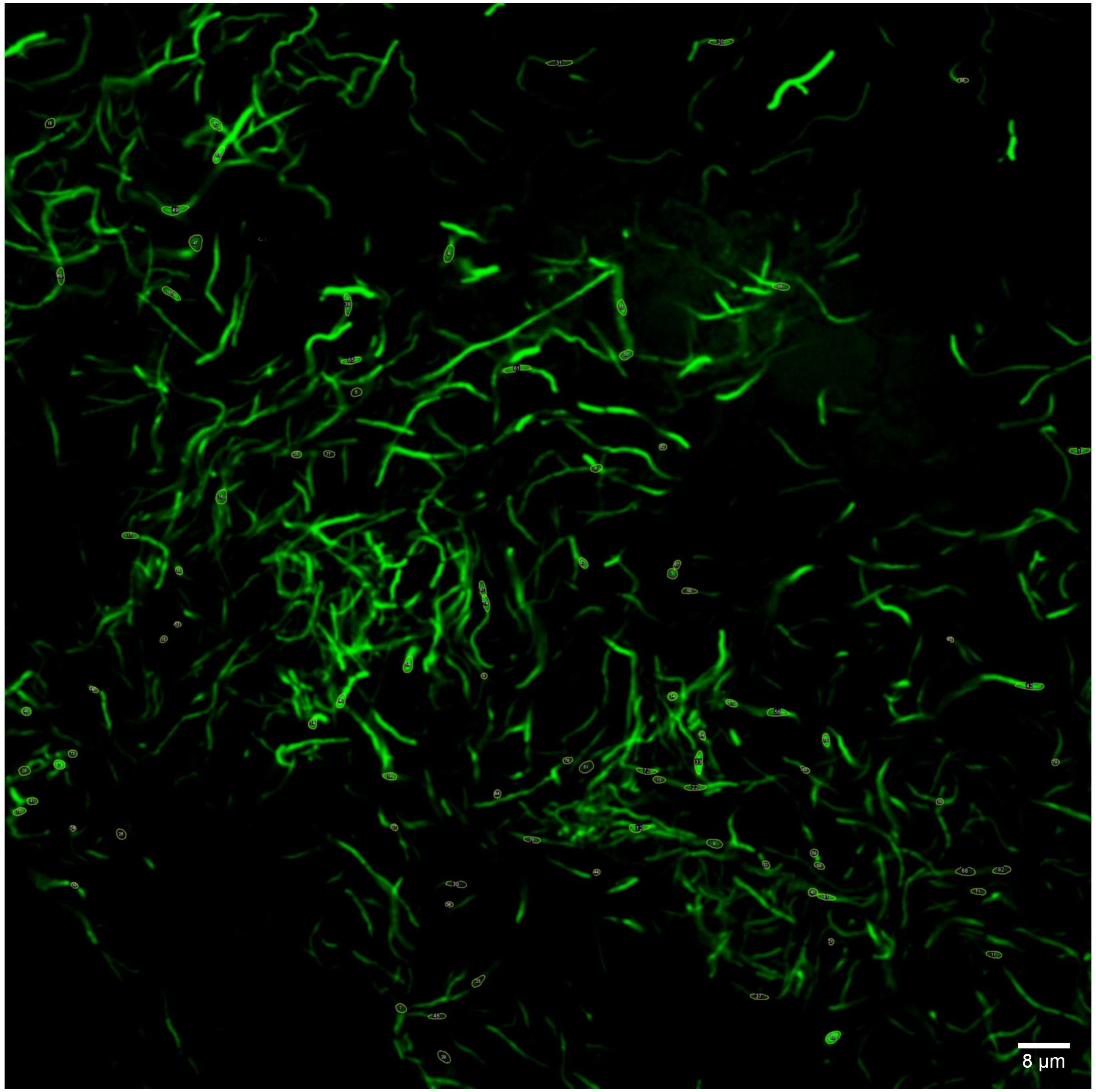

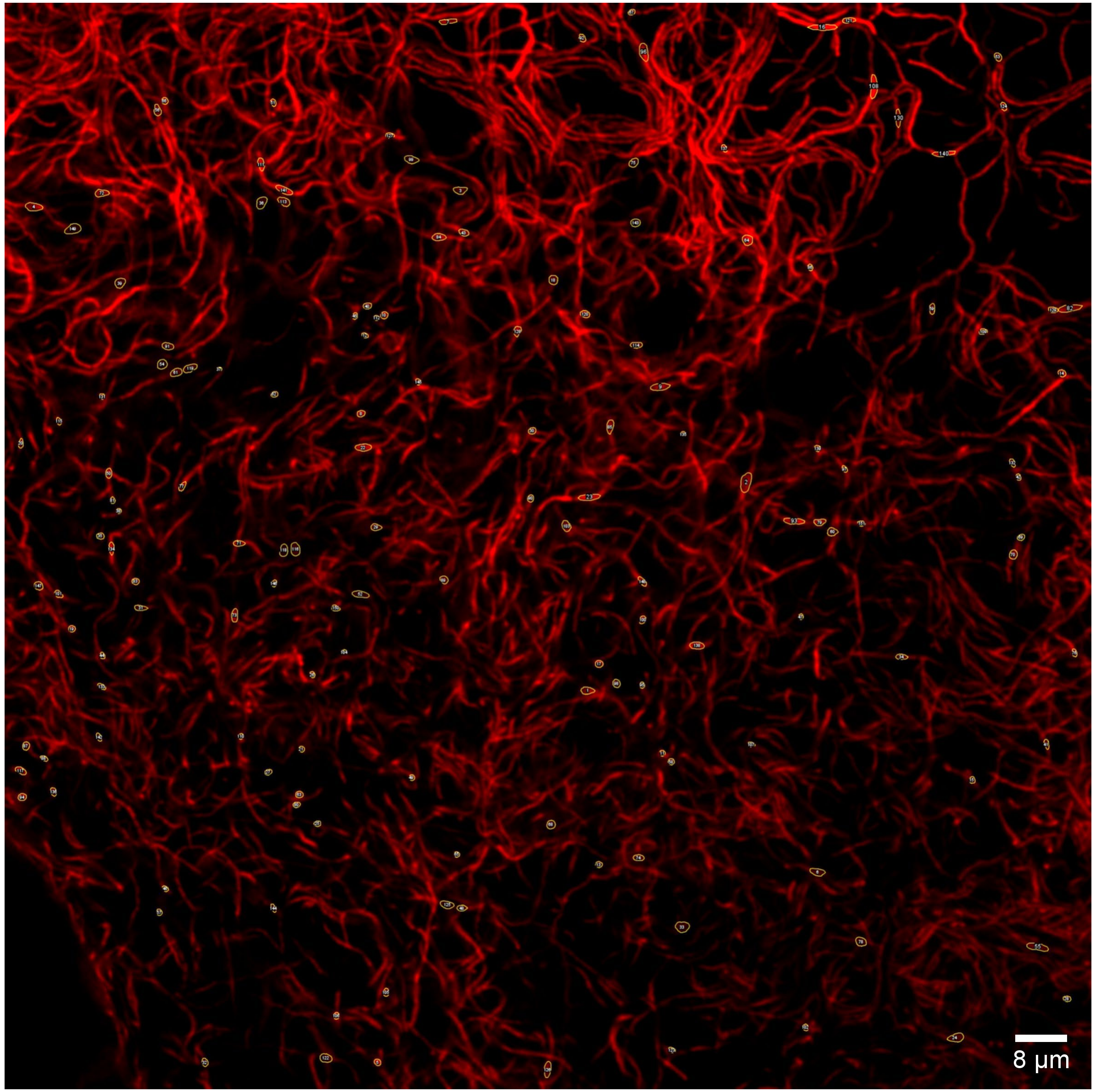

(b) Representative images of EVs used to generate the area histograms shown in Fig. 4.

Fig. 4i wt pRASK growing on GYM 0,64M sucrose 48-hour culture. SYTO9 (green) and PI (red) staining.

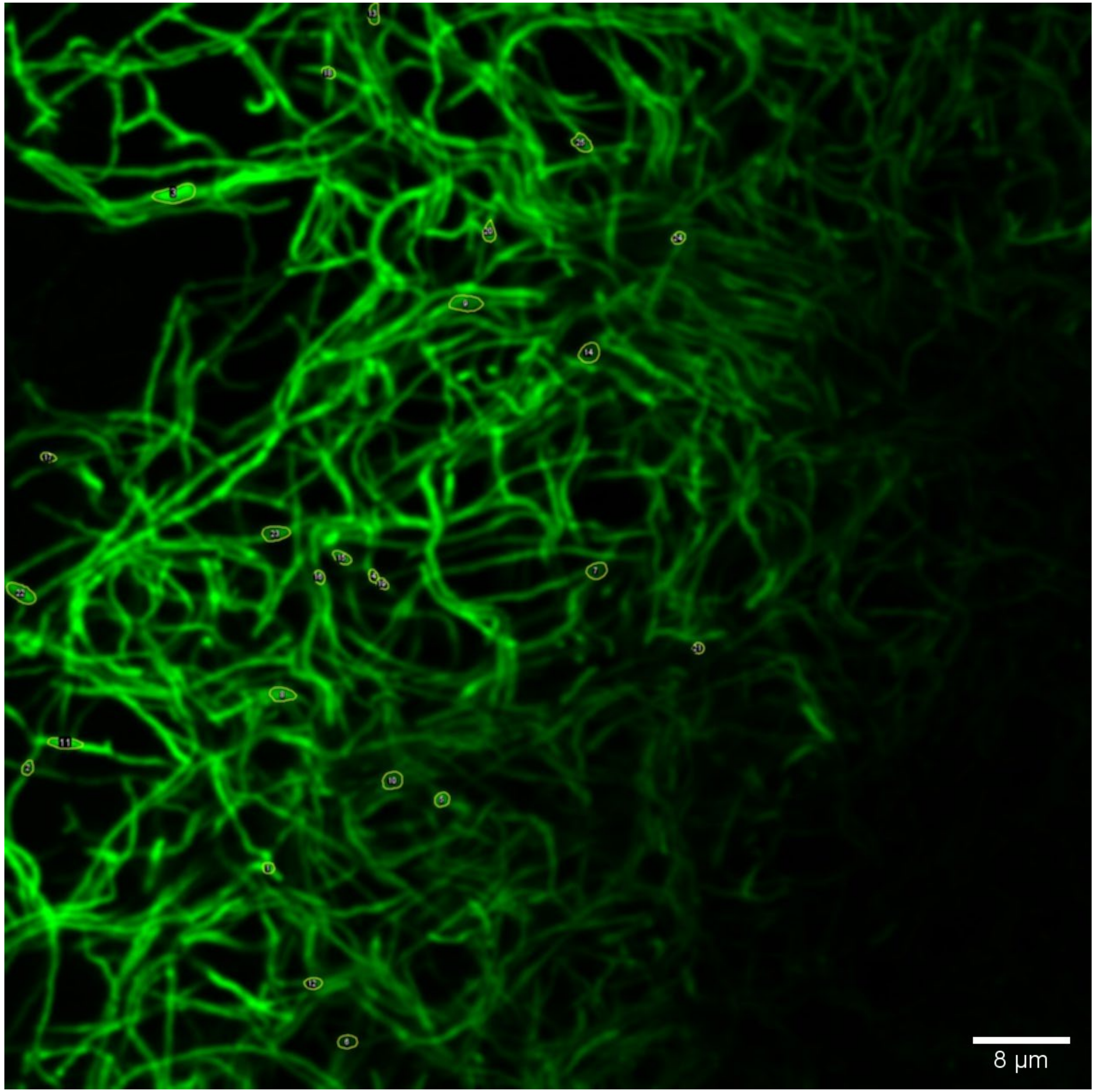

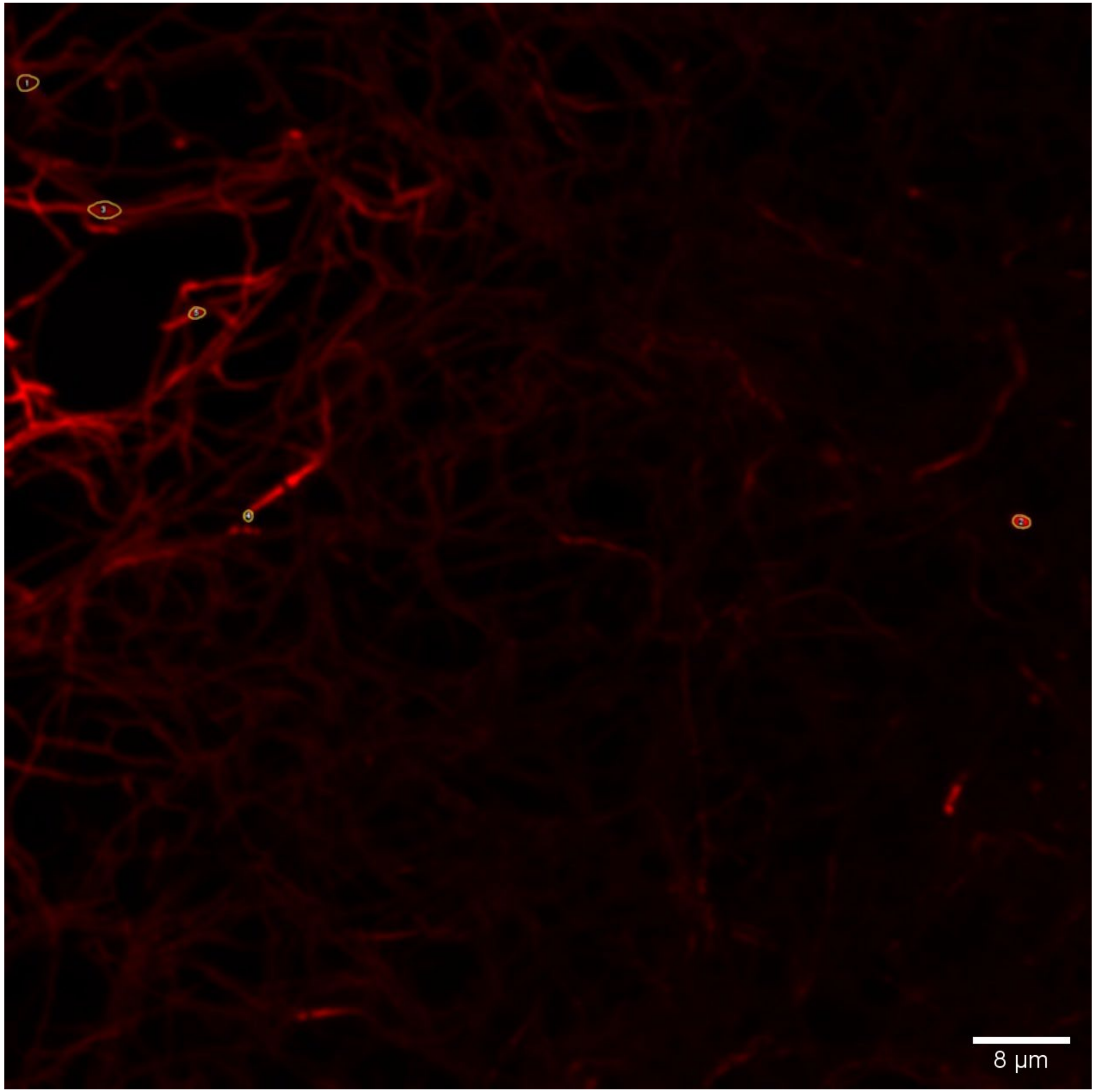

(b) Images of EVs used to generate the area histograms shown in Fig. 4.

Fig. 4j wt pMS82 growing on GYM 0,64M sucrose 48-hour culture. SYTO9 (green) and PI (red) staining.

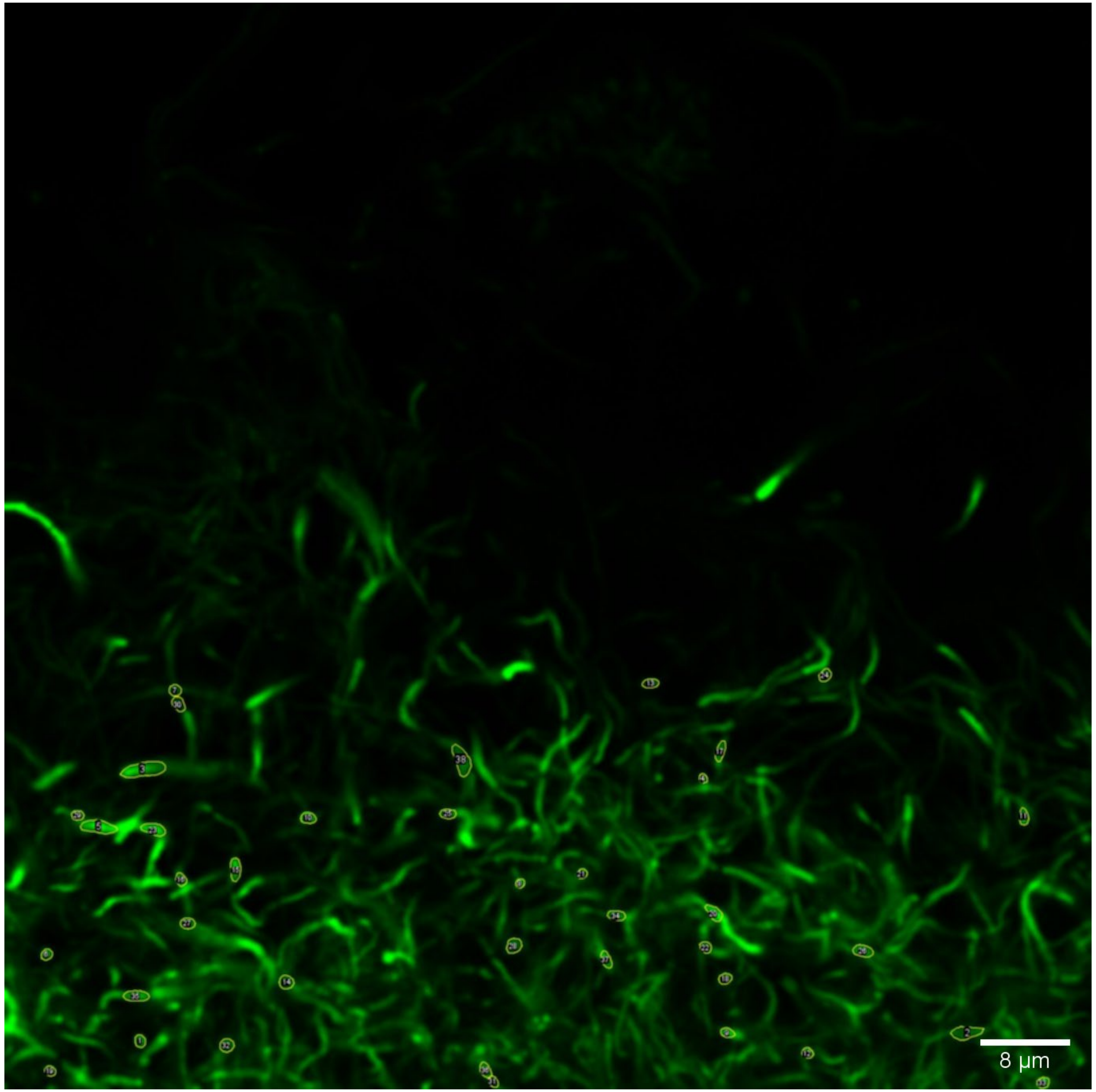

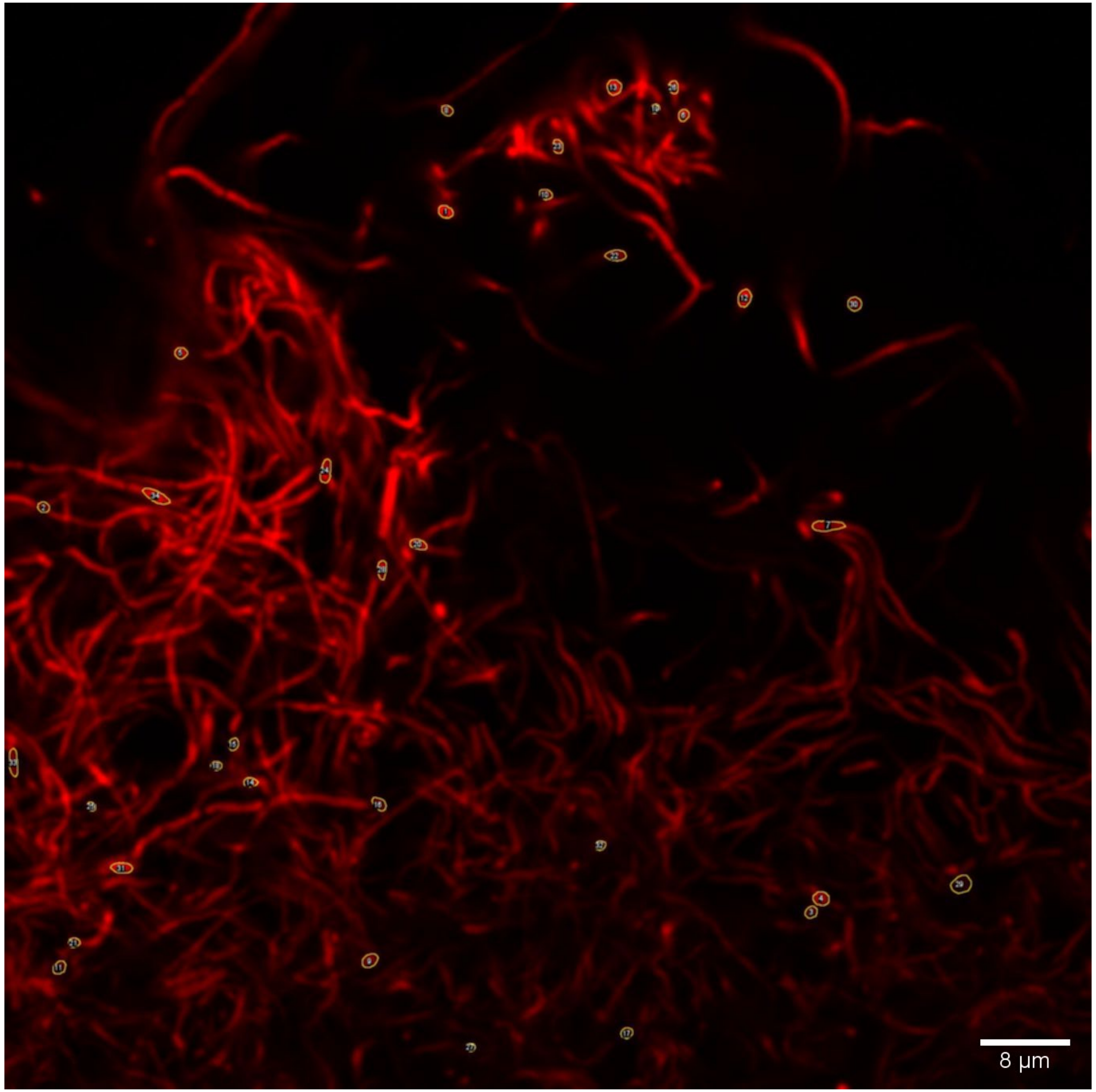

(c) Images used to calculate the membrane/cell wall ratio, as presented in the histogram in Fig. 2h.

Fig. 2a *S. coelicolor* wild-type strain growing on GYM 0,64M sucrose 48-hour culture. WGA (green) and FM595 (red) staining.

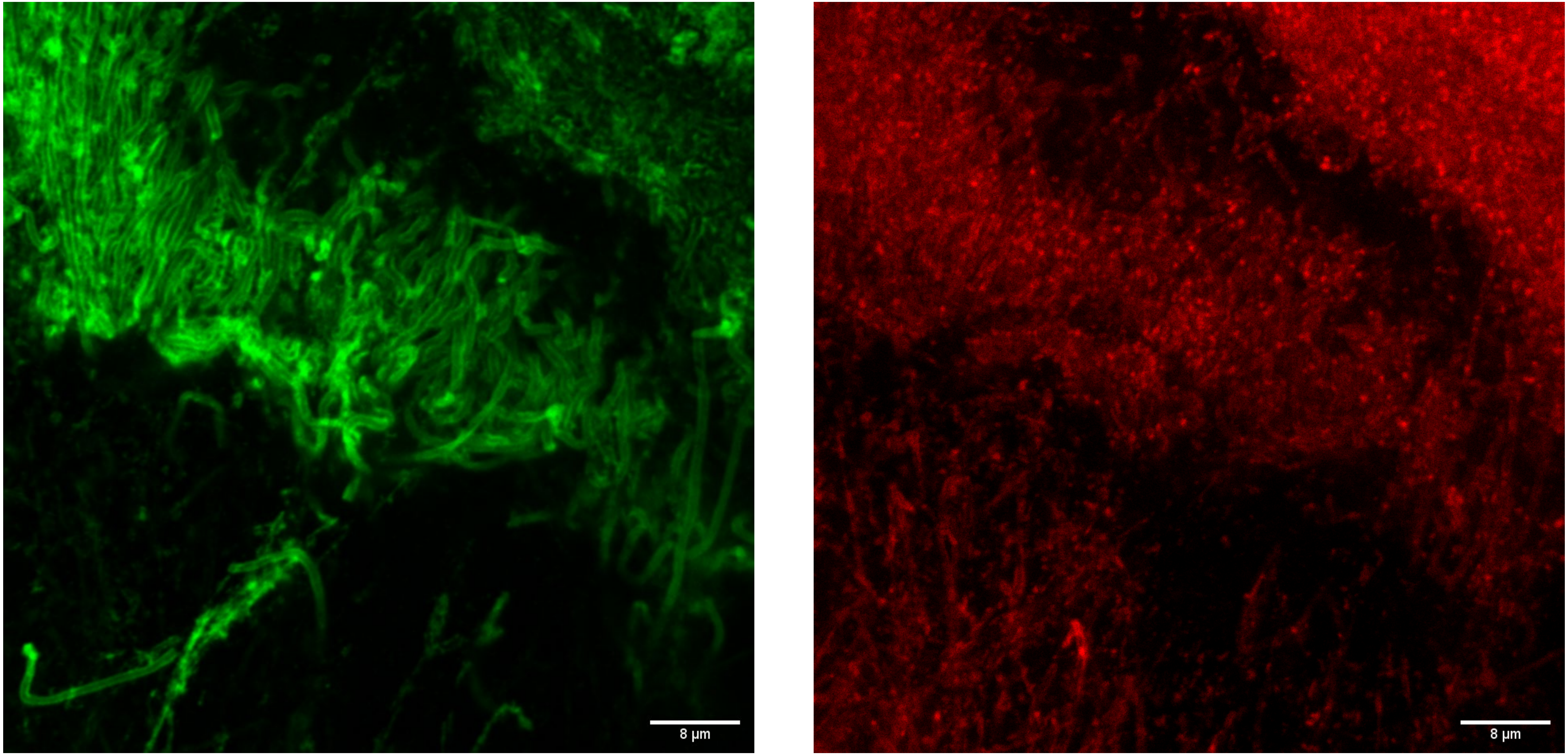

(c) Representative images used to calculate the membrane/cell wall ratio, as presented in the histogram in Fig. 2h.

Fig. 2b,c *S. coelicolor* overexpressing *sco0954* growing on GYM 0,64M sucrose 48-hour culture. WGA (green) and FM595 (red) staining.

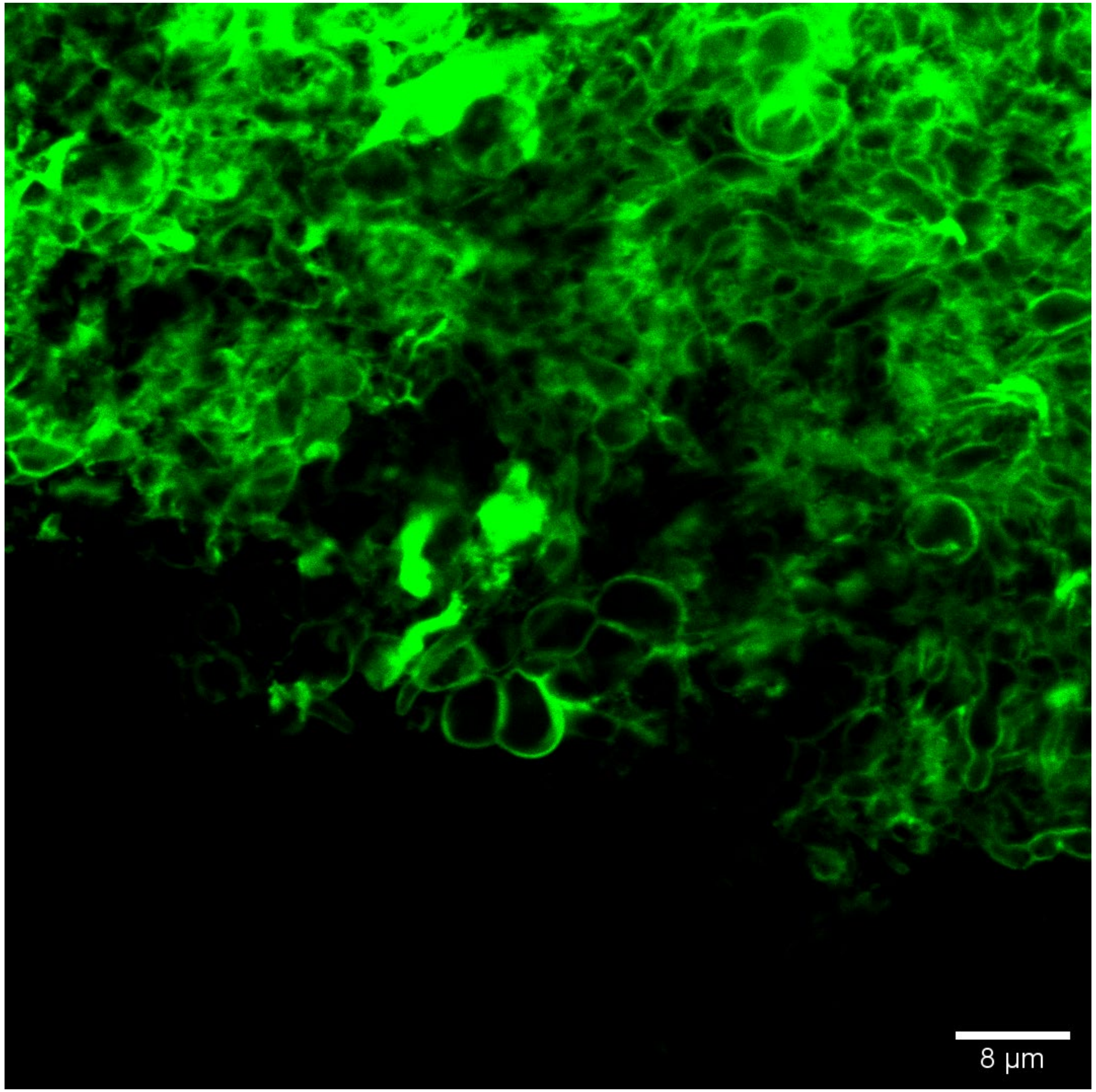

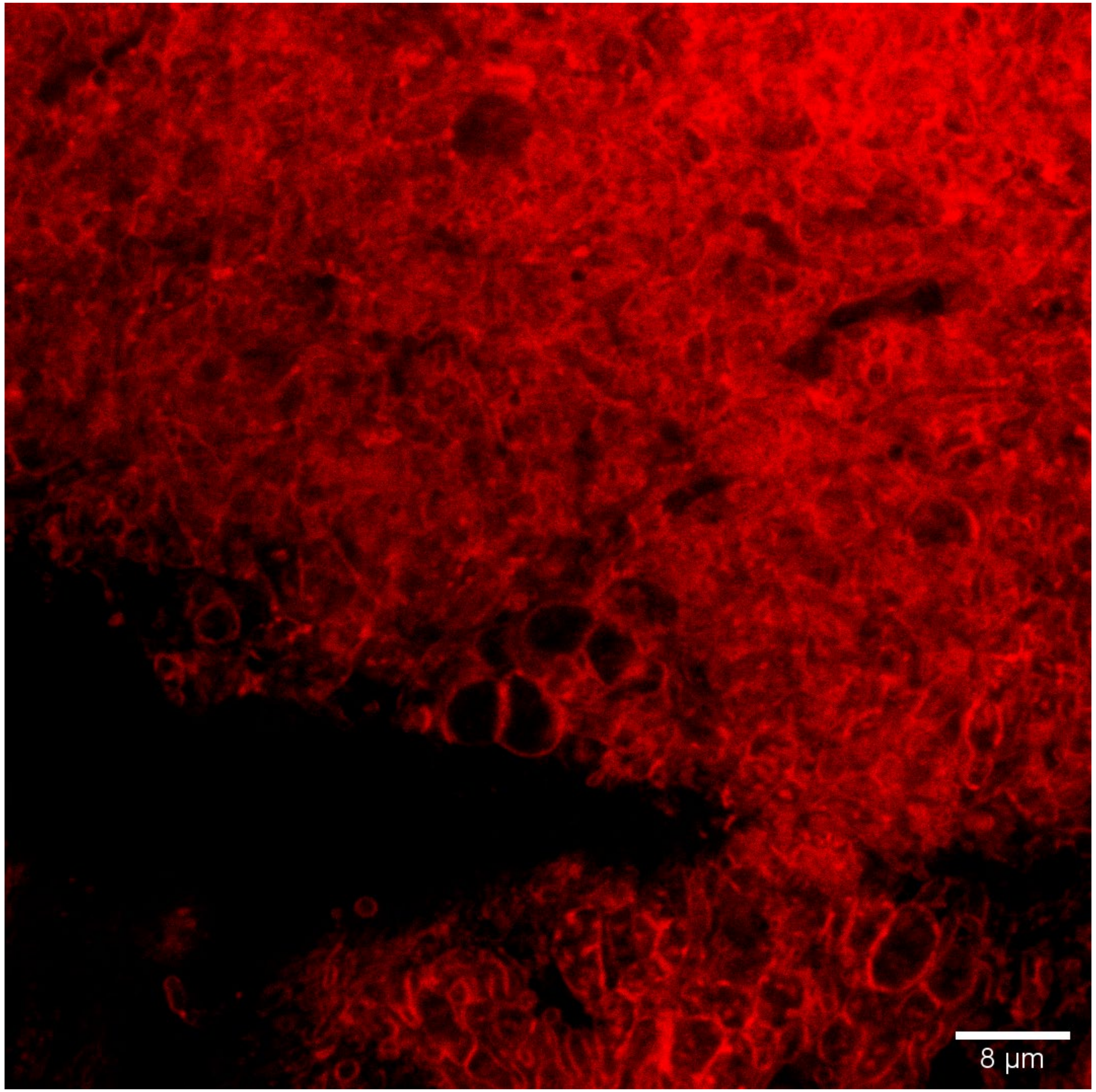

(c) Representative images used to calculate the membrane/cell wall ratio, as presented in the histogram in Fig. 2h.

Fig. 2d, e *sco4439/40::Tn5062* mutant growing on GYM 0,64M sucrose 48-hour culture. WGA (green) and FM595 (red) staining.

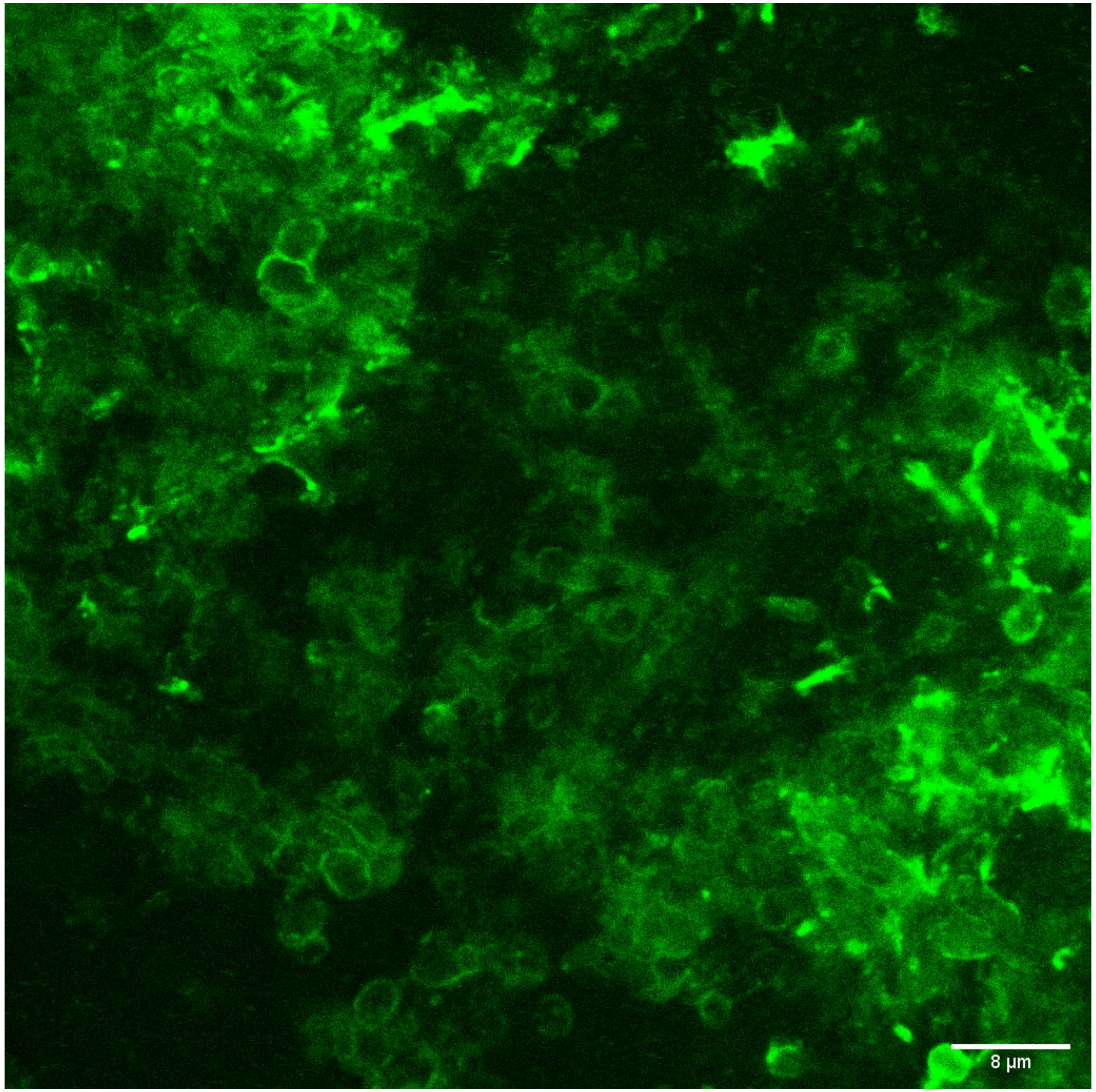

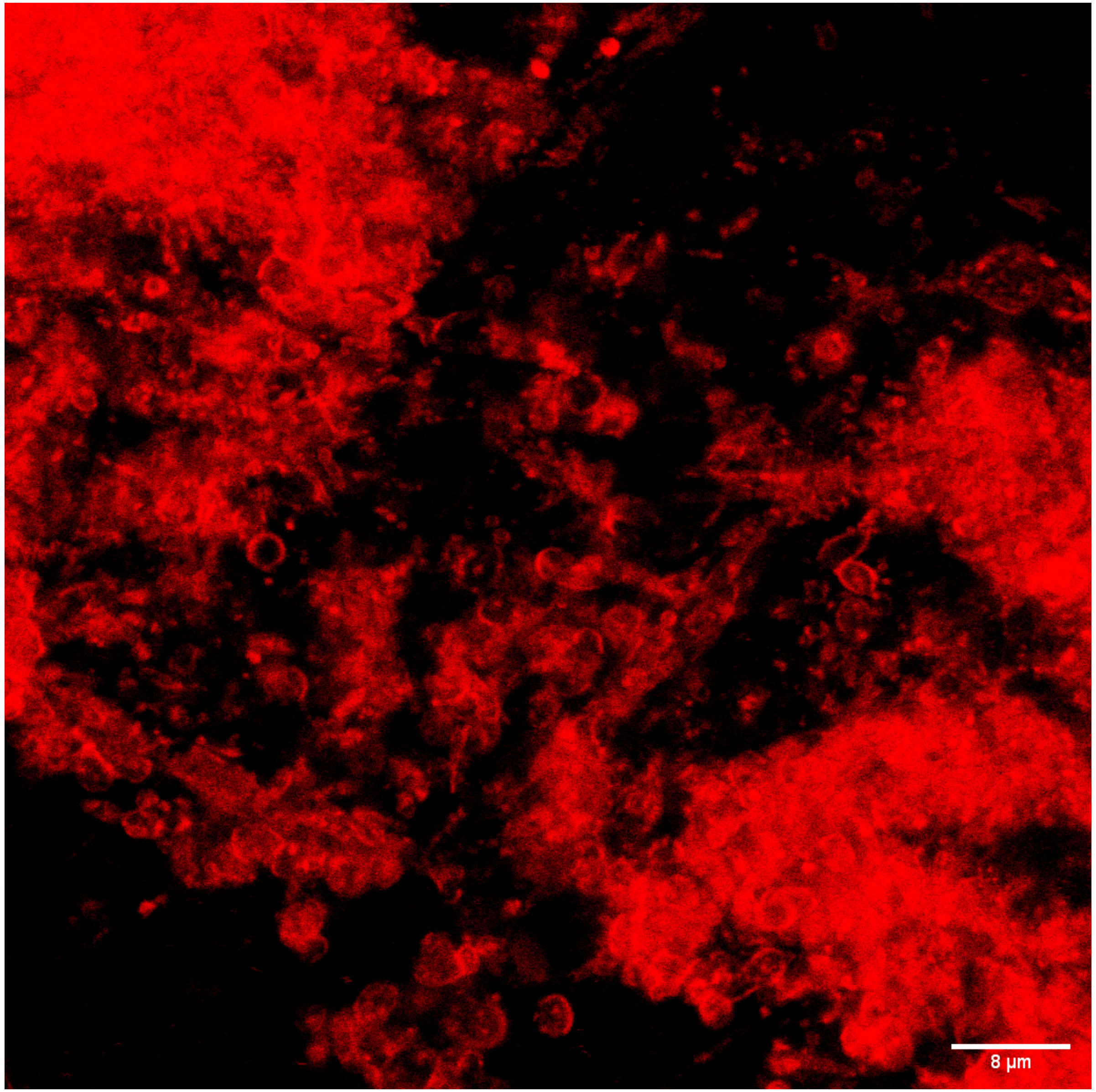

(c) Representative images used to calculate the membrane/cell wall ratio, as presented in the histogram in Fig. 2h.

Fig. 2f, g *sco1760::Tn5* mutant growing on GYM 0,64M sucrose 48-hour culture. WGA (green) and FM595 (red) staining.

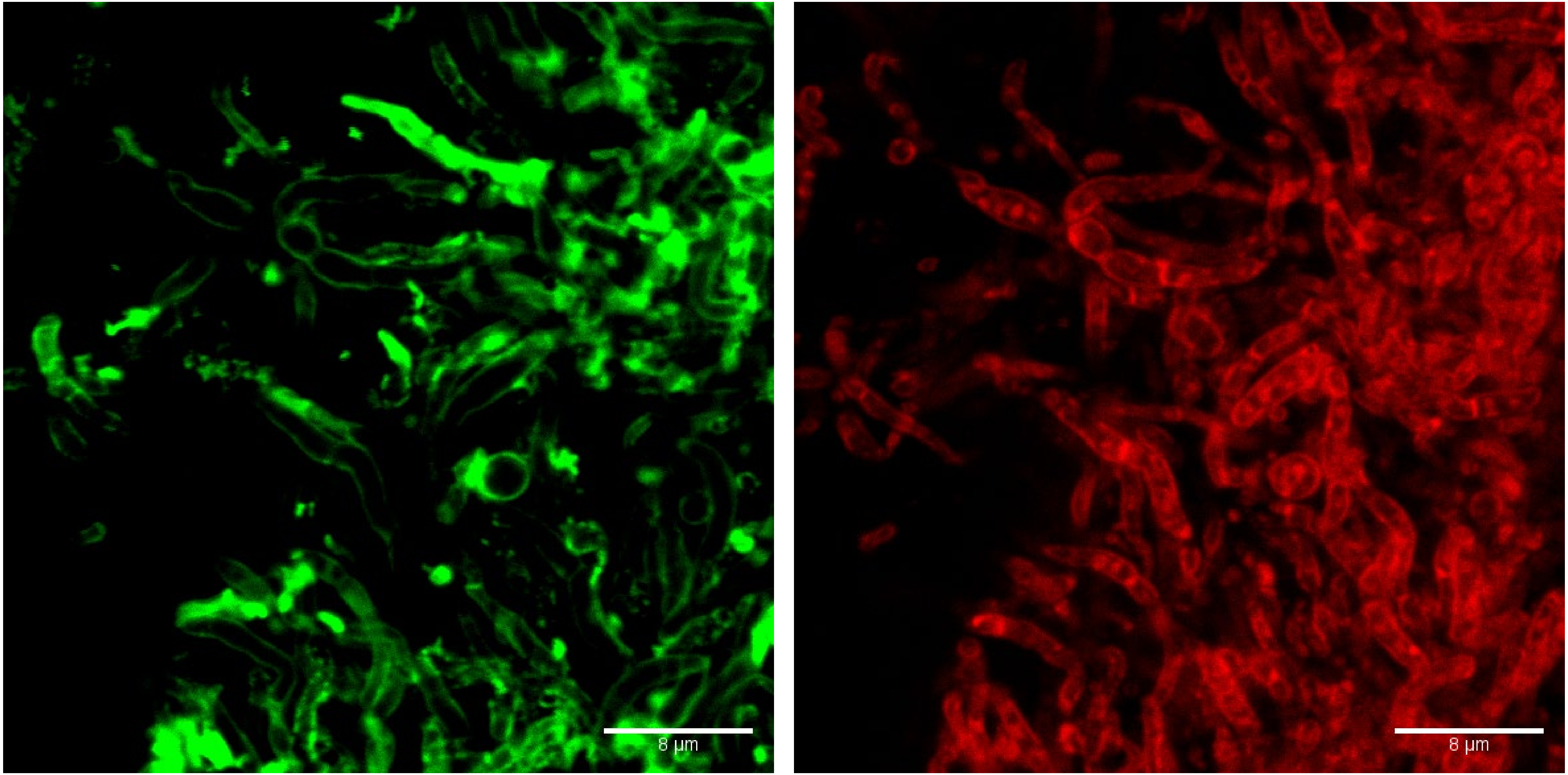

Supplement: Supplementary file 5 — Supplementary Fig. S1 [file 41598_2025_15457_MOESM5_ESM.pdf]
