# Supplementary material for: Peptidoglycan-reshuffling proteins SCO0954, SCO1758, SCO4439, and SCO4440 modulate the formation of wall-deficient cells in Streptomyces coelicolor under hyperosmotic sucrose stress
Source: Sci Rep. 2025 Sep 1;15:32112. doi: 10.1038/s41598-025-15457-z (PMC12402492; doi:10.1038/s41598-025-15457-z)

Supplementary Fig. S2. Loss of the acetyl group from NAM after acid hydrolysis. NAM without acid hydrolysis is indicated in blue. NAM after acid hydrolysis (4N HCl at 110ºC for 14 hours) is indicated in red.

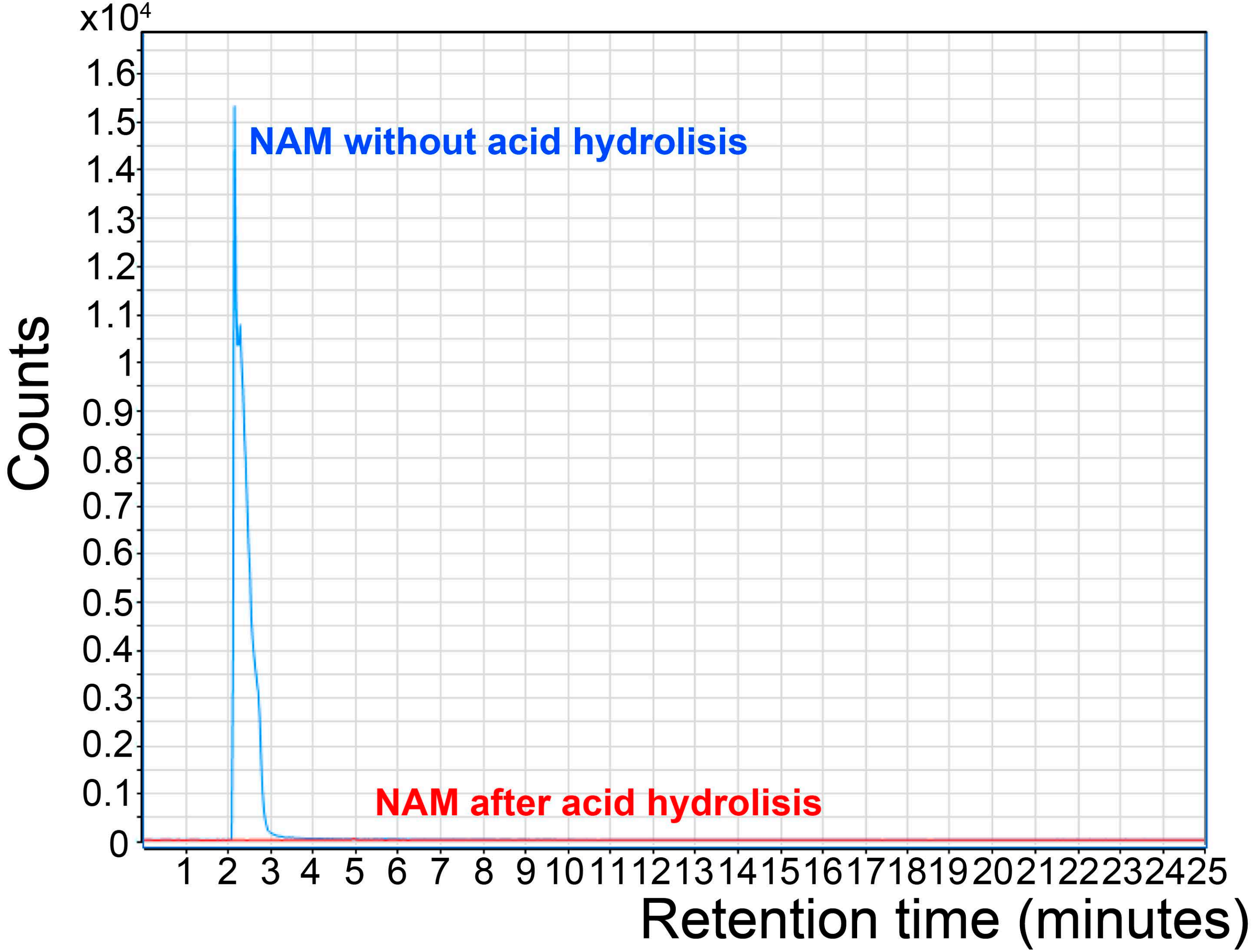

Supplement: Supplementary file 6 — Supplementary Fig. S2 [file 41598_2025_15457_MOESM6_ESM.pdf]
