# Supplementary material for: Peptidoglycan-reshuffling proteins SCO0954, SCO1758, SCO4439, and SCO4440 modulate the formation of wall-deficient cells in Streptomyces coelicolor under hyperosmotic sucrose stress
Source: Sci Rep. 2025 Sep 1;15:32112. doi: 10.1038/s41598-025-15457-z (PMC12402492; doi:10.1038/s41598-025-15457-z)
Supplement: Supplementary file 10 — Supplementary Movies S1-S3. [file 41598_2025_15457_MOESM10_ESM.pdf]

Supplementary Movies S1-S3. Time-lapse recordings of the *SCO1760::Tn5* mutant (Movie S1), the *S. coelicolor* wild-type strain overexpressing *SCO0954* (Movie S2), and *SCO4439/40::Tn5062* (Movie S3) growing on 0.64 M sucrose-supplemented GYM medium. Time-lapses were initiated in 48-hour cultures, once sucrose-driven hyperosmotic induced cells / EVs had formed. The cultures were stained with SYTO-9 (green, DNA stain), with images acquired every 13 minutes.
